# An unexpected clade of South American ground beetles (Coleoptera, Carabidae, *Bembidion*)

**DOI:** 10.3897/zookeys.416.7706

**Published:** 2014-06-17

**Authors:** David R. Maddison

**Affiliations:** 1 Department of Integrative Biology, Oregon State University, Corvallis, OR 97331, USA

**Keywords:** Carabidae, Bembidiini, *Bembidion*, phylogeny, systematics, DNA, South America, ground beetles

## Abstract

Phylogenetic relationships of the *Antiperyphanes* Complex of the genus *Bembidion* are inferred using DNA sequences from seven genes (two nuclear ribosomal, four nuclear protein coding, and one mitochondrial protein coding). Redefined subgenera within the complex are each well-supported as monophyletic. Most striking was the discovery that a small set of morphologically and ecologically heterogeneous species formed a clade, here called subgenus *Nothonepha*. This unexpected result was corroborated by the discovery of deep pits in the lateral body wall (in the mesepisternum) of all *Nothonepha*, a trait unique within *Bembidion*. These pits are filled with a waxy substance in ethanol-preserved specimens. In one newly discovered species (*Bembidion tetrapholeon* **sp. n.**, described here), these pits are so deep that their projections into the body cavity from the two sides touch each other internally. These structures in *Bembidion (Nothonepha)* are compared to very similar mesepisternal pits which have convergently evolved in two other groups of carabid beetles. The function of these thoracic pits is unknown. Most members of subgenus *Nothonepha* have in addition similar but smaller pits in the abdomen. A revised classification is proposed for the *Antiperyphanes* Complex.

## Introduction

Ground beetles of the genus *Bembidion* are distributed throughout the temperate regions of the world (Maddison 2012). The fauna of South America is diverse, with about 140 species described (Jeannel 1962; Toledano 2002; Toledano 2008), mostly occurring in cooler, southern regions of the continent, and northward in the Andes. Although many species resemble northern-hemisphere subgenera scattered throughout the two major clades of *Bembidion* (the *Bembidion* Series and the *Ocydromus* Series), all known species in South America are in fact members of only three groups within the *Bembidion* Series: the subgenera *Notaphus* and *Nothocys*, as well as the *Antiperyphanes* Complex (Maddison 2012).

**Figure 1.**
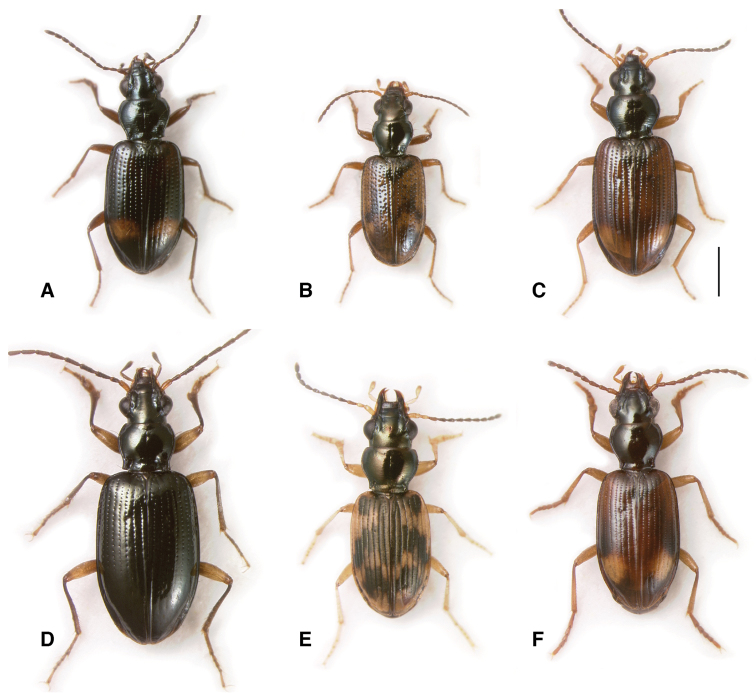
Adults of subgenera *Antiperyphanes* and *Chilioperyphus*. **A** *Bembidion (Antiperyphanes) rufoplagiatum*. Argentina: Neuquén: Arroyo Queñi at Lago Queñi, DRM voucher V100796 **B** *Bembidion (Antiperyphanes) hirtipes*, Argentina: Mendoza: Pampa Palauco, DRM voucher V100792 **C** *Bembidion (Antiperyphanes) spinolai*, Argentina: Chubut: Rio Azul at Lago Puelo, DRM voucher V100788 **D** *Bembidion (Antiperyphanes) zanettii*, Ecuador: Napo: Rio Quijos W of Baeza, DRM voucher V100791 **E** *Bembidion (Antiperyphanes) mandibulare*. Chile: Reg. X, Chiloé: Cucao, DRM voucher V100789 **F** *Bembidion (Chilioperyphus) orregoi*, Argentina: Chubut: Rio Azul at Lago Puelo, DRM voucher V100674. Scale bar 1 mm.

**Figure 2.**
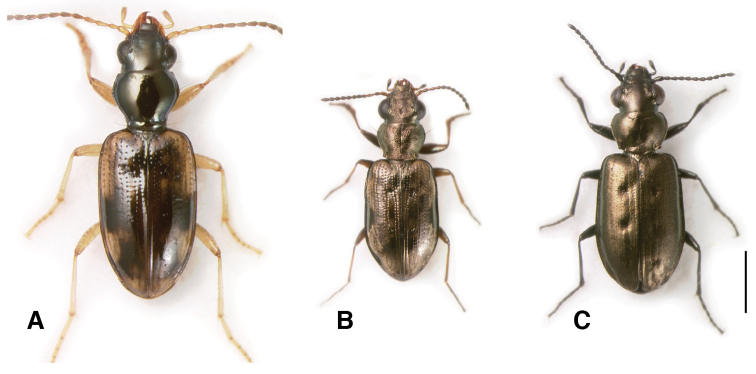
Adults of subgenera *Antiperyphus* and *Notholopha*. **A** *Bembidion (Antiperyphus) philippii*, Argentina: Neuquén: Rio Collón Curá ca 13 km S La Rinconada, DRM voucher V100787 **B** *Bembidion (Notholopha) scitulum*, CHILE: Reg. VII: Los Niches E of Curicó, DRM voucher V100790 **C** *Bembidion (Notholopha) sexfoveolatum*, CHILE: Reg. IX: 16.3 km E Malalcahuello, Cuesta Las Raices, DRM voucher V100598. Scale bar 1 mm.

**Figure 3.**
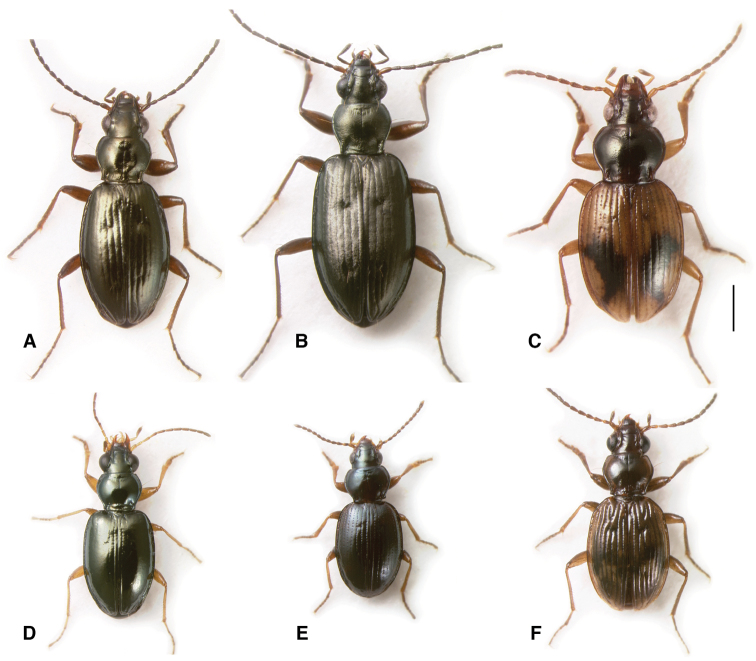
Adults of subgenus *Ecuadion*. **A** *Bembidion chimborazonum*, ECUADOR: Pichincha: Paso de la Virgen, DRM voucher V100793 **B** *Bembidion sanctaemarthae*, ECUADOR: Napo: Rio Chalpi Grande, DRM voucher V100798 **C** *Bembidion andersoni*, ECUADOR: Pichincha: Reserva Yanacocha, start Andean Snipe Trail, DRM voucher V100655 **D** *Bembidion walterrossi*, ECUADOR: Napo: Vinillos, 4.1 km S Cosanga, DRM voucher V100794 **E** *Bembidion cotopaxi*, ECUADOR: Pichincha: km 17 on route 28 W of Papallacta DRM voucher V100797 **F** *Bembidion ricei*, ECUADOR: Napo: Rio Chalpi Grande, DRM voucher V100622. Scale bar 1 mm.

**Figure 4.**
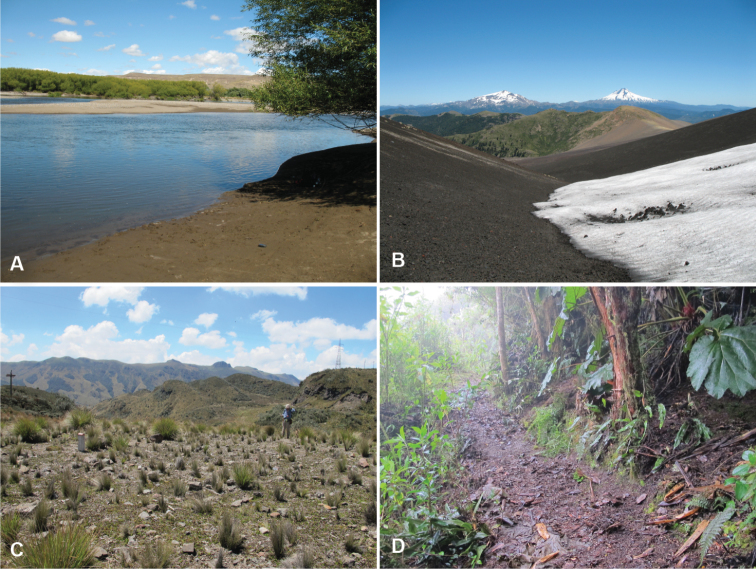
Habitats of the *Antiperyphanes* Complex. **A** River shore, Argentina: Neuquén: Rio Collón Curá, about 13 km S La Rinconada, 625m. On the sandy bank in the foreground *Bembidion philippii* is abundant, as is *Bembidion (Nothonepha)* sp. nr. *lonae*. Also occurring on the sand banks are *Bembidium orregoi* and *Bembidion (Nothonepha) eburneonigrum*. On the upper sand banks across the river are *Bembidium mandibulare*, and in the gravel are *Bembidium spinolai* **B** Edges of snowfields at Chile: Reg. IX: Volcán Lonquimay, 1910m. Habitat of *Bembidion (Notholopha) sexfoveolatum* **C** Open high-elevation grassland at Ecuador: Pichincha: Paso de la Virgen, 4060m, habitat of three species of subgenus *Ecuadion*: *Bembidion chimborazonum*, *Bembidion guamani*, and *Bembidion humboldti* **D** Leaf litter in cloud forest, Ecuador: Pichincha: Reserva Yanacocha, 0.1152°S, 78.5837°W, 3540m, habitat of *Bembidion andersoni*, *Bembidion georgeballi*, and *Bembidion onorei*.

The *Antiperyphanes* Complex is a clade restricted to South and Central America. Eight subgenera are considered to belong to the complex (Maddison 2012; Maddison et al. 2013): *Antiperyphanes* Jeannel, *Antiperyphus* Jeannel, *Chilioperyphus* Jeannel, *Plocamoperyphus* Jeannel, *Nothonepha* Jeannel, *Pacmophena* Jeannel, *Notholopha* Jeannel, and *Ecuadion* Moret and Toledano, with two subgenera suspected of belonging (*Notoperyphus* Bonniard de Saludo (1969), *Pseudotrepanes* Jeannel (1962)). Members of this complex are moderately diverse in form, and are typically shades of brown, orange, and yellow, although a few species have metallic colors (Figs 1–3) They are abundant along edges of bodies of water (rivers, creeks, ponds, lakes, snowfields, and ocean; Figs 4A, B) in south temperate regions (especially Argentina, Chile, Peru, and Bolivia), and at higher elevations from Patagonia north into Central America. In the mountains of Ecuador, Peru, and nearby areas, one group (subgenus *Ecuadion*) has radiated into alpine grasslands (Fig. 4C), cloud forest leaf litter (Fig. 4D), clay cliffs, and other habitats distant from open water.

Although monophyly of the *Antiperyphanes* Complex is well supported (Maddison 2012), details about its phylogenetic structure are poorly known. Only 20 species of the 95 or so known species have been included in phylogenetic studies, with some key taxa missing. For example, only two species of what is considered the heterogeneous subgenus *Antiperyphus* have been sampled, and its type species (*Bembidium philippii* Germain) has not previously been examined. Similarly, only two of the more than 50 species of *Ecuadion* were included in previous studies.

**Figure 5.**
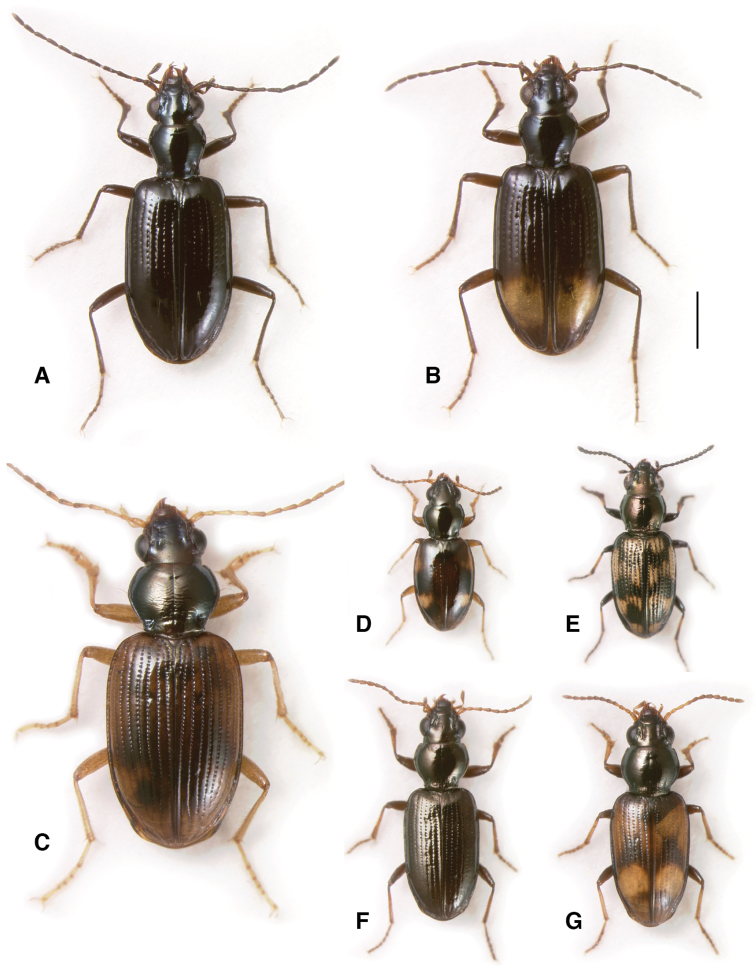
Adults of subgenus *Nothonepha*. **A** *Bembidion tetrapholeon* (black form), Argentina: Neuquén: Arroyo Queñi at Lago Queñi, DRM voucher V100781 **B** *Bembidion tetrapholeon* (orange form), Argentina: Chubut: Rio Azul at Lago Puelo, DRM voucher V100780 **C** *Bembidion germainianum*, Argentina: Neuquén: Rio Salado at route 40, DRM voucher V100782 **D** *Bembidion lonae*, Argentina: Mendoza: Salinas del Diamante, DRM voucher V100786 **E** *Bembidion eburneonigrum*, Argentina: Neuquén: Rio Neuquén at Chos Malal, DRM voucher V100785 **F** *Bembidion tucumanum*, Argentina: Mendoza: Salinas del Diamante, DRM voucher V100783 **G** *Bembidion engelhardti engelhardti*, Argentina: Neuquén: Rio Salado at route 40, DRM voucher V100784. Scale bar 1 mm.

**Figure 6.**
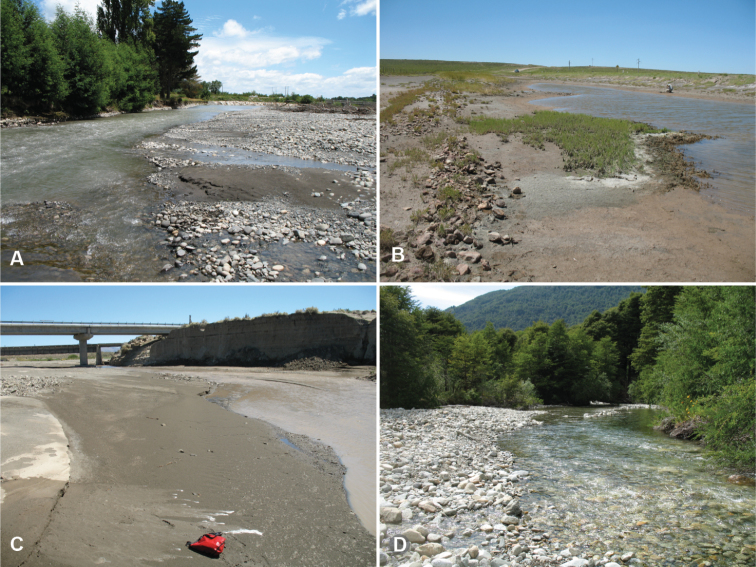
Habitats of the subgenus *Nothonepha*. **A** Habitat of *Bembidion (Nothonepha) eburneonigrum* (on sand patches) and *Bembidion (Nothonepha)* sp. nr. *lonae* (on sand patches and in gravel). This habitat (Chile: Reg. IX: Rio Allipén at route 119, 132m) is also home to *Bembidion spinolai* Solier and *Bembidion rufoplagiatum* **B** Habitat of *Bembidion (Nothonepha) tucumanum*, *Bembidion (Nothonepha) lonae*, and *Bembidion (Nothonepha) engelhardti* (Argentina: Mendoza: Salinas del Diamante, 1280m). The beetles are common under rocks and around vegetation on the salt-encrusted clay and sand banks of this saline pond; in the same habitat *Bembidion (Notaphus) cillenoides* Jensen-Haarup and two other *Notaphus* are common **C** On the sand shores of this desert river *Bembidion (Nothonepha) germainianum*, *Bembidion (Nothonepha) engelhardti*, and *Bembidion (Nothonepha) lonae* are common (Argentina: Neuquén: Rio Salado at route 40, 725m) **D** Type locality of *Bembidion (Nothonepha) tetrapholeon* (Argentina: Neuquén: Arroyo Queñi at Lago Queñi, 830m). The beetles are found under rocks along the river shore; *Bembidion rufoplagiatum* is also common in this habitat.

The current more in-depth investigation into phylogeny of the *Antiperyphanes* Complex was inspired by discovery, on the gravel shores of Rio Puntra on Isla Grande de Chiloé, Chile, of a large, distinctive, undescribed species of *Bembidion* (Figs 5A and 5B). This unusual species appeared to fall outside any named subgenus, and is given the name *Bembidion tetrapholeon* in this paper. In order to infer its relationships, additional members of the *Antiperyphanes* Complex were gathered and sequenced. Preliminary results from the sequences of one gene indicated the existence of a clade so surprising that I initially considered it fallacious, a result of errors in sample labeling, but when additional samples and genes provided stronger support, that explanation was no longer tenable. This apparent clade, including the new species, consisted of taxa that are much more diverse in form (Fig. 5) and habitat (Fig. 6) than other small clades of similar molecular diversity. This paper reports the results of sequencing of seven genes which together provide very strong support for this clade. The discovery of the clade led to the search for morphological synapomorphies of its members, and a striking, derived character was found in thoracic structure. Although the focus of the paper is on this unexpected clade, the relationships of other members of the *Antiperyphanes* Complex are explored, and a new classification is proposed for the group.

## Methods

Specimens examined and depositories. Specimens examined are from or will be deposited in the collections listed below. Each collection’s listing begins with the coden used in the text.

BMNH The Natural History Museum, London, UK

CMNH Carnegie Museum of Natural History, Pittsburgh, Pennsylvania, USA

CTVR Luca Toledano collection, Verona, Italy

EMEC Essig Museum Entomology Collection, University of California, Berkeley, USA

IADIZA Instituto Argentino de Investigaciones de las Zonas Aridas, Mendoza, Argentina

MACN Museo Argentino de Ciencias Naturales “Bernardino Rivadavia”, Buenos Aires, Argentina.

MNHN Muséum National d’Histoire Naturelle, Paris, France

MNNC Museo Nacional de Historia Natural, Santiago, Chile

NHMW Naturhistorisches Museum, Wien, Austria

OSAC Oregon State Arthropod Collection, Oregon State University, Corvallis, USA

USNM National Museum of Natural History, Washington, USA

ZMUC Natural History Museum of Denmark, University of Copenhagen, Copenhagen, Denmark

**Collecting methods.** Specimens were collected by hand or using an aspirator; specimens were found during the day in their habitat after splashing the soil with water, or with the aid of a headlamp at night, when the beetles are more actively moving on the surface.

Most specimens were killed and preserved in *Acer* sawdust to which ethyl acetate was added. Specimens collected specifically for DNA sequencing were killed and stored in 95% or 100% ethanol, with best results obtained when the abdomen was slightly separated from the rest of the body to allow better penetration, or when the reproductive system was dissected out through the rear of the abdomen within a few minutes of the beetle’s death in ethanol. Ethanol was decanted and refilled at least two times within the first few weeks after death. Storage was then at 4° or -20°C.

**Figure 7.**
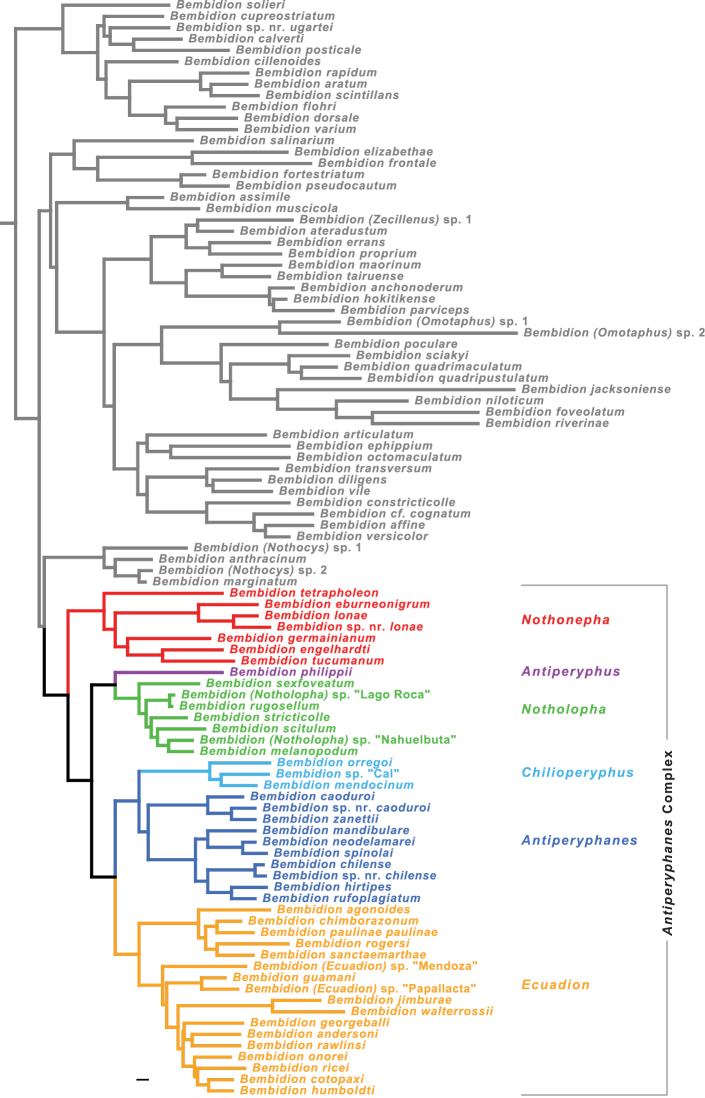
Tree of highest likelihood found for the combined, seven-gene matrix with partitioning scheme chosen by PartitionFinder. Only members of the *Bembidion* Series shown; more distant outgroups not depicted. Scale bar: 0.01, branch lengths as reconstructed by RAxML.

**Taxon sampling for DNA studies.** DNA was newly sequenced from 25 species of the *Antiperyphanes* Complex of *Bembidion*; to these sequences were added sequences of 20 more species of this complex acquired during previous studies (Table 1). Forty additional species of *Bembidion* were also included in the analyses (27 species of the *Bembidion* Series exclusive of the *Antiperyphanes* Complex, plus 13 species of *Bembidion* outside the *Bembidion* Series). The 27 additional *Bembidion* Series species are evident in Fig. 7; details about these species and specimens sequenced are available in Maddison (2012). The 13 species outside of the *Bembidion* Series included are *Bembidion variegatum* Say, *Bembidion planum* (Haldeman), *Bembidion stephensi* Crotch, *Bembidion tetracolum* Say, *Bembidion hastii* Sahlberg, *Bembidion punctulatum* Drapiez, *Bembidion properans* (Stephens), *Bembidion concolor* (Kirby), *Bembidion chalceum* (Dejean), *Bembidion lunulatum* (Geoffroy), *Bembidion wickhami* Hayward, *Bembidion genei illigeri* Netolitzky, and Bembidion cf. exquisitium Andrewes.

**Table 1.** Sampling of *Bembidion* species of the *Antiperyphanes* Complex. The ID column indicates how the specimens were identified: if there is a number in square brackets, then the specimen was identified by me using one of the following references: 1: Jeannel (1962); 2: Toledano (2008); 3: Vigna Taglianti and Toledano (2008); 4: Moret and Toledano (2002); 5: Erwin (1982). If there is a single letter, then the specimen is the holotype of the species (“H”), or I compared the specimen to the primary type or syntypes (“T”) of the species, in consultation with the relevant literature; the species marked “LT” was identified by Luca Toledano. Thus, all of these are “genseq-4” sequences (Chakrabarty et al. 2013), except for DNA2653 (genseq-1) and DNA2236 (genseq-3). In the third column, four-digit numbers in entries are D.R. Maddison voucher numbers for sequenced specimens; if a “*” appears in that column, the data from these specimens were obtained from GenBank from previous studies (Maddison 2012; Maddison and Ober 2011; Maddison and Toledano 2012; Maddison et al. 2013), and more data about the specimens is present in those papers. Locality details for newly sequenced specimens (those without a “*”) are given in the Appendix 1. For those three species with a “*” in the “#” column but without a number, details of specimens sequenced is available in Maddison (2012). For each gene, GenBank accession numbers are listed.

	ID	#	CAD	*wg*	ArgK	Topo	28S	18S	COI
**Subgenus *Antiperyphanes* Jeannel**									
*Bembidion caoduroi* Toledano	[2]	1987*	JN170771	JN171375	JN170525	JN171188	JN170305	JN170158	JN171006
*Bembidion* sp. nr. *caoduroi* Toledano		2677	KJ653108	KJ653211	KJ653079	KJ653177	KJ653045		KJ653141
*Bembidion chilense* Solier	[1]	1466*	JN170779	JN171382	JN170533	JN171196	JN170313		JN171014
*Bembidion* sp. nr. *chilense* Solier		*	JN170920	GU556037	JN170677	JN171297	GU556088	JN170236	JN171117
*Bembidium spinolai* Solier	T	*	JN170925	JN171523	JN170682	JN171302	JN170448		JN171122
*Bembidion neodelamarei* Toledano	[1]	2342	KJ653102	KJ653205	KJ653073	KJ653171	KJ653040		KJ653136
*Bembidion zanettii* Toledano	[2]	2679*	KC140267	KC140241	KC140273	KC140251	KC140281	KC140285	KC140261
*Bembidion hirtipes* (Jeannel)	T	2335*	JN170822	JN171424	JN170576	JN171227	JN170354		JN171045
*Bembidion rufoplagiatum* Germain	T	*	JN170902	JN171501	JN170659	JN171282	JN170426	JN170227	JN171102
*Bembidium mandibulare* Solier	[1]	2203*	EU677545	EU677669	JN170603	EU677643	EU677689	JN170200	JN171065
**Subgenus *Chilioperyphus* Jeannel**									
*Bembidium orregoi* Germain	T	2333*	KC140265	KC140238	KC140271	KC140246	KC140278	KC140283	KC140256
*Bembidion mendocinum* Jensen-Haarup	T	2337*	KC140266	KC140239	KC140272	KC140247	KC140279	KC140284	KC140257
*Bembidion* sp. n.“Cal”		2700*	KC140262	KC140234	KC140268	KC140243	KC140274	KC140282	KC140252
**Subgenus *Ecuadion* Moret & Toledano**									
*Bembidion agonoides* Vigna Taglianti & Toledano	[3]	2675	KJ653090	KJ653193	KJ653061	KJ653159	KJ653028	KJ653020	KJ653124
*Bembidion andersoni* Toledano	[2]	2651	KJ653091	KJ653194	KJ653062	KJ653160	KJ653029		KJ653125
*Bembidion chimborazonum* Moret & Toledano	[4]	2659	KJ653093	KJ653196	KJ653064	KJ653162	KJ653031		KJ653127
*Bembidion cotopaxi* Moret & Toledano	[4]	2658	KJ653094	KJ653197	KJ653065	KJ653163	KJ653032		KJ653128
*Bembidion georgeballi* Toledano	[2]	2661	KJ653097	KJ653200	KJ653068	KJ653166	KJ653035	KJ653022	KJ653131
*Bembidion guamani* Moret & Toledano	[4]	2660	KJ653099	KJ653202	KJ653070	KJ653168	KJ653037		KJ653133
*Bembidion humboldti* Moret & Toledano	[4]	2673	KJ653100	KJ653203	KJ653071	KJ653169	KJ653038		KJ653134
*Bembidion jimburae* Moret & Toledano	[4]	2674	KJ653101	KJ653204	KJ653072	KJ653170	KJ653039		KJ653135
*Bembidion onorei* Moret & Toledano	[4]	2678	KJ653103	KJ653206	KJ653074	KJ653172	KJ653041		KJ653137
*Bembidion paulinae paulinae* Moret & Toledano	[4]	2783	KJ653104	KJ653207	KJ653075	KJ653173	KJ653042		KJ653138
*Bembidion rawlinsi* Moret & Toledano	[4]	1462*	JN170893	JN171492	JN170650	JN171275	JN170418		JN171096
*Bembidion ricei* Maddison & Toledano	H	2653	KJ653106	KJ653209	KJ653077	KJ653175	JX971116		JX971117
*Bembidion rogersi* Bates	[5]	2414*	JN170897	JN171496	JN170654	JN171279	JN170422	JN170225	JN171100
*Bembidion sanctaemarthae* Darlington	[4]	2652	KJ653107	KJ653210	KJ653078	KJ653176	KJ653044		KJ653140
*Bembidion* sp. “Mendoza”		2701	KJ653088	KJ653191	KJ653059	KJ653157	KJ653026	KJ653019	KJ653122
*Bembidion* sp. “Papallacta”		2657	KJ653092	KJ653195	KJ653063	KJ653161	KJ653030		KJ653126
*Bembidion walterrossii* Toledano	[2]	2650	KJ653121	KJ653220	KJ653087	KJ653190	KJ653058		KJ653154
**Subgenus *Antiperyphus* Jeannel**									
*Bembidium philippii* Germain	T	2327	KJ653105	KJ653208	KJ653076	KJ653174	KJ653043	KJ653023	KJ653139
**Subgenus *Notholopha* Jeannel**									
*Bembidion rugosellum* (Jeannel)	LT	1348*	JN170903	JN171502	JN170660	JN171283	JN170427	JN170228	JN171103
*Bembidion sexfoveatum* Germain	T	2208*	JN170916	JN171515	JN170673	JN171293	JN170439	JN170233	JN171113
*Bembidion melanopodum* Solier	T	2307*	JN170853	JN171453	JN170609	JN171249	JN170383	JN170202	JN171069
*Bembidium scitulum* Erichson	[1]	1347*	JN170911	JN171510	JN170668	JN171288	JN170435		JN171109
*Bembidion stricticolle* Germain	T	2240	KJ653109	KJ653212	KJ653080	KJ653178	KJ653046		KJ653142
*Bembidion* sp. “Lago Roca”		2046*	JN170747	JN171352	JN170500	KC140249	JN170281		KC140259
*Bembidion* sp. “Nahuelbuta”		2239	KJ653089	KJ653192	KJ653060	KJ653158	KJ653027		KJ653123
**Subgenus *Nothonepha* Jeannel**									
*Bembidion eburneonigrum* Germain	T	2204	KJ653095	KJ653198	KJ653066	KJ653164	KJ653033	KJ653021	KJ653129
*Bembidion engelhardti* Jensen-Haarup	T	2334	KJ653096	KJ653199	KJ653067	KJ653165	KJ653034		KJ653130
*Bembidion germainianum* Toledano	T	2336	KJ653098	KJ653201	KJ653069	KJ653167	KJ653036		KJ653132
*Bembidion lonae* Jensen-Haarup	T	1321*	JN170844	JN171444	JN170599	JN171242	JN170374	JN170196	JN171061
*Bembidion* sp. nr. *lonae* Jensen-Haarup		1457*	JN170921	JN171519	JN170678	JN171298	JN170444		JN171118
*Bembidion tetrapholeon* sp. n.	T	2236	KJ653111	KJ653214	KJ653081	KJ653180	KJ653048	KJ653024	KJ653144
*Bembidion tucumanum* (Jeannel)	T	1430	KJ653120	KJ653219	KJ653086	KJ653189	KJ653057	KJ653025	KJ653153

Nine additional specimens of *Bembidion tetrapholeon* sp. n., were sequenced (Table 2) to examine variation. The 10 specimens sequenced in total included specimens from two localities in Chile and three localities in Argentina (Tables 1, 2), and included the typical uniformly black specimens, and others that have a large orange spot on their elytra.

**Table 2.** Additional sampling of *Bembidion tetrapholeon* to examine DNA sequence variation. The DNA voucher number is listed in the “#” column. Color of elytra: BL: nearly black; OR: black with large orange spot. * indicates the holotype. Further details about the localities are provided under the description of *Bembidion tetrapholeon* and in the Appendix 1. All of these are “genseq-2” sequences (Chakrabarty et al. 2013), except for DNA2356 (genseq-1) and DNA1752 (genseq-3).

#	Color	CAD	*wg*	ArgK	Topo	28S	COI
1752	BL	KJ653110	KJ653213		KJ653179	KJ653047	KJ653143
2356*	BL	KJ653112	KJ653215	KJ653082	KJ653181	KJ653049	KJ653145
2357	OR	KJ653113	KJ653216	KJ653083	KJ653182	KJ653050	KJ653146
2562	BL	KJ653115	KJ653217	KJ653084	KJ653184	KJ653052	KJ653148
2566	BL	KJ653119	KJ653218	KJ653085	KJ653188	KJ653056	KJ653152
2555	OR	KJ653114			KJ653183	KJ653051	KJ653147
2564	OR	KJ653117			KJ653186	KJ653054	KJ653150
2565	BL	KJ653118			KJ653187	KJ653055	KJ653151
2563	BL	KJ653116			KJ653185	KJ653053	KJ653149

Vouchers are housed in the David Maddison voucher collection at Oregon State University, with the exception of voucher number DNA2356, the holotype of *Bembidion tetrapholeon*, which is deposited in IADIZA.

**DNA sequencing.** The genes studied, and abbreviations used in this paper, are: **28S** or **28S rDNA**: 28S ribosomal DNA; **18S** or **18S rDNA**: 18S ribosomal DNA; **ArgK**: arginine kinase; **CAD**: carbamoyl phosphate synthetase domain of the *rudimentary* gene; **COI**: cytochrome oxidase I; **Topo**: topoisomerase I; ***wg***: *wingless*.

Fragments for these genes were amplified using the Polymerase Chain Reaction on an Eppendorf Mastercycler Thermal Cycler ProS, using TaKaRa Ex Taq and the basic protocols recommended by the manufacturer. Primers and details of the cycling reactions used are given in Maddison (2012). The amplified products were then cleaned, quantified, and sequenced at the University of Arizona’s Genomic and Technology Core Facility using a 3730 XL Applied Biosystems automatic sequencer.

Assembly of multiple chromatograms for each gene fragment and initial base calls were made with Phred (Green and Ewing 2002) and Phrap (Green 1999) as orchestrated by Mesquite’s Chromaseq package (Maddison and Maddison 2011a; Maddison and Maddison 2011b) with subsequent modifications by Chromaseq and manual inspection. Multiple peaks at a single position in multiple reads were coded using IUPAC ambiguity codes.

Sequences of COI for two species showed evidence of nuclear copies of this mitochondrial gene (“numts”) (Thalmann et al. 2004). For *Bembidion georgeballi* (voucher 2661) and *Bembidion walterrossii* (voucher 2650), sequences obtained using the LC1490-HC2198 primer pair yielded different sequences from the B1490-Bcoi2R primer pair. The former had numerous double-peaks, suggesting that the sequences included numts (Maddison 2012); the LC1490-HC2198 sequence for *Bembidion walterrossii* also had one stop codon near its 5’ end. The reads from the B1490-Bcoi2R primers are much cleaner and show no double-peaks. Although all of these sequences have been submitted to GenBank, only the sequences from the B1490-Bcoi2R primers have been included in the analyses.

**Alignment and data exclusion.** The appropriate alignment was obvious for all protein-coding genes. There were no insertion or deletions (indels) evident in the sampled CAD, ArgK, Topo, or COI sequences. In *wingless*, there were two small, well-separated indels, restricted to only three taxa: three inserted nucleotides in *Bembidion (Zemetallina) parviceps* Bates, and six inserted nucleotides in a different region in the two species of subgenus *Omotaphus* Netolitzky sampled. These inserted nucleotides were excluded from analyses.

The ribosomal genes showed a slightly more complex history of insertions and deletions. Both genes were first subjected to multiple sequence alignment in MAFFT version 7.130b (Katoh and Standley 2013), using the L-INS-i search option and otherwise default parameter values. Visual inspection suggested no needed improvements, and no ambiguously aligned regions that required exclusion.

**Molecular phylogenetic analysis.** Models of nucleotide evolution were chosen with the aid of jModelTest version 2.1.1 (Darriba et al. 2012; Guindon and Gascuel 2003) (for each gene) and PartitionFinder version 1.1.1 (Lanfear et al. 2012) (for parts of the partition chosen by PartitionFinder). Among the models supported by RAxML, the model chosen for all genes by the Bayesian Information Criterion was GTR+I+Γ.

Likelihood analyses of nucleotide data were conducted using RAxML version 7.2.6 (Stamatakis 2006). Analyses were conducted on each gene individually, as well as a matrix of seven genes concatenated together. Two different partitioning schemes were examined: (1) with seven parts, one for each gene; (2) as chosen using the Bayesian Information Criterion (BIC) using PartitionFinder (Lanfear et al. 2012). The partition chosen by BIC contained three parts: one part with third positions of COI; a second part with third positions of the nuclear protein-coding genes; a third part with all remaining sites. For bootstrap analyses 2000 replicates were conducted; maximum likelihood bootstrap (MLB) values are reported as percentages. In addition to these bootstrap analyses, searches for maximum likelihood trees were conducted using 1000 search replicates.

Most-parsimonious trees (MPTs) were sought using PAUP* (Swofford 2002). To search for most parsimonious trees, 2000 replicates were conducted, each beginning with a starting tree formed with the random addition sequence option, with each replicate saving no more than 25 trees. For parsimony bootstrap analyses in PAUP*, 1000 bootstrap replicates were examined, each of which used a heuristic search with four replicates, each beginning with a starting tree formed by the random addition sequence option, with TBR branch rearrangement, with each replicate saving no more than 25 trees; the estimated bootstrap values are reported as parsimony bootstrap percentages (PB).

**Morphological methods.** General methods of specimen preparation for morphological work, and terms used, are given in Maddison (1993; 2008). After dissection from the body, genitalia were prepared by treatment in 10% KOH at 65 °C for 10 minutes followed by multi-hour baths of distilled water, 5% glacial acetic acid, distilled water, and then ethanol. Male genitalia, when studied, have been mounted in Euparal between two small coverslips attached to archival-quality heavyweight watercolor paper. Measurements for body length are from apex of the labrum to apex of the longer elytron.

An examination of external and internal features of the exoskeleton was conducted to search for possible apomorphies of one of the discovered clades, focusing on externally-visible pits in the mesothorax and anterior region of the abdomen. Internal skeletal elements were studied on specimens whose soft tissue was dissolved by placing the opened body in a 10% KOH solution at 65 °C for 10 minutes.

Studies of muscles and other internal soft tissue were conducted on specimens killed and preserved in 100% ethanol. This is not the ideal preservation medium, as it leaves muscles brittle and more difficult to trace. However, since the unexpected discovery of structures requiring internal examination, better-fixed specimens have not been available.

Photographs of body parts were taken with a Leica Z6 and JVC KY-F75U camera. For pronotal, elytral, and genitalic images, a stack of photographs at different focal planes was taken using Microvision’s Cartograph software; these TIFF images were then merged using the PMax procedure in Zerene Systems’s Zerene Stacker; the final images thus potentially have some artifacts caused by the merging algorithm.

## Data resources

Sequences have been deposited in GenBank with accession numbers KJ653019 through KJ653220. GenBank numbers for the two apparent numts sequences are KJ653155 for DNA2661 and KJ653156 for DNA2650. Aligned data for each specimen as well as files containing inferred trees for each gene and concatenated matrices are available in Suppl. material 1 and 2, and have been deposited in the Dryad Digital Repository, http://doi.org/10.5061/dryad.47r16.

## Results

### Molecular results

**Figure 8.**
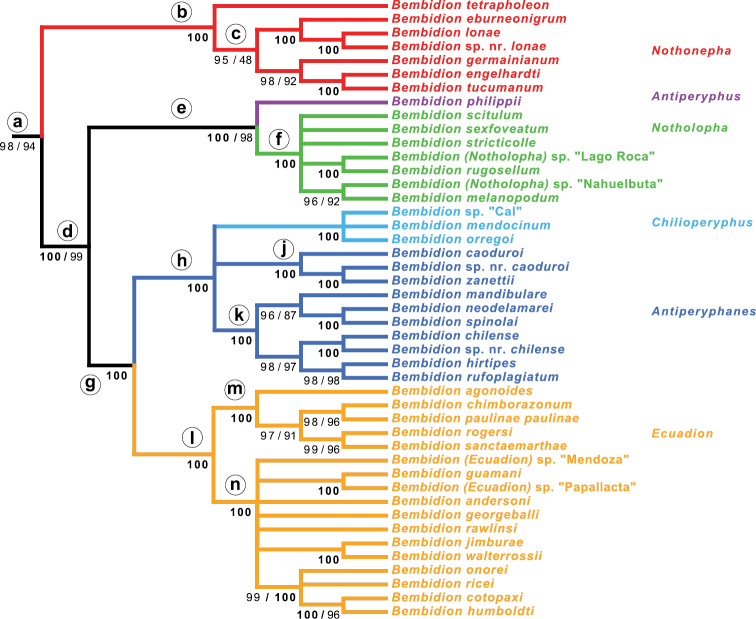
Maximum likelihood bootstrap tree showing only those clades appearing in 90% of the bootstrap replicates; taxa outside of the *Antiperyphanes* Complex not shown. Numbers below branches indicate maximum likelihood bootstrap percentage / parsimony bootstrap percentage. Circled letters on branches correspond to groups documented in Table 3.

The inferred phylogeny is presented in Figs 7 and 8, with support values for notable clades given in Table 3. Maximum likelihood trees and maximum likelihood bootstrap trees for each gene and the concatenated matrices are illustrated in Suppl. material 3 and 4. They are also contained in the NEXUS file S1 in the Suppl. material 1.

**Table 3.** Support for and against various clades. The letter at the left corresponds to the circled letters of clades in Fig. 8 (although clade **i**, consisting of clades **j** and **k**, is absent in Fig. 8). ML: Maximum likelihood analysis; P: parsimony analysis. Numbers indicate the bootstrap support expressed as a percentage. Cells shaded in gray to black indicate that the clade is present with bootstrap support greater than 50 or present in the optimal (maximum likelihood or most parsimonious) trees but with bootstrap value below 50. Cells in white indicate that the clade has a bootstrap value less than 50 and is not present in the optimal tree; if the bootstrap value is listed in parentheses, it means that a contradictory clade was present in the optimal trees. For no analysis was there bootstrap support greater than 50 against any of these clades. Abbreviations: “exc.” = “excluding”.

	Clade	Combined		CAD		wg		ArgK		Topo		28S		18S		COI	
		ML	P	ML	P	ML	P	ML	P	ML	P	ML	P	ML	P	ML	P
**a**	*Antiperyphanes* Complex	**98**	**94**	**88**	**86**	**27**	**19**	**(4)**	**1**	**(1)**	**0**	**(0)**	**0**	**(2)**	**1**	**(0)**	**(0)**
**b**	*Nothonepha*	**100**	**100**	**97**	**92**	**69**	**70**	**(1)**	**0**	**(2)**	**1**	**50**	**34**	**79**	**59**	**(0)**	**(0)**
**c**	*Nothonepha* exc. *Bembidion tetrapholeon*	**95**	**(48)**	**81**	**71**	**55**	**62**	**42**	**43**	**(1)**	**1**	**(9)**	**7**	**46**	**40**	**(0)**	**(0)**
**d**	*Antiperyphanes* Complex exc. *Nothonepha*	**100**	**99**	**80**	**79**	**(15)**	**20**	**38**	**22**	**(3)**	**9**	**81**	**75**	**54**	**23**	**(0)**	**0**
**e**	*Antiperyphus* + *Notholopha*	**100**	**98**	**98**	**98**	**78**	**70**	**8**	**2**	**(20)**	**20**	**(1)**	**3**	**(8)**	**46**	**(0)**	**(0)**
**f**	*Notholopha*	**100**	**100**	**63**	**80**	**83**	**81**	**16**	**43**	**43**	**53**	**(2)**	**3**	**72**	**68**	**49**	**63**
**g**	*Antiperyphanes* + *Chilioperyphus* + *Ecuadion*	**100**	**100**	**97**	**97**	**22**	**32**	**(1)**	**0**	**(29)**	**45**	**65**	**53**	**65**	**34**	**(0)**	**0**
**h**	*Antiperyphanes* + *Chilioperyphus*	**100**	**100**	**99**	**99**	**48**	**57**	**(7)**	**3**	**61**	**60**	**65**	**71**	**87**	**70**	**(0)**	**0**
**i**	*Antiperyphanes*	**68**	**71**	**54**	**59**	**(15)**	**16**	**(6)**	**7**	**33**	**58**	**68**	**73**	**75**	**62**	**(0)**	**0**
**j**	*Bembidion caoduroi* group	**100**	**100**	**100**	**100**	**95**	**96**	**98**	**98**	**96**	**96**	**93**	**92**	**66**	**57**	**62**	**57**
**k**	*Antiperyphanes* exc. *Bembidion caoduroi* group	**100**	**100**	**99**	**100**	**96**	**97**	**32**	**32**	**61**	**77**	**94**	**91**	**(38)**	**34**	**(29)**	**12**
**l**	*Ecuadion*	**100**	**100**	**54**	**46**	**47**	**53**	**44**	**33**	**66**	**59**	**99**	**100**	**55**	**43**	**(2)**	**0**
**m**	*Bembidion chimborazonum* group	**100**	**100**	**100**	**100**	**53**	**56**	**95**	**95**	**97**	**93**	**68**	**68**	**93**	**90**	**17**	**11**
**n**	*Ecuadion* exc. *Bembidion chimborazonum* group	**100**	**100**	**46**	**33**	**(38)**	**5**	**(23)**	**21**	**46**	**39**	**99**	**99**	**89**	**83**	**(1)**	**0**

The monophyly of each subgenus (indicated by color in Figs 7 and 8) is well supported by analyses of the concatenated matrix (MLB=100 and PB=100 for all but one subgenus) and at least four genes (Table 3), except for subgenus *Antiperyphanes*. *Antiperyphanes* is monophyletic in the maximum likelihood trees of four genes, but bootstrap support is low (Table 3).

The basal split in the *Antiperyphanes* Complex appears to be between clade **b** and clade **d** (Fig. 8). Clade **b** is strongly supported in seven-gene analyses (MLB=100, PB=100), and there is bootstrap support in individual analyses of four genes (Table 3). As a subgenus of *Bembidion*, this clade would take the name *Nothonepha* Jeannel, as it contains *Bembidion lonae*, the type species of *Nothonepha*. Clade **d** is also strongly supported in the seven-gene analyses (MLB=100, PB=99), and there is moderate to weak bootstrap support from four genes (Table 3).

Within the *Antiperyphanes* Complex, strongly supported relationships between subgenera include a clade containing *Antiperyphanes* and *Chilioperyphus*, and a sister-group relationship between that clade and the subgenus *Ecuadion* (Fig. 8, Table 3). *Bembidion (Antiperyphus) philippii* appears as the sister group to subgenus *Notholopha*.

As a whole, the *Antiperyphanes* Complex is supported as monophyletic (clade **a** in Figs 7 and 8, Table 3), although not as strongly as in an earlier study with more limited sampling of the group (Maddison 2012). Individual gene support for the clade is only provided by CAD and to a lesser extent *wingless* (Table 3), but the concatenated analyses have MLB=88 and PB=86. Lack of monophyly of the complex in some analyses (e.g., individual gene analyses of 28S and 18S) is a result of *Nothonepha* falling in the *Bembidion* Series separate from the rest of the complex.

Different analytical methods yielded similar results for the concatenated, seven-gene matrices. The two partition schemes examined (by gene and as chosen by PartitionFinder) resulted in maximum likelihood trees that differ only in the placement of *Bembidion georgeballi* within subgenus *Ecuadion*. In maximum likelihood bootstrap analyses, clades with MLB>90 were the same in both partition schemes. Parsimony analyses showed similar results to maximum likelihood (Table 3).

Within *Bembidion tetrapholeon* sp. n., the 10 specimens sequenced from five localities showed little variation in DNA, and the variation observed was not correlated with presence of an orange spot. There was no variation observed in CAD (n=10), ArgK (n=5), Topo (n=10), and 28S (n=10), over a total of more than 3090 bases. In the *wingless* gene (n=6) variation was observed at three third-position sites, all of which represent synonymous differences, and for each of which some other specimens were heterozygous for the variants. COI showed the most variation with variability at eight sites, seven of which represented synonymous differences and one a non-synonymous difference. At seven of these sites, nine of the ten specimens had the same nucleotide, with the tenth specimen being unique; the specimen that was unique varied from site to site. The most distinct specimen was DNA2236, from Chiloé, with three unique nucleotides in the more than 650 bases of COI sequenced.

### Morphological results

With the unexpected discovery that *Bembidion tetrapholeon* sp. n., belongs in a clade with an assortment of morphologically and ecologically diverse *Bembidion*, the search for synapomorphies for this clade became compelling.

**Figure 9.**
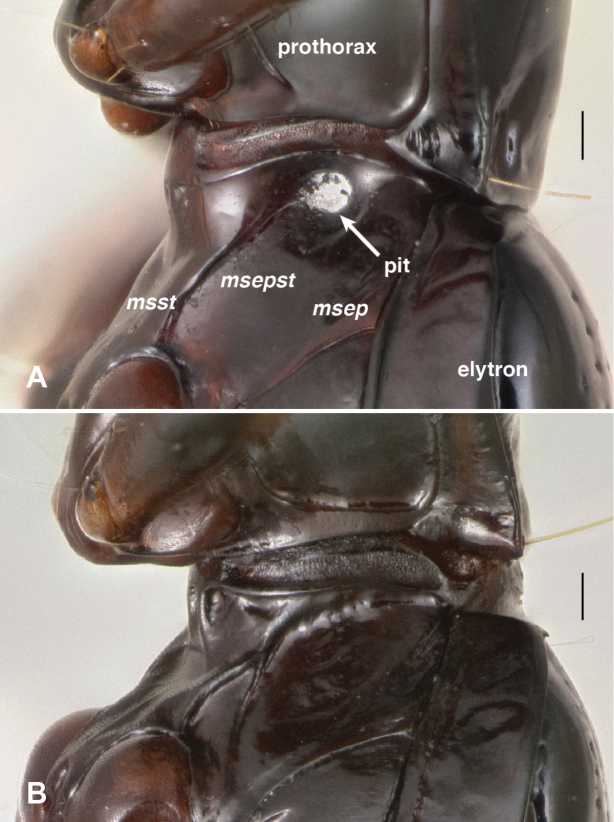
Left lateral region of the prothorax and mesothorax; top of each photograph is anterior. *msst*: mesosternum; *msepst*: mesepisternum; *msep*: mesepimeron (note that the boundaries between these sclerites are not evident externally in **A. A** *Bembidion (Nothonepha) tetrapholeon*, DRM voucher V100810 **B** *Bembidion (Antiperyphanes) zanettii*, DRM voucher V100811. Scale bar 0.1 mm.

**Mesothoracic pits.** The most striking derived feature observed was presence in all *Nothonepha* species of a pit in each lateral wall of the mesothorax. This mesepisternal pit (Fig. 9A) appears empty in many specimens killed in ethyl acetate, but in most specimens preserved in ethanol, a waxy substance is visible within it (Fig. 9A). When extracted and placed in glycerin on a microscope slide, this substance appears slightly yellowish-gray and contains no obvious substructure or particles (including no evident bacteria or fungal spores) when examined at 400× with transmitted, brightfield light (n=2, from *Bembidion tetrapholeon*). In contrast, all other members of *Bembidion* examined to date lack such a pit (e.g., Fig. 9B).

**Figure 10.**
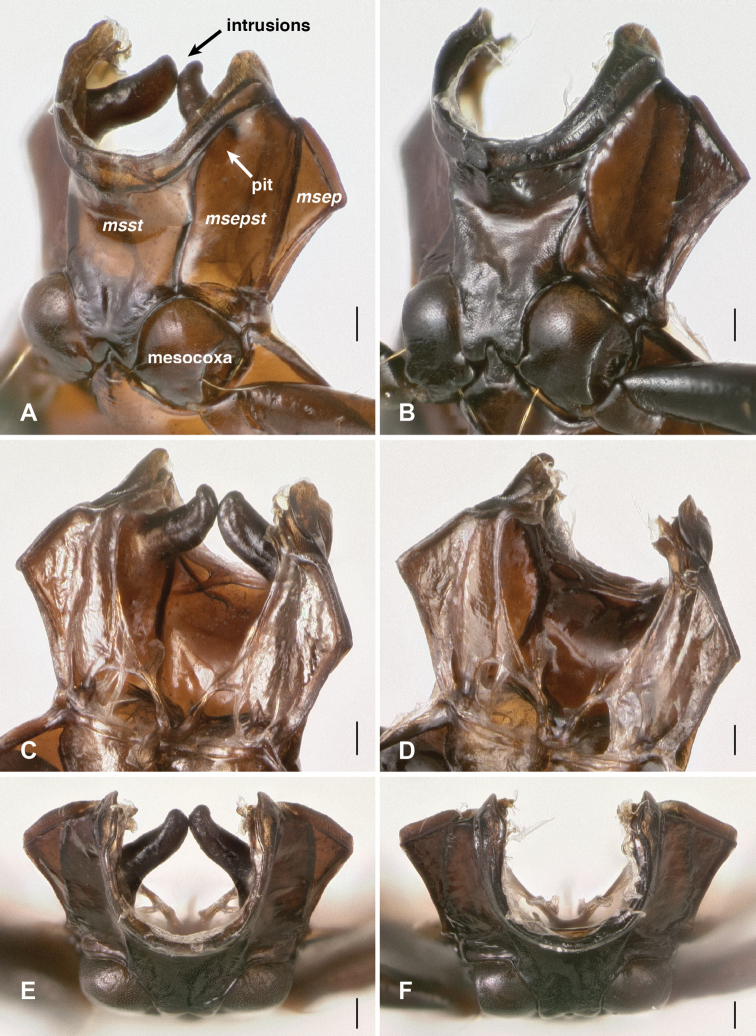
Mesothorax, dorsal surface and soft tissue removed. *msst*: mesosternum; *msepst*: mesepisternum; *msep*: mesepimeron. Scale bar 0.1 mm. (**A, C** and **E**) *Bembidion (Nothonepha) tetrapholeon*, DRM voucher V100766 **B, D**, and **F** *Bembidion (Antiperyphanes) zanettii*, DRM voucher V100767 **A, B** oblique ventral view; view from lower left side, slightly in front of mesothorax. **C, D** oblique dorsal view; view from upper right side, slightly behind the mesothorax. **E, F** anterior view.

**Figure 11.**
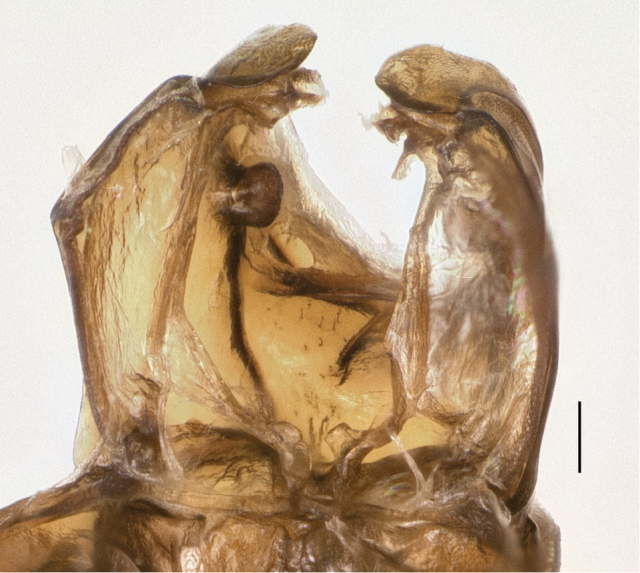
Mesothorax, dorsal surface and soft tissue removed, of *Bembidion (Nothonepha) engelhardti rayoda* Toledano. Oblique dorsal view; view from upper right side, slightly behind the mesothorax. Scale bar 0.1 mm.

In *Bembidion tetrapholeon* these paired structures, one on either side, internally manifest as large intrusions which touch in the center of the body cavity (Figs 10A, C, E). Typical *Bembidion* have no such structures internally (Figs 10B, D, F). There is variation within *Nothonepha* in the size of the intrusions, with *Bembidion engelhardti* having relatively small intrusions (and thus relatively shallow pits) (Fig. 11).

Examination of musculature in *Bembidion tetrapholeon* (n=4) and *Bembidion tucumanum* (n=1) revealed no muscles attached to the internal intrusions, although the course of some muscles appeared to be bent by the necessity to wrap around the structures. There were no evident large glands associated with the intrusions, although there were small patches of tissue on their internal surfaces.

**Figure 12.**
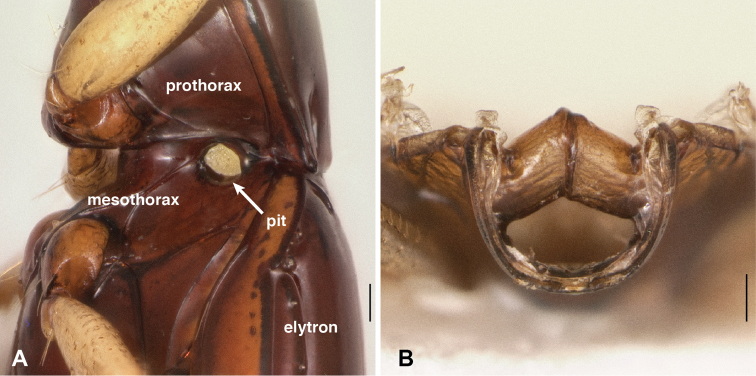
Mesothoracic structures of *Elaphropus (Tachylopha) leleupi* Basilewsky. **A** Left lateral region of the prothorax and mesothorax **B** Anterior view of mesothorax, dorsal surface and soft tissue removed. Scale bars 0.1 mm.

Two other groups of carabids reported to have mesepisternal pits were also examined, members of subgenus *Tachylopha* of the genus *Elaphropus* (Bruneau de Miré 1966; Erwin 1970) and the genus *Oodinus* (Spence 1982). I have specimens of these preserved in 95% ethanol, and both have large pits in the mesepisternum in the same place as *Bembidion (Nothonepha)*. In *Elaphropus (Tachylopha) leleupi* Basilewsky from South Africa the pits are filled with a waxy substance similar to that seen in *Nothonepha* (Fig. 12A). Internally these pits appear as two large intrusions that join in mid-thorax to form a tunnel (Fig. 12B); in the two specimens I have dissected there is no evidence of a septum at the point of joining, and the waxy substance fills the tunnel. I have seen ethanol-preserved specimens of *Elaphropus (Tachylopha) basilewskyi* Bruneau de Miré from Gabon (identified with Bruneau de Miré (1966)) and *Elaphropus (Tachylopha) spenceri* (Sloane) from Australia (identified with Baehr (1988)) that have similar pits also filled with a waxy substance. The apparently related subgenus *Sphaerotachys* also has mesepisternal pits, although they are much smaller than those seen in *Tachylopha*; a specimen I have examined from Hans Merensky Nature Reserve, Republic of South Africa, has pits similar to those shown in Fig. 11. The single ethanol-preserved *Oodinus alutaceus* (Bates) (identified with Bousquet (1996)) that I have examined, from south Texas, also has mesepisternal pits, but internally the intrusions do not touch, and are more similar in structure to those of *Bembidion (Nothonepha)* than *Elaphropus (Tachylopha)*﻿. In some specimens of *Oodinus*, the pits are also filled with a waxy substance (Spence 1982).

**Figure 13.**
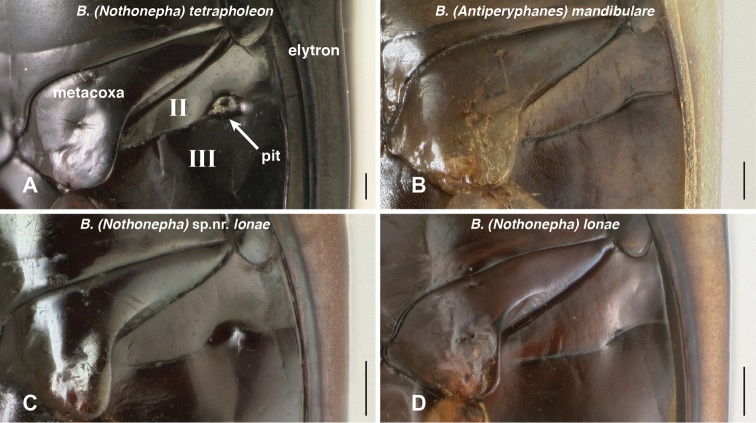
Ventral surface of anterior end of abdomen and posterior region of metathorax. The second and third abdominal segments are marked by II and III. **A** *Bembidion tetrapholeon*, DRM voucher V100773. **B** *Bembidium mandibulare*, DRM voucher V100772 **C** *Bembidion* sp. nr. *lonae*, DRM voucher V100771 **D** *Bembidion lonae*, DRM voucher V100770. Scale bar 0.1 mm.

**Abdominal pits.** In additional to mesepisternal pits, *Bembidion tetrapholeon* has a prominent pit on each side of the abdomen, ventrally, between abdominal segments II and III (Fig. 13A). In ethanol-preserved specimens, this pit is filled with a waxy substance similar to that in the mesepisternal pits. Almost all other species of subgenus *Nothonepha* have similar pits (e.g., *Bembidion (Nothonepha)* sp. nr. *lonae*, Fig. 13C); they are lacking only in *Bembidion (Nothonepha) lonae* (Fig. 13D), the sister to *Bembidion* sp. nr. *lonae*.

**Figure 14.**
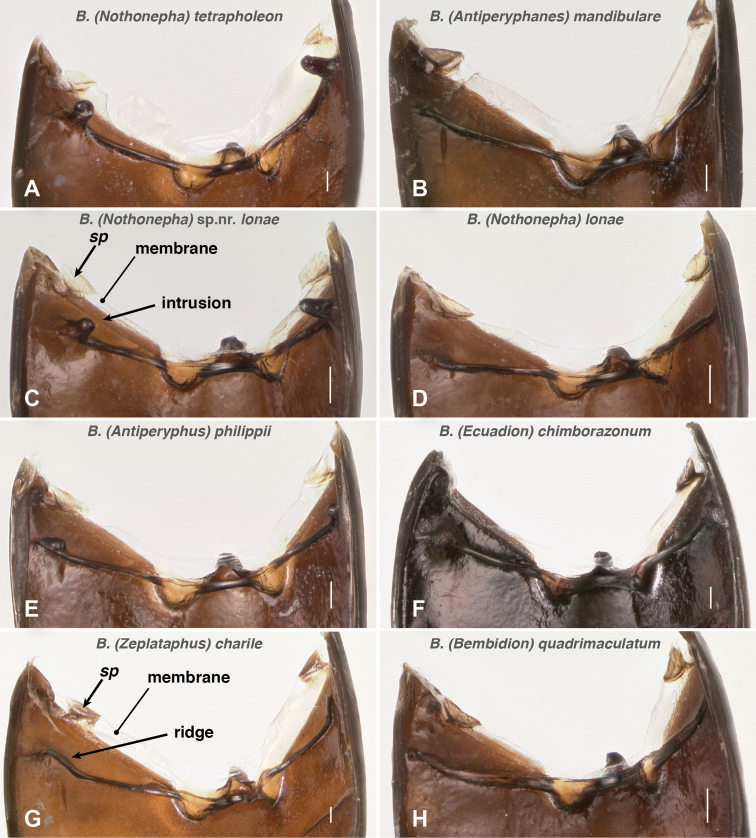
Inner surface of the anterior portion of the abdomen, soft tissue removed. The abdomen is slightly tilted to the left. *sp*: sclerotized patch on membrane that bounds the front of the abdomen on the ventral side. All species shown are members of the *Bembidion* Series; **A–F** are members of the *Antiperyphanes* Complex; **A, C** and **D** are members of the subgenus *Nothonepha* **A** *Bembidion tetrapholeon*, DRM voucher V100766 **B** *Bembidium mandibulare*, DRM voucher V100805 **C** *Bembidion* sp. nr. *lonae*, DRM voucher V100803 **D** *Bembidion lonae*, DRM voucher V100802 **E** *Bembidium philippii*, DRM voucher V100804 **F** *Bembidion chimborazonum*, DRM voucher V100809 **G** *Bembidion charile* Bates, DRM voucher DNA1171 **H** *Bembidion quadrimaculatum oppositum* Say, DRM voucher V100807. Scale bar 0.1 mm.

Internally these abdominal pits are evident as knob-shaped intrusions (Fig. 14A, C). Consistent with the lack of externally visible pits, *Bembidion lonae* lacks these intrusions, and has instead only a ridge between the abdominal segments (Fig. 14D), as is typical in *Bembidion*.

Outside of *Nothonepha* I have seen no species of *Bembidion* with as prominent abdominal pits, and most species lack them entirely. *Bembidion (Antiperyphanes) mandibulare*, for example, lacks abdominal pits and has only a slight linear depression in that region (Fig. 13B), and internally a simple ridge is evident (Fig. 14B). There is much variation between members of the *Antiperyphanes* Complex in this feature, however, with some species having an evident pit/internal intrusion (e.g., *Bembidion (Antiperyphus) philippii*, Fig. 14E, and *Bembidion (Antiperyphanes) zanettii*), and others (e.g., *Bembidion (Ecuadion) chimborazonum*, Fig. 14F) having a low, wide hump internally. Outside of the *Antiperyphanes* Complex all species examined either have a sinuate (Fig. 14G) or straight (Fig. 14H) ridge internally in this region.

Whatever internal structure is present between abdominal segments II and III, whether a ridge, or low mound, or knob-like intrusion, in the species examined this structure serves (at least in part) as an apodeme. In *Bembidion tetrapholeon* (n=5), for example, a muscle bundle is attached to the apex of the internal intrusion, and extends forward to the small sclerotized patch (*sp* in Fig. 14C) in the membrane that serves as the front boundary above the ventral margin of the abdomen. Another muscle bundle extends from this sclerotized patch forward and laterally to the lateral wall of the body, where it attaches to an external rod-like sclerite that is connected to the posterior lateral corner of the metanotum; this rod-like sclerite extends posterior laterally from that point to near the metepimeron. I have examined *Bembidion (Ecuadion) chimborazonum* (n=1) and it has a similar muscle attached to the low mound in the same region; *Bembidion (Bracteon) foveum* Motschulsky (n=1), *Bembidion* (*Ocydromus* Complex) *nebraskense* (n=2), and the bembidiine *Lionepha casta* (n=2) all have a similar muscle connecting the intersegmental ridge at the equivalent region to the small sclerotized patch.

## Discussion

**Monophyly of *Nothonepha*.** DNA data strongly support *Nothonepha* as a clade. Four genes (CAD, *wg*, 28S, 18S) independently have bootstrap support for the clade (Table 3), and the concatenated seven-gene analysis has MLB=100 and PB=100. Combined with the striking synapomorphy of mesothoracic pits, evidence for this clade becomes convincing. As Darwin (1859) noted, “We may err in this respect in regard to single points of structure, but when several characters, let them be ever so trifling, occur together throughout a large group of beings having different habits, we may feel almost sure, on the theory of descent, that these characters have been inherited from a common ancestor.”

**The unexpectedness of *Nothonepha*.** The relationship between species here grouped into subgenus *Nothonepha* was so unexpected when first discovered from sequences of 28S that it was dismissed, and considered to be the result of DNA contamination or mislabeled extractions. This small clade, of only ten known species, includes some of the largest *Bembidion* in South America (*Bembidion germainianum*, up to 6.4 mm in length, Fig. 5C), and some of the smallest (*Bembidion lonae*, down to 2.4 mm in length, Fig. 5D). They range in habitat from cobbles shores of small, cold, clear rivers (Fig. 6D) to mixed shores of large rivers (Fig. 6A), and sand shores of desert rivers (Fig. 6C) to warm, exposed salt flats (Fig. 6B). In the field, beetles in this group give the appearance of rather different groups of carabids. In my first field encounter with live *Bembidion lonae*, I mistook them for tachyines of the genus *Elaphropus* Motschulsky; *Bembidion germainianum* is reminiscent of the Nearctic *Bembidion perspicuum* Casey, a member of the *Ocydromus* Series of *Bembidion*; *Bembidion eburneonigrum* looks very much like a small member of the subgenus *Notaphus* as it scurries on the sandy shores of rivers.

There are many examples of clades throughout the tree of life that contain species of diverse forms living in diverse habitats. Why then is *Nothonepha* unexpected? The current classification, to the extent that it might be a predictor of relationships, would suggest that these taxa are not related. *Bembidion lonae* is the only described species of those sampled that was placed in *Nothonepha*; the very similar but undescribed *Bembidion* sp. nr. *lonae* would have been placed there as well. The other described species (*Bembidion germainianum*, *Bembidion engelhardti*, *Bembidion tucumanum*, *Bembidion eburneonigrum*, and *Bembidion tucumanum*) had all been classified in subgenus *Antiperyphus*, along with *Bembidium philippii*. When I first discovered *Bembidion tetrapholeon*, I thought it represented a separate lineage requiring a new subgeneric name, as it is very different in form from any other South American *Bembidion*. This apparently added a third element to the diverse group. However, the current classification, constructed with limited data and without phylogenetic analyses, may not be the best predictor of phylogenetic relationships.

Nonetheless, *Nothonepha* is a morphologically heterogeneous group (Fig. 6). I have been studying *Bembidion* systematics for over three decades, and whatever predictive map my brain has developed from all the data accumulated over the years contained no hint that the species shown in Fig. 6 formed a clade.

However, it may not be the diversity of form within *Nothonepha* that is unusual, but rather the diversity *given* the lack of intermediate forms and small size of the group. Other clades in the South American fauna are also diverse in form and size (e.g., *Ecuadion*, Fig. 3), but they have many more species, some of which are intermediate between the more distinct forms. The lack of intermediate forms in *Nothonepha* may be a result of low speciation rates with high rates of morphological evolution, or it might be a result of extinction of intermediate forms; it is unlikely to be a lack of sampling, as enough collecting has been done in South America to suggest that there are not a large number of undescribed species of *Nothonepha*. A full investigation of patterns of morphological and molecular rates, times of divergence, and speciation and extinction rates relative to other clades is beyond the scope of this paper, but would be a worthwhile topic for future studies.

**Function of mesepisternal pits.** Exoskeletal invaginations are widespread in beetles (Grebennikov and Leschen 2010). These cavities occur on many different body parts, including in the lateral regions of the mesothorax (e.g., in the ptiliid tribe Discheramocephalini (Grebennikov 2008; 2009)); their function probably varies from group to group. Many have been thought to be mycangia for storing fungal spores, but this is well documented in only two of the many independent origins of such cavities (Grebennikov and Leschen 2010). In a few groups of polyphagan beetles (e.g., the Staphylinoidea subfamily Scydmaeninae, the Cucujoid families Cyclaxyridae and Nitidulidae, the Tenebrionoidea family Zopheridae) there are species with pits containing a waxy substance (Grebennikov and Leschen 2010). Wax has been proposed to act as a defensive shield (Lawrence and Hlavac 1979), or as a medium for retaining fungal spores (Grebennikov and Leschen 2010).

The functions of mesepisternal pits in the two carabid groups in which they were previously described is not known, but functions have been hypothesized. Erwin (1970) proposes that pits of *Elaphropus (Tachylopha)* are insertion points for ant mandibles, allowing ants to carry the adults around. Bousquet (1996) calls the structures in *Oodinus* “apodemal pits”, which implies a function as internal attachment points for muscles.

The similarities between the wax-filled mesepisternal pits observed in the bembidiine *Bembidion (Nothonepha)*﻿, the tachyines *Elaphropus (Tachylopha)* and *Elaphropus (Sphaerotachys)*﻿, and the oodine *Oodinus* are striking (e.g., Figs 9A and 12A in this paper, and Fig. 145 in Bousquet (1996)), enough so that a common function might be hypothesized. Although the shape and nature of the deepest pits and largest intrusions differs between the groups (e.g., there are no *Nothonepha* known with the merged intrusions of some *Tachylopha*), the less extreme forms are indistinguishable externally and internally. These features are surely convergent, as the clades are not closely related. *Bembidion* and *Elaphropus* are both members of the subfamily Trechinae, but they are each deeply nested within independent clades (Maddison and Ober 2011); *Oodinus* is a member of the tribe Oodini (Bousquet 1996; Spence 1982), which is nested within several clades in the superfamily Harpalinae, itself a well-supported clade (Maddison et al. 1999; Ober and Heider 2010). It may be that these pits serve different functions in these clades, but consistency of structure suggests they may be serving the beetles in similar ways in the three different groups.

Correlates in way of life might provide some hints about function. Members of the three carabid groups are all presumably generalist predators, as is typical in Carabidae (Thiele 1977). They are also all terrestrial, but associated with shorelines. *Bembidion (Nothonepha)* species occur at edges of bodies of water in southern South America. *Elaphropus (Tachylopha)* occurs in similar habitats. I have seen *Elaphropus (Tachylopha) spenceri* (Sloane) (identified using Baehr (1988)) from multiple localities in Queensland, Australia, labeled as being found at “water’s edge” along creek shores. Bruneau de Miré (1966) reports numerous species along rivers, and states that they can be abundant in swamps and moist forest humus. *Oodinus* occurs at the edges of marshes and swamps (Bousquet 1996; Spence 1982). However, many other carabid groups lacking these pits are also found in these habitats, including many other *Bembidion*, *Elaphropus*, and Oodini.

It appears unlikely that mesepisternal pits are used as ant handles in *Bembidion (Nothonepha)* and *Oodinus*, and probably not in *Elaphropus (Tachylopha)*. Erwin’s (1970) hypothesis was based in part on the unusual elytral structure of *Tachylopha*, which is narrow above the mesepisternum and which possesses a notch into which the base of ant mandibles could fit. Although most *Nothonepha* have narrow-enough pronota to allow curved, sickle-shaped mandibles of a large ant access to the pits, that seems much less likely for *Oodinus*, which are wide-bodied, oval carabids. *Oodinus* have a distinct ledge along the lateral edge of their bodies; to fit a mandible tip into the pit underneath this ledge an ant would need exceptionally curved mandibles. In general, ground-nesting ants are relatively rare in wet, near-shore, seasonally inundated habitats, and one would not expect large ants (ground-nesting or arboreal) with sufficiently curved mandibles in these near-shore environments (Philip S. Ward, pers. comm.). I have observed ants only rarely in the near-water habitats of subgenus *Nothonepha*, and not at all in the case of the four localities at which I have collected *Bembidion tetrapholeon* or the three localities at which I have found *Bembidion germainianum*. Furthermore, there is no evidence for any association between ants and these three carabid groups.

There is also evidence against the mesepisternal pits functioning as apodemes. As noted above, *Nothonepha* and *Tachylopha* do not have muscles attached to the internal walls of the mesepisternal pits. Spence (1982) reports that there are no conspicuous muscles that attach to the internal walls of the pits in *Oodinus*.

It is possible that the function is as a reservoir for the wax, although where the wax is produced is not evident. Spence (1982) states that the pit walls are perforated by numerous channels in *Oodinus*; these might be ducts for glandular secretions. I have not detected any large glands internally near the intrusions, although there is a thin layer of tissue in places on the inner surface of the structures. It is also possible that the wax is not produced near the intrusion; it might be produced elsewhere on the beetle’s body, and in fluid form flow into the pits. (As noted above, the wax is not evident in many ethyl acetate killed specimens, but rather in ethanol killed specimens, suggesting that the substance may precipitate in ethanol, but exist as a liquid otherwise.) More detailed histological work is needed to explore possible glandular sources.

Whatever the source of the wax, its function (if it has one) is unclear. It seems unlikely that it would be for retaining fungal spores (Grebennikov and Leschen 2010), as there is nothing known about these generalist predators that would suggest a benefit to the beetle to retain fungal spores. Wax as a defensive coating is plausible (Lawrence and Hlavac 1979), but more studies are needed to explore this and other possibilities.

**Function of abdominal pits.** In contrast to the mesepisternal pits, the abdominal pits in *Bembidion (Nothonepha)* evidently serve (in part) as apodemes, that is, as attachment points for muscles. However, the function of those muscles is unclear; in general the nature and function of muscles at the junction of the metathorax and abdomen in beetles is poorly known (Rolf Beutel, pers. comm. 2014). Even if the abdominal pits serve as apodemes, they may serve additional functions as well, perhaps related to the presence of wax that appears similar to that found in the mesepisternal pits.

## Taxonomic treatment

Three clades of *Bembidion* comprise the South American fauna: subgenus *Notaphus* Dejean (32 species (Jeannel 1962; Toledano 2002; Toledano 2008)), subgenus *Nothocys* Jeannel (13 species (Toledano 2002; Toledano 2008)), and the *Antiperyphanes* Complex. Along with a number of lineages outside of South America, these all belong to one subclade of *Bembidion*, the *Bembidion* Series (Maddison 2012). Subgenus *Notaphus* is widespread and abundant throughout the New World, with over 50 species known from North America; in addition, seven species occur in the Old World (Lobl and Smetana 2003). *Nothocys* is restricted to southern South America, occurring in Chile, Argentina, Peru, and Bolivia (Toledano 2002; Toledano 2008). The largest of these clades is the *Antiperyphanes* Complex, with about 95 described species (Jeannel 1962; Maddison 2012; Toledano 2002; Toledano 2008), and many undescribed.

### Antiperyphanes Complex

Taxon classificationAnimaliaColeopteraCarabidae

#### Remarks.

Members of the *Antiperyphanes* Complex are diverse in form (Figs 1–3, 5). There are no recognized derived morphological characteristics of the group, although the clade is moderately well supported by the concatenated DNA sequence data (Fig. 8, Table 3). Within the South American fauna, most species can be recognized by the lack of an N sclerite in the internal sac of the male genitalia (Toledano 2008). However, some members of the complex, including some species in subgenus *Nothonepha*, have a small sclerite that could be homologous to the N sclerite (Toledano 2008).

The *Antiperyphanes* Complex, as here classified, consists of at least five subgenera: *Antiperyphanes*, *Chilioperyphus*, *Antiperyphus*, *Notholopha*, *Ecuadion*, and *Nothonepha*. Each of these subgenera is briefly discussed below, with notes about their composition. Two other poorly known subgenera, *Pseudotrepanes* Jeannel and *Notoperyphus* Bonniard de Saludo, are likely members of this complex, but specimens will need to be examined to confirm their membership.

### Subgenus Antiperyphanes

Taxon classificationAnimaliaColeopteraCarabidae

Jeannel, 1962

Antiperyphanes Jeannel, 1962; type species *Bembidium spinolai* Solier, by original designation.
Plocamoperyphus Jeannel, 1962; type species *Bembidium mandibulare* Solier, by original designation. New synonymy.

#### Remarks.

This group contains at least 19 described species (Toledano 2002; Toledano 2008), and is characterized by males having an aedeagus lacking a brush sclerite, and with a very long flagellum (Maddison et al. 2013; Toledano 2008).

Included here are some species previously placed in subgenus *Antiperyphus* by Jeannel (1962): *Bembidion hirtipes* (Jeannel), *Bembidion ringueleti* (Jeannel), *Bembidion rufoplagiatum* Germain, *Bembidion uniforme* Csiki, and *Bembidion parvum* (Jeannel). *Bembidium mandibulare* Solier belongs to *Antiperyphanes* as well, and is nested well within it (Fig. 8); thus, subgenus *Plocamoperyphus* is a synonym of *Antiperyphanes*. There are no morphological characteristics of *Bembidium mandibulare* that would suggest it is not a member of *Antiperyphanes*; it shares all apomorphies of the group. As first reviser, I choose *Antiperyphanes* as the valid name of the group.

*Antiperyphanes* has two distinct clades, each very well supported: the *Bembidion caoduroi* group (clade **j** in Fig. 8; Fig. 1D), consisting (among the sampled species) of three large species from the northern Andes (Fig. 1D); (2) the remaining *Antiperypanes* (clade **k** in Fig. 8; Figs 1A–C, E). Each is supported by MLB=100 and PB=100 in the multi-gene analyses, and individually by support from five to seven genes (Table 3). As a whole, however, the monophyly of *Antiperyphanes* is only weakly supported by the combined analysis and analyses of four genes (Table 3).

Members of this subgenus are found at the edges of bodies of water. For example, *Bembidion rufoplagiatum* and *Bembidion zanettii* are common on gravel and cobble river shores, *Bembidion ringueleti* is found on gravel and sand shores of smaller creeks, and *Bembidium mandibulare* on the sand beaches of the Pacific Ocean in Chile and sand beaches of rivers in Argentina.

### Subgenus Chilioperyphus

Taxon classificationAnimaliaColeopteraCarabidae

Jeannel, 1962

Chilioperyphus Jeannel, 1962; type species *Bembidium orregoi* Germain, by original designation.

#### Remarks.

This subgenus contains two described species (Jeannel 1962; Maddison et al. 2013) (Fig. 1F) and at least three undescribed species. Males are characterized by having a brush sclerite, and by having an extremely elongate flagellum, so long that it can only fit within the median lobe through folding (Maddison et al. 2013). Members of this subgenus occur on steep sand or clay banks of rivers and creeks.

### Subgenus Antiperyphus

Taxon classificationAnimaliaColeopteraCarabidae

Jeannel, 1962

Antiperyphus Jeannel, 1962; type species *Bembidium philippii* Germain, by original designation.

#### Remarks.

As noted by Toledano (2008), Jeannel’s concept of *Antiperyphus* was polyphyletic, with at least *Bembidion hirtipes*, *Bembidion ringueleti*, *Bembidion rufoplagiatum*, and *Bembidion uniforme* Csiki belonging within *Antiperyphanes*. This is confirmed in part by my results (Fig. 8, Table 3). In addition, *Bembidion engelhardti*, *Bembidion eburneonigrum*, *Bembidion tucumanum*, and *Bembidion germainianum* are members of *Nothonepha*, not *Antiperyphus*. Of the species included in the subgenus by Jeannel (1962), this leaves only the type species, *Bembidium philippii* (Fig. 2A).

In addition, *Bembidion peterseni* Jensen-Haarup (1910), from Mendoza, Argentina, can tentatively be placed here. I have examined a male syntype (in ZMUC), and it is similar in appearance to *Bembidium philippii*, although with much deeper and longer elytral striae. It is not a member of *Nothonepha* (as it lacks mesepisternal pits), nor is it a member of *Antiperyphanes* (it has a brush sclerite, and does not have the long flagellum characteristic of *Antiperyphanes*). The internal sac of the male genitalia, although difficult to see because of the nature of the preparation, appears very similar to that of *Bembidium philippii*.

B. *philippii* is common on sand shores of rivers in the provinces of Neuquén and Chubut in Argentina; it also occurs in Chile.

### Subgenus Notholopha

Taxon classificationAnimaliaColeopteraCarabidae

Jeannel, 1962

Notholopha Jeannel, 1962; type species *Bembidium punctigerum* Solier, by original designation.
Pacmophena Jeannel, 1962; type species *Bembidium scitulum* Erichson, by original designation.

#### Remarks.

*Notholopha* consists of 11 described species (Toledano 2002; Toledano 2008), and several undescribed (two of which are sequenced here). These are small beetles with large, protruding eyes (Fig. 2B, C), with small flagella in the internal sac of the male genitalia, and with brush sclerites. They have the general appearance when running of a member of the Holarctic subgenus *Bembidion*. Some frequent habitats similar to those of subgenus *Bembidion*, including dry habitats far from water (*Bembidion stricticolle*), or upper banks of creeks (e.g., *Bembidion* sp. “Nahuelbuta”). Others occur at high elevation near small rivulets in open, alpine areas (e.g., *Bembidion rugosellum* and *Bembidion melanopodum*), or at the edges of snowfields (*Bembidion sexfoveolatum*).

Jeannel considered *Pacmophena* and *Notholopha* to be two subgenera within the genus *Notholopha*. As the characters that Jeannel used to distinguish the two are minor characters such as surface texture and antennal length, and as it appears that *Pacmophena* is paraphyletic with respect to the *Notholopha* (*s. str.*) (Fig. 7), I consider them synonymous, with *Notholopha* as the valid name.

### Subgenus Ecuadion

Taxon classificationAnimaliaColeopteraCarabidae

Moret & Toledano, 2002

Ecuadion Moret & Toledano, 2002; type species *Bembidion fulvocinctum* Bates, by original designation.

#### Remarks.

*Ecuadion* is the largest subgenus in the *Antiperyphanes* Complex, with over 50 described species (Maddison and Toledano 2012; Moret and Toledano 2002; Toledano 2008; Vigna Taglianti and Toledano 2008) and likely many undescribed. It occurs from Costa Rica south to the mountains near Mendoza, Argentina. There are no known exoskeletal synapomorphies of the group, but it is well-supported by the molecular data, with bootstrap support in six of the seven genes examined (Table 3).

*Ecuadion* falls into two distinct clades among the sampled species: (1) the *Bembidion chimborazonum* group (clade **m** in Fig. 8), consisting mostly of larger, long-legged species (Figs 3A, B); (2) remaining *Ecuadion* (clade **n** in Fig. 8), consisting of mostly smaller species with shorter appendages (Figs 3C–F). The *Bembidion chimborazonum* group is supported by all genes examined (Table 3); support for the complementary clade is not quite as strong, with the clearest evidence coming from ribosomal genes (Table 3).

Unlike most *Bembidion*, this subgenus has radiated in habitats away from water. Some occur in leaf litter in cloud forests (e.g., *Bembidion andersoni*, *Bembidion georgeballi*, *Bembidion onorei*, *Bembidion* sp. “Papallacta”; Fig. 4D), in habitats that would typically be occupied by the genus *Trechus* Clairville in North America. A number of species are found in open, high-elevation grasslands (e.g., *Bembidion chimborazonum*, *Bembidion guamani*, *Bembidion humboldti*; Fig. 4C). Some species occur on the upper banks of creek shores (e.g., *Bembidion sanctaemarthae*, *Bembidion ricei*); others are found on clay or silt cliffs (e.g., *Bembidion agonoides*, *Bembidion walterrossii*).

### Subgenus Nothonepha

Taxon classificationAnimaliaColeopteraCarabidae

Jeannel, 1962

Nothonepha Jeannel, 1962; type species *Bembidium baptisatum* Csiki (=*Bembidion lonae* Jensen-Haarup), by original designation.

#### Remarks.

As here defined, the subgenus *Nothonepha* includes all species of the *Antiperyphanes* Complex possessing mesepisternal pits. Seven described species belong to *Nothonepha*:

*Bembidion lonae* Jensen-Haarup, 1910 (Fig. 5D)

*Bembidion pallideguttula* Jensen-Haarup, 1910

*Bembidion eburneonigrum* Germain, 1906 (Fig. 5E)

*Bembidion engelhardti* Jensen-Haarup, 1910

*Bembidion engelhardti engelhardti* Jensen-Haarup, 1910 (Fig. 5G)

*Bembidion engelhardti rayoda* Toledano, 2008

*Bembidion tucumanum* (Jeannel, 1962) (Fig. 5F)

*Bembidion germainianum* Toledano, 2002 (Fig. 5C)

*Bembidion tetrapholeon* Maddison, sp. n. (Figs 5A, B)

Four of these species (*Bembidion germainianum*, *Bembidion tucumanum*, *Bembidion engelhardti*, and *Bembidion eburneonigrum*) were formerly placed in subgenus *Antiperyphus*. In addition, there are at least three undescribed species (including *Bembidion* sp. nr. *lonae*, sequenced here). The species figured by Toledano (2008) as *Bembidion germainianum* is an undescribed species related to *Bembidion germainianum*. A revision of the subgenus is in preparation (Roig-Juñent and Maddison).

### Bembidion (Nothonepha) tetrapholeon sp. n.

Taxon classificationAnimaliaColeopteraCarabidae

http://zoobank.org/90188564-6B1E-4F0E-B411-5AD99047F716

**Figure 15.**
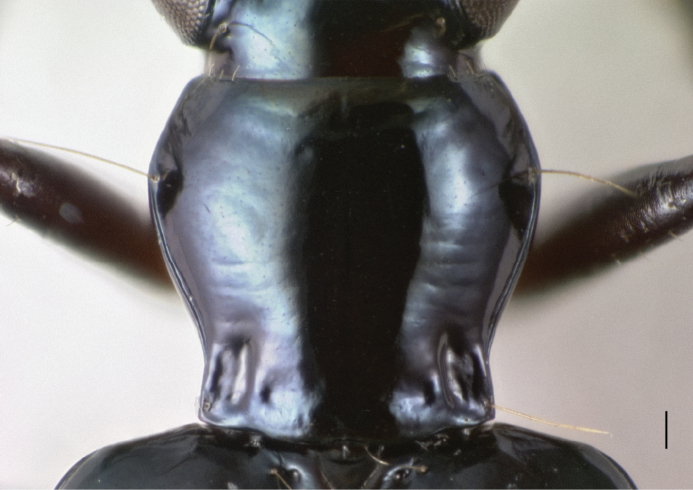
Pronotum of *Bembidion tetrapholeon*, DRM voucher V100781. Scale bar 0.1 mm.

**Figure 16.**
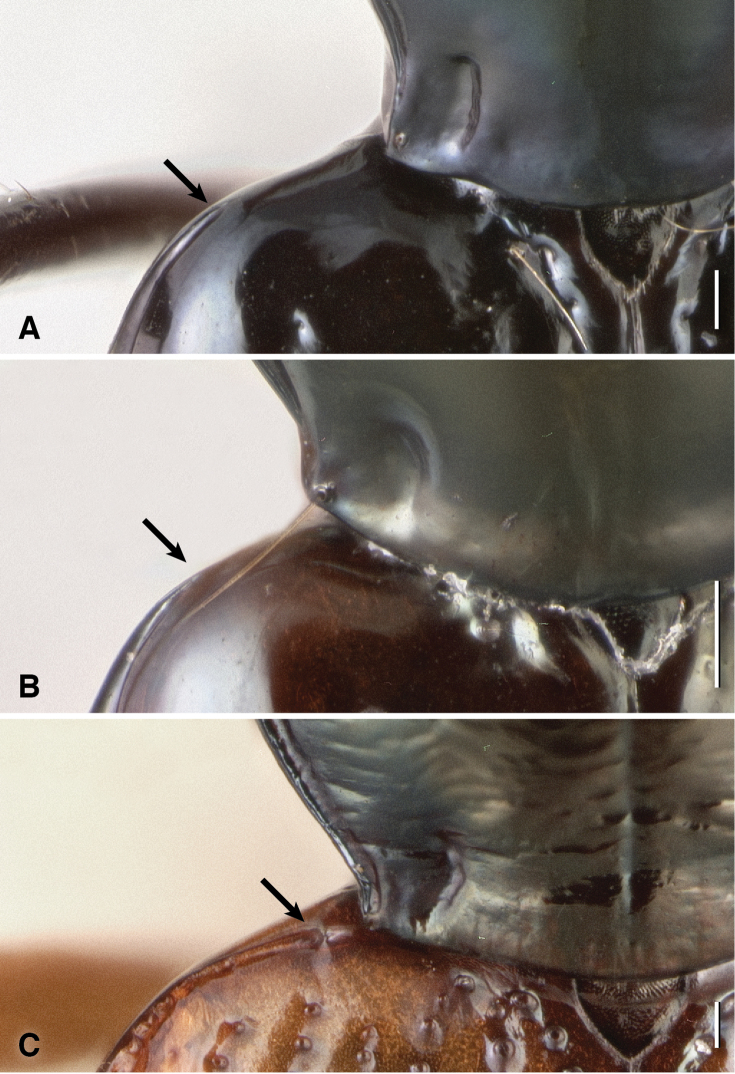
Humeral region of left elytron and posterior corner of pronotum of three *Bembidion (Nothonepha)* species. **A** *Bembidion tetrapholeon*, DRM voucher V100781 **B** *Bembidion lonae*, DRM voucher V100786 **C** *Bembidion germainianum*, DRM voucher V100782. Arrows show the anterior end of the lateral elytral groove and bead. Scale bar 0.1 mm.

**Figure 17.**
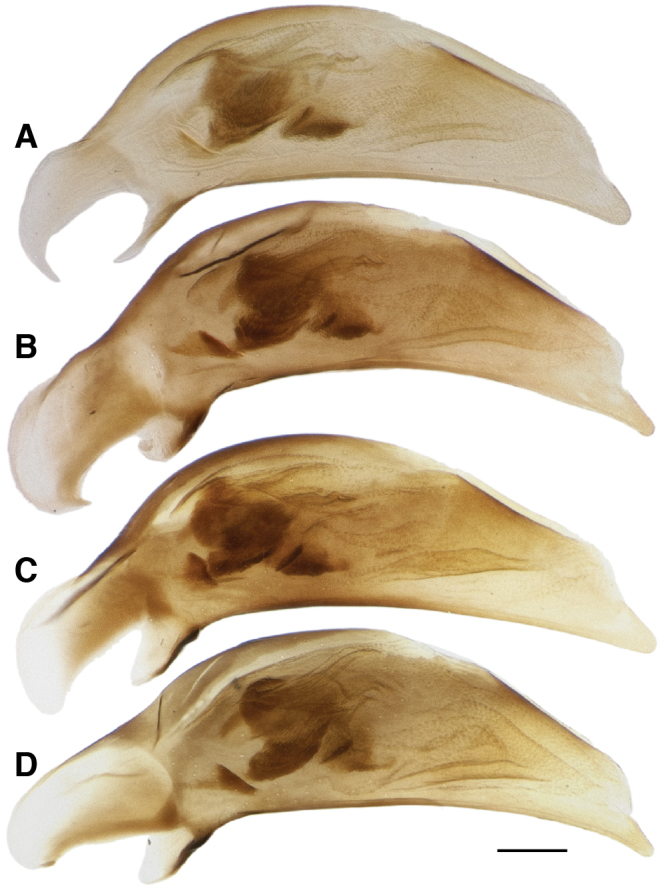
Male aedeagus of *Bembidion tetrapholeon*. **A** Black form, Chile: Region X, Rio Pullinque at Puente Huanehue, 8 km NE Panguipulli, DRM voucher DNA1752 **B** Black form, Chile: Reg. X, Chiloé: Rio Puntra at route 5, DRM voucher DNA2236 **C** Orange-spotted form, Argentina: Neuquén: Rio Pichi Traful nr Lago Traful, DRM voucher DNA2564 **D** Orange-spotted form, Argentina: Neuquén: Rio Pichi Traful nr Lago Traful, DRM voucher DNA2555. Scale bar 0.1 mm.

**Figure 18.**
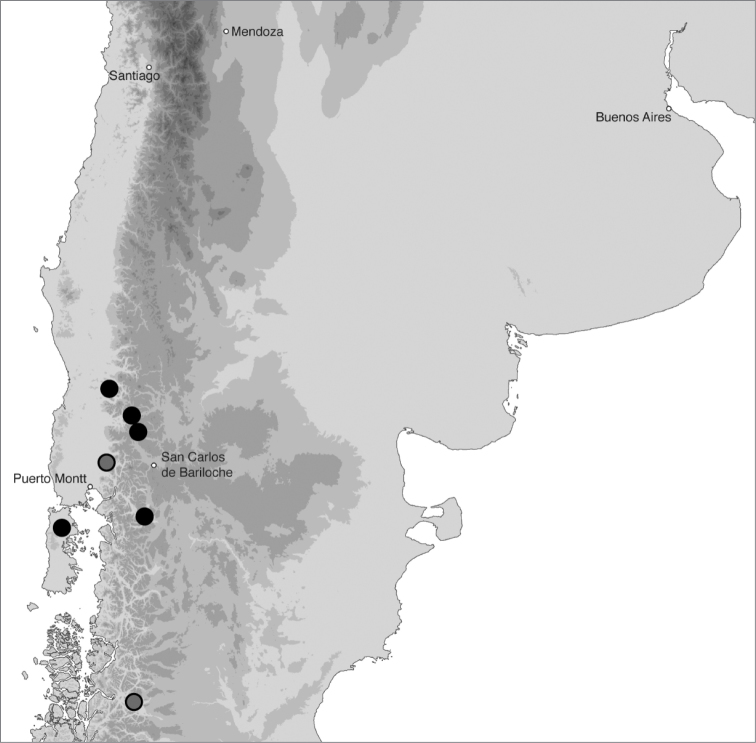
Geographic distribution of *Bembidion tetrapholeon*. Circles filled in black are based upon specimens I have examined and that have been sequenced; circles filled in gray are based upon specimens identified by Luca Toledano. Five cities are included as landmarks.

Figs 5A, B , 9A , 15 , 16 , 17A , 18

#### Holotype

male (IADIZA), with 3 labels: “Argentina: Neuquén: Arroyo / Queñi at Lago Queñi, 830m, / 40.1575°S, 71.721°W, / 10-11.ii.2007. DRM 07.035. / D.R. Maddison, S.A.Roig”, “David R. Maddison / DNA2356 / DNA Voucher” [printed on pale green paper], and “HOLOTYPE / Bembidion / tetrapholeon / David R. Maddison” [printed on red paper]. Genitalia in glycerine in small plastic vial beneath specimen; extracted DNA stored separately. GenBank accession numbers for DNA sequences of the holotype are KJ653049 (28S), KJ653145 (COI), KJ653112 (CAD), KJ653181 (Topo), KJ653215 (*wg*), and KJ653082 (ArgK).

#### Paratypes.

Total of 244, in IADIZA, MACN, MNNC, OSAC, MNHN, BMNH, EMEC, CTVR, and CMNH, from “Argentina: Neuquén: Arroyo / Queñi at Lago Queñi, 830m, / 40.1575°S, 71.721°W, / 10–11.ii.2007. DRM 07.035. / D.R. Maddison, S.A.Roig” [135 exx.], Argentina: Neuquén: Rio Pichi / Traful nr Lago Traful, 810m, / 40.4867°S, 71.5958°W, / 12.ii.2007. DRM 07.039. / D.R. Maddison, S.A.Roig” [95 exx], “Argentina: Chubut: Rio / Azul at Lago Puelo, 200m / 42.0929°S, 71.6244°W / 13.ii.2007. DRM 07.044. / D.R. Maddison” [12 exx], “Argentina: Chubut: Rio / Azul at Lago Puelo, 200m / 42.0929°S, 71.6244°W, / 13.ii.2007. DRM 07.045. / S.A.Roig, D.R. Maddison” [1 exx].

#### Additional material examined.

CHILE: Reg. X, Chiloé: Rio Puntra at rt 5, 55m, 42.1661°S, 73.7256°W, 19.i.2006. DRM 06.075. D.R. Maddison [5 exx, OSAC, MNNC]. CHILE: Region X, Rio Pullinque at Puente Huanehue, 8 km NE Panguipulli. 16 Jan 2002, 39.6162°S, 72.2286°W, 1590 ft. W. D. Shepard [2 exx, OSAC].

#### Additional identified material.

The following specimens have been examined by Luca Toledano and confirmed to belong to this species (based upon photographs we have shared). CHILE: Reg. X, Los Lagos, P.N. Vicente Péres Rosales, Petrohué, Lago Todos los Santos, 190m, mouth Rio El Caulle, 41.0924°S, 72.3950°W. 5.i.2014. L. Toledano, R. Olivieri, J.P. Morales. [1 ex, CTVR]; CHILE: Region XI, Parque Nat. Rio Simpson, H. Franz [4 exx, NHMW]; CHILE: Reg. X, I. Chiloé, R. Punta, 31.i.1986. M. Spies. [1 ex, USNM].

#### Type locality.

Argentina: Neuquén: Arroyo Queñi at Lago Queñi, 830m, 40.1575°S, 71.7210°W. The habitat at the type locality is a cobble, gravel, and coarse sand river shore (Fig. 6D); the river is cold and has crystal-clear water. In the same habitat members of the genus *Bembidarenas* Erwin are abundant, as is *Bembidion (Antiperyphanes) rufoplagiatum*.

#### Derivation of specific epithet.

From the Greek “*tetra*”, meaning “four”, and “*pholeon*”, meaning “pit”, referring to the four prominent pits visible on the underside of adults.

#### Diagnosis.

A large, sleek, shiny *Bembidion*, with an unusual body form (Figs 5A, B) of narrow forebody and large elytra. With its shape, color, and smoothness it is one of the most distinctive *Bembidion* species in South America, and no other known species is likely to be confused with it; it is more reminiscent of some species in New Zealand, e.g., *Bembidion (Zeplataphus) dehiscens* Broun (Lindroth 1976).

Length (4.7–5.7 mm, with most specimens above 5.0 mm). Color piceous (Fig. 5A), with legs and antennae in some specimens slightly paler, and with a few specimens having a large orange spot just in front of the elytral apices (Fig. 5B).

Head with shallow and parallel frontal furrows.

Pronotum narrow, cordate, with hind angles flaring outward (Fig. 15). Very smooth, without punctures, and with a linear basolateral foveae; without distinct carina at hind angle. Lateral bead of pronotum not complete, not reaching front angle of prothorax and only in some specimens reaching the hind angle. One midlateral and one basolateral seta on each side.

Each elytron with two discal setae (ed3 and ed5); ed3 in third stria. Elytral striae with prominent punctures in their basal half, but with striae 2–7 absent in about the hind 40% or more of the elytra, such that the posterior discal seta, ed5, is in a region without striae. Striae 7 absent in many specimens. In many specimens the striae are effaced anteriorly, especially striae 2 and 3. Lateral bead of elytron effaced anteriorly, not extended onto shoulder (Fig. 16A), similar to that of *Bembidion (Nothonepha) lonae* (Fig. 16B), but unlike most other *Bembidion* (e.g., *Bembidion (Nothonepha) germainianum*, Fig. 16C).

Mesothorax with prominent pits in the mesepisternum (Fig. 9A), which appear internally as large intrusions that touch in the middle (Figs 10A, C, E). Smaller pits are present ventrally at the junction of abdominal segments II and III (Fig. 13A), which are evident internally as knob-like intrusions (Fig. 14A).

Hind wings full.

Microsculpture absent from entire dorsal surface of the body except for the cervical region of the head, labrum, and faintly on the clypeus; the beetles are thus brilliantly shiny. Microsculpture absent from most of the ventral surface as well, with the most notable microsculpture being on the undersurface of the head.

Aedeagus with nearly straight ventral margin, tip of variable width (Fig. 17). Prominent brush sclerite, and with flagellum not clearly evident from the left side. There is no evident correlation between aedeagal structure and presence or absence of orange spots on the elytra (compare Figs 17A, B to Figs 17C, D).

#### Morphological variation.

The most noted variation is in color of the elytra. Of the 257 specimens examined (including the six specimens identified by Luca Toledano), 245 have uniformly piceous elytra (Fig. 5A); the remaining 12 have a large, diffuse orange spot occupying most of the posterior third of the elytra (Fig. 5B). Ten of these orange-spotted specimens are from the three localities in Argentina, with at least one orange-spotted specimen from each locality, and two of the orange-spotted specimens are from Chile. In most of orange-spotted specimens, the posterior discal seta (ed5) is in the orange region, but immediately around the seta is a small dark spot. No other aspect of morphological or molecular variation was observed to be correlated with presence or absence of the orange spot.

#### DNA sequence variation.

As noted above under Results, there was minor variation present in COI and the *wingless* gene, and no variation in the other genes studied.

#### Habitat and seasonality.

At all four localities where habitat data were recorded, *Bembidion tetrapholeon* specimens were found on cobble, gravel, and coarse sand shores of clear, fast-flowing rivers (Fig. 6D), from 55 m elevation (Rio Puntra, Isla Grande de Chiloé, Chile) to 830 m elevation (Arroyo Queñi, Neuquén, Argentina). These shorelines lack vascular plants. The beetles occur close to the water, most within 1 m. Specimens have been found in January and February.

#### Geographic distribution.

In southern Argentina and Chile (Fig. 18). In Argentina this species has been found in Neuquén and Chubut, and in Chile from Regions X and XI.

#### Relationship to other *Bembidion*.

*Bembidion tetrapholeon* is a member of subgenus *Nothonepha*, as strongly supported by DNA sequences (Table 3) and the presence of shared, derived mesepisternal pits. *Bembidion tetrapholeon* appears to be the sister of remaining *Bembidion (Nothonepha)* (Figs 7, 8). Four genes support this placement (CAD, *wg*, ArgK, and 18S; Table 3), although the support is weak or moderate in single-gene analyses.

## Concluding remark

“*when the same organ appears in several members of the same class, especially if in members having very different habits of life, we may attribute its presence to inheritance from a common ancestor.*” (Darwin 1859)

In groups such as beetles, in which a preponderance of lineages split without later reticulation, the evolutionary tree at the core of life’s history yields hierarchical patterns in the distributions of characteristics. Any particular lineage in the tree, if separated long enough or with a high enough rate of evolution, will leave in the bodies of its descendants marks of its existence. The echoes from that deep historical well can reverberate down through later lineages in the form of signals scattered throughout the genomes. The repeated patterns of these branch markers both in the DNA and on the bodies of organisms are among the most compelling signs of the existence of the tree of life, and provide to us evidence about its shape. On occasion the clades thereby revealed are so unexpected that it is only with multiple independent markers, all showing the same pattern, that we can confidently accept the existence of the clade. *Nothonepha* is such a clade.

Many of us who study the diversity of life, and see the hierarchical patterns of organismal traits, are steeped in evidence of the existence of a genetic tree of life, so much so that we perhaps take it for granted (I often do). I think about the evidence about the tree’s shape, but much less so evidence about its existence. In encountering the first evidence of *Nothonepha*, my belief in the tree-like structure of beetle genetic history was challenged, as the data made little sense in that light. As newly sequenced genes added to the evidence, my acceptance of the clade increased. The struggle was fully resolved when the mesothoracic pits came to light; this harmonizing of the morphological data with the molecular not only instilled a firm belief in this clade, but also a simple confirmation of the tree itself.

## Supplementary Material

XML Treatment for Antiperyphanes

XML Treatment for Antiperyphanes

XML Treatment for Chilioperyphus

XML Treatment for Antiperyphus

XML Treatment for Notholopha

XML Treatment for Ecuadion

XML Treatment for Nothonepha

XML Treatment for Bembidion (Nothonepha) tetrapholeon

## Appendix 1

**Locality data for newly sequenced specimens**

**Table:** Locality data for *Bembidion* specimens newly sequenced for this study. Under “#” the D.R. Maddison DNA voucher number is listed.

	#	Locality
*Bembidion agonoides*	2675	ECUADOR: Napo: Vinillos, 4.1 km S Cosanga, 2090m, 0.6024°S, 77.8509°W
*Bembidion andersoni*	2651	ECUADOR: Pichincha: Reserva Yanacocha, start Andean Snipe Trail, 3540m, 0.1152°S, 78.5837°W
*Bembidion chimborazonum*	2659	ECUADOR: Pichincha: Paso de la Virgen, 4070m, 0.3331°S, 78.2025°W
*Bembidion cotopaxi*	2658	ECUADOR: Pichincha: Quebrada Lozada, on road to Res. Yanacocha, 3460m, 0.1105°S, 78.5642°W
*Bembidion eburneonigrum*	2204	CHILE: Reg. IX: Rio Allipén at route 119, 132m, 39.0164°S, 72.5045°W
*Bembidion engelhardti*	2334	ARGENTINA: Neuquén: Rio Salado at route 40, 725m, 38.2143°S, 70.0931°W
*Bembidion georgeballi*	2661	ECUADOR: Pichincha: Quebrada Lozada, on road to Res. Yanacocha, 3455m, 0.1105°S, 78.5642°W
*Bembidion germainianum*	2336	ARGENTINA: Neuquén: Rio Salado at route 40, 725m, 38.2143°S, 70.0931°W
*Bembidion guamani*	2660	ECUADOR: Pichincha: Paso de la Virgen, 4070m, 0.3331°S, 78.2025°W
*Bembidion humboldti*	2673	ECUADOR: Pichincha: Paso de la Virgen, 4070m, 0.3331°S, 78.2025°W
*Bembidion jimburae*	2674	ECUADOR: Napo: Rio Guango, 2730m, 0.3758°S, 78.0748°W, 26.x.2010
*Bembidion neodelamarei*	2342	ARGENTINA: Mendoza: Uspallata, 1880m, 32.5908°S, 69.3513°W, 25.ii.2007
*Bembidion onorei*	2678	ECUADOR: Pichincha: Quebrada Lozada, on road to Res. Yanacocha, 3455m, 0.1105°S, 78.5642°W
*Bembidion paulinae paulinae*	2783	ECUADOR: Pichincha: Quebrada Lozada, on road to Res. Yanacocha, 3455m, 0.1105°S, 78.5642°W
*Bembidium philippii*	2327	ARGENTINA: Neuquén: Rio Collón Curá ca 13 km S La Rinconada, 625m, 40.1015°S, 70.7545°W
*Bembidion ricei*	2653	ECUADOR: Napo: Rio Chalpi Grande, 2800m, 0.3645°S, 78.0852°W
*Bembidion sanctaemarthae*	2652	ECUADOR: Napo: Rio Chalpi Grande, 2780m, 0.3657°S, 78.0848°W
*Bembidion* sp. nr. *caoduroi*	2677	ECUADOR: Napo: Rio Cosanga at mouth of Rio Angenaro, 2185m, 0.6407°S, 77.9083°W
*Bembidion stricticolle*	2240	CHILE: Reg. IX: ca. 28 km E Melipeuco, 1262m, 38.83°S, 71.4038°W
*Bembidion tetrapholeon*	1752	CHILE: Region X, Rio Pullinque at Puente Huanehue, 8 km NE Panguipulli. 39°36’58’’S, 72°13’43’’W, 1590 ft.
*Bembidion tetrapholeon*	2236	CHILE: Reg. X, Chiloé: Rio Puntra at rt 5, 55m, 42.1661°S, 73.7256°W
*Bembidion tetrapholeon*	2356	ARGENTINA: Neuquén: Arroyo Queñi at Lago Queñi, 830m, 40.1575°S, 71.7210°W
*Bembidion tetrapholeon*	2357	ARGENTINA: Neuquén: Arroyo Queñi at Lago Queñi, 830m, 40.1575°S, 71.7210°W
*Bembidion tetrapholeon*	2555	ARGENTINA: Neuquén: Rip Pichi Traful nr Lago Traful, 810m, 40.4867°S, 71.5958°W
*Bembidion tetrapholeon*	2562	ARGENTINA: Neuquén: Arroyo Queñi at Lago Queñi, 830m, 40.1575°S, 71.7210°W
*Bembidion tetrapholeon*	2563	ARGENTINA: Chubut: Rio Azul at Lago Puelo, 200m, 42.0929°S, 71.6244°W
*Bembidion tetrapholeon*	2564	ARGENTINA: Neuquén: Rip Pichi Traful nr Lago Traful, 810m, 40.4867°S, 71.5958°W
*Bembidion tetrapholeon*	2565	ARGENTINA: Neuquén: Rip Pichi Traful nr Lago Traful, 810m, 40.4867°S, 71.5958°W
*Bembidion tetrapholeon*	2566	ARGENTINA: Neuquén: Arroyo Queñi at Lago Queñi, 830m, 40.1575°S, 71.7210°W
*Bembidion tucumanum*	1430	ARGENTINA: Santa Cruz District, Dept. of Deseado, Cañadón Minerales. 25 km S. of Caleta Olivia. 46.7146°S, 67.367°W. 25m.
*Bembidion walterrossii*	2650	ECUADOR: Napo: Vinillos, 4.1 km S Cosanga, 2090m, 0.6024°S, 77.8509°W
*Bembidion (Ecuadion)* sp. “*Mendoza*”	2701	ARGENTINA: Mendoza: Reserva Villavicencio, 1540m, 32.5232°S, 68.9949°W
*Bembidion (Ecuadion)* sp. “*Papallacta*”	2657	ECUADOR: Napo: Papallacta, 3315m, 0.3703°S, 78.1481°W
*Bembidion (Notholopha)* sp. “*Nahuelbuta*”	2239	CHILE: Reg. IX: P.N. Nahuelbuta, 1090m, 37.8274°S, 73.0096°W

## Supplementary materials

Supplementary material 1 NEXUS file containing DNA sequence matrices and maximum likelihood trees doi: 10.3897/zookeys.416.7706.app1 Data type: NEXUS file with DNA data and trees Explanation note: This is a NEXUS file containing the seven individual gene matrices as well as the two matrices of the concatenated genes. In addition, it contains the maximum likelihood trees for each matrix, and all of the bootstrap trees (2000 trees for each matrix). The file is formatted to be opened in Mesquite 2.75 or later. This dataset is made available under the Open Database License (http://opendatacommons.org/licenses/odbl/1.0/). The Open Database License (ODbL) is a license agreement intended to allow users to freely share, modify, and use this Dataset while maintaining this same freedom for others, provided that the original source and author(s) are credited.David R. Maddison

Supplementary material 2 NEXUS file containing DNA sequences for *Bembidion tetrapholeon* doi: 10.3897/zookeys.416.7706.app2 Data type: NEXUS file with DNA data Explanation note: This is a NEXUS file containing six matrices showing observed sequences for all of the specimens of *Bembidion tetrapholeon* sequenced. The file is formatted to be opened in Mesquite 2.75 or later. This dataset is made available under the Open Database License (http://opendatacommons.org/licenses/odbl/1.0/). The Open Database License (ODbL) is a license agreement intended to allow users to freely share, modify, and use this Dataset while maintaining this same freedom for others, provided that the original source and author(s) are credited.David R. Maddison

Supplementary material 3 Images of maximum likelihood trees doi: 10.3897/zookeys.416.7706.app3 Data type: images of phylogenetic trees Explanation note: This file shows the maximum likelihood trees for the concatenated, seven-gene matrices as well as each individual gene. Each figure is labeled to indicate the nature of the data analyzed for that tree. This dataset is made available under the Open Database License (http://opendatacommons.org/licenses/odbl/1.0/). The Open Database License (ODbL) is a license agreement intended to allow users to freely share, modify, and use this Dataset while maintaining this same freedom for others, provided that the original source and author(s) are credited.David R. Maddison

Supplementary material 4 Images of maximum likelihood bootstrap trees doi: 10.3897/zookeys.416.7706.app4 Data type: images of phylogenetic trees Explanation note: This file shows the maximum likelihood bootstrap trees for the concatenated, 7-gene matrices as well as each individual gene. Each figure is labeled to indicate the nature of the data analyzed for that tree. Numbers on each branch are the frequencies of that clade in the bootstrap replicates, expressed as a percentage. This dataset is made available under the Open Database License (http://opendatacommons.org/licenses/odbl/1.0/). The Open Database License (ODbL) is a license agreement intended to allow users to freely share, modify, and use this Dataset while maintaining this same freedom for others, provided that the original source and author(s) are credited.David R. Maddison
