# Supplementary material for: An unexpected clade of South American ground beetles (Coleoptera, Carabidae, Bembidion)
Source: Zookeys. 2014 Jun 17;(416):113–55. doi: 10.3897/zookeys.416.7706 (PMC4109512; doi:10.3897/zookeys.416.7706)
Supplement: Supplementary material 3 — Images of maximum likelihood trees [file zookeys-416-113-s003.pdf]

**Supporting Information S3**

This file shows the maximum likelihood trees for the concatenated, 7-gene matrices as well as each individual gene. Each figure is labeled to indicate the nature of that data analyzed for that tree.

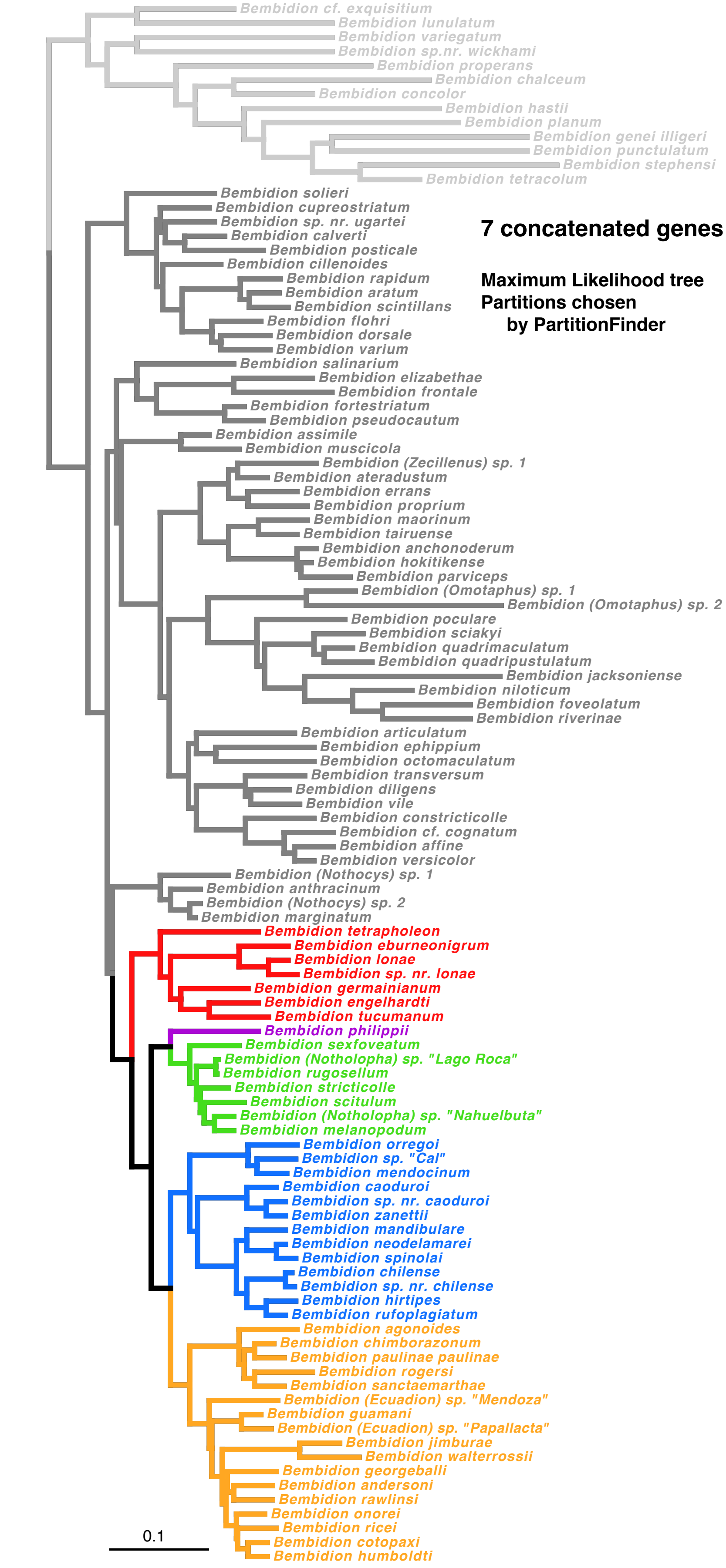

7 concatenated genes
Maximum Likelihood tree
Partitions chosen
by PartitionFinder
Bembidion cf. exquisitium
Bembidion lunulatum
Bembidion variegatum
Bembidion sp.nr. wickhami
Bembidion properans
Bembidion chalceum
Bembidion concolor
Bembidion hastii
Bembidion planum
Bembidion genei illigeri
Bembidion punctulatum
Bembidion stephensi
Bembidion tetracolum
Bembidion solieri
Bembidion cupreostriatum
Bembidion sp. nr. ugartei
Bembidion calverti
Bembidion posticale
Bembidion cillenoides
Bembidion rapidum
Bembidion aratum
Bembidion scintillans
Bembidion flohri
Bembidion dorsale
Bembidion varium
Bembidion salinarium
Bembidion elizabethae
Bembidion frontale
Bembidion fortestriatum
Bembidion pseudocautum
Bembidion assimile
Bembidion muscicola
Bembidion (Zecillenus) sp. 1
Bembidion ateradustum
Bembidion errans
Bembidion proprium
Bembidion maorinum
Bembidion tairuense
Bembidion anchonoderum
Bembidion hokitikense
Bembidion parviceps
Bembidion (Omotaphus) sp. 1
Bembidion (Omotaphus) sp. 2
Bembidion poculare
Bembidion sciakyi
Bembidion quadrimaculatum
Bembidion quadripustulatum
Bembidion jacksoniense
Bembidion niloticum
Bembidion foveolatum
Bembidion riverinae
Bembidion articulatum
Bembidion ephippium
Bembidion octomaculatum
Bembidion transversum
Bembidion diligens
Bembidion vile
Bembidion constricticolle
Bembidion cf. cognatum
Bembidion affine
Bembidion versicolor
Bembidion (Nothocys) sp. 1
Bembidion anthracinum
Bembidion (Nothocys) sp. 2
Bembidion marginatum
Bembidion tetrapholeon
Bembidion eburneonigrum
Bembidion lonae
Bembidion sp. nr. lonae
Bembidion germainianum
Bembidion engelhardti
Bembidion tucumanum
Bembidion philippii
Bembidion sexfoveatum
Bembidion (Notholopha) sp. "Lago Roca"
Bembidion rugosellum
Bembidion stricticolle
Bembidion scitulum
Bembidion (Notholopha) sp. "Nahuelbuta"
Bembidion melanopodum
Bembidion orregoi
Bembidion sp. "Cal"
Bembidion mendocinum
Bembidion caoduroi
Bembidion sp. nr. caoduroi
Bembidion zanettii
Bembidion mandibulare
Bembidion neodelamarei
Bembidion spinolai
Bembidion chilense
Bembidion sp. nr. chilense
Bembidion hirtipes
Bembidion rufoplagiatum
Bembidion agonoides
Bembidion chimborazonum
Bembidion paulinae paulinae
Bembidion rogersi
Bembidion sanctaemarthae
Bembidion (Ecuadion) sp. "Mendoza"
Bembidion guamani
Bembidion (Ecuadion) sp. "Papallacta"
Bembidion jimburae
Bembidion walterrossii
Bembidion georgeballi
Bembidion andersoni
Bembidion rawlinsi
Bembidion onorei
Bembidion ricei
Bembidion cotopaxi
Bembidion humboldti
0.1

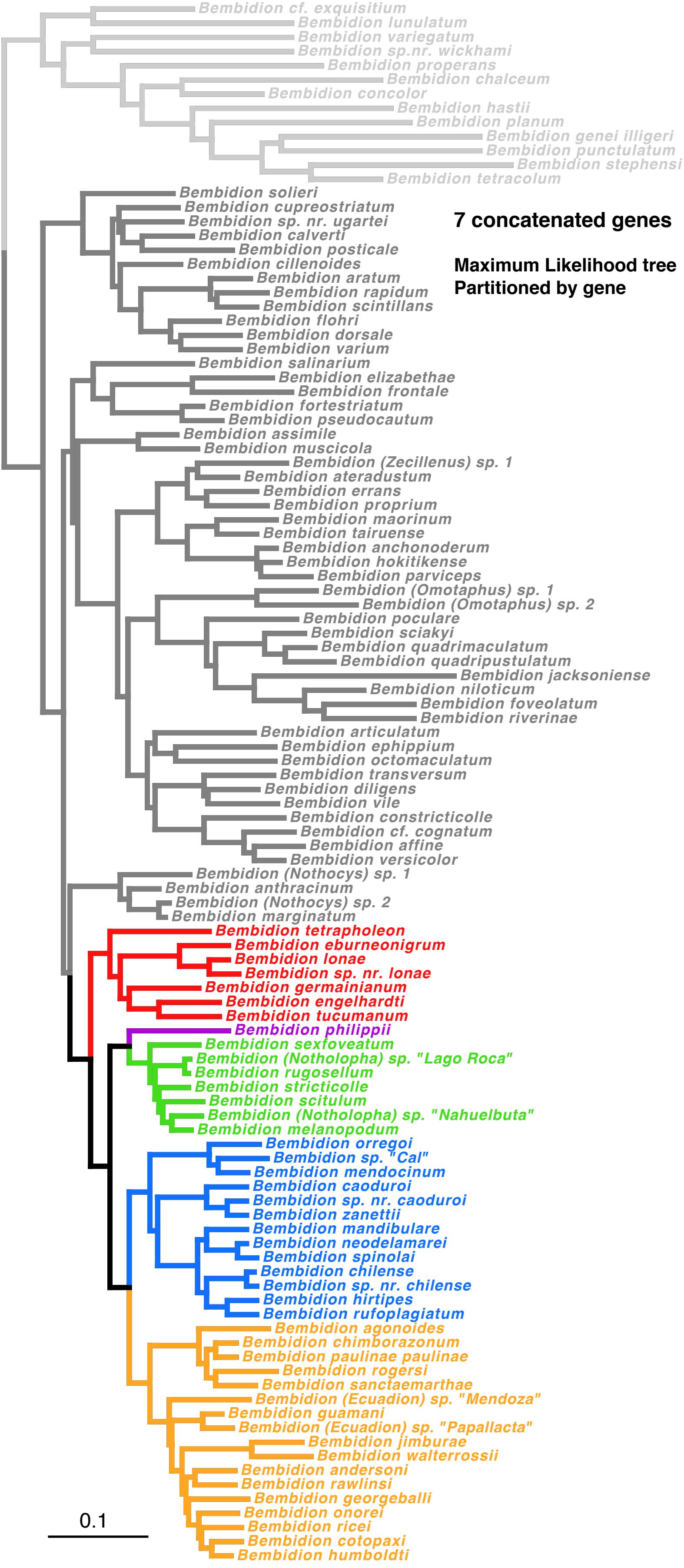

Bembidion cf. exquisitium
Bembidion lunulatum
Bembidion variegatum
Bembidion sp.nr. wickhami
Bembidion properans
Bembidion chalceum
Bembidion concolor
Bembidion hastii
Bembidion planum
Bembidion genei illigeri
Bembidion punctulatum
Bembidion stephensi
Bembidion tetracolum
Bembidion solieri
Bembidion cupreostriatum
Bembidion sp. nr. ugartei
Bembidion calverti
Bembidion posticale
Bembidion cillenoides
Bembidion aratum
Bembidion rapidum
Bembidion scintillans
Bembidion flohri
Bembidion dorsale
Bembidion varium
Bembidion salinarium
Bembidion elizabethae
Bembidion frontale
Bembidion fortestriatum
Bembidion pseudocautum
Bembidion assimile
Bembidion muscicola
Bembidion (Zecillenus) sp. 1
Bembidion ateradustum
Bembidion errans
Bembidion proprium
Bembidion maorinum
Bembidion tairuense
Bembidion anchonoderum
Bembidion hokitikense
Bembidion parviceps
Bembidion (Omotaphus) sp. 1
Bembidion (Omotaphus) sp. 2
Bembidion poculare
Bembidion sciakyi
Bembidion quadrimaculatum
Bembidion quadripustulatum
Bembidion jacksoniense
Bembidion niloticum
Bembidion foveolatum
Bembidion riverinae
Bembidion articulatum
Bembidion ephippium
Bembidion octomaculatum
Bembidion transversum
Bembidion diligens
Bembidion vile
Bembidion constricticolle
Bembidion cf. cognatum
Bembidion affine
Bembidion versicolor
Bembidion (Nothocys) sp. 1
Bembidion anthracinum
Bembidion (Nothocys) sp. 2
Bembidion marginatum
Bembidion tetrapholeon
Bembidion eburneonigrum
Bembidion lonae
Bembidion sp. nr. lonae
Bembidion germainianum
Bembidion engelhardti
Bembidion tucumanum
Bembidion philippii
Bembidion sexfoveatum
Bembidion (Notholopha) sp. "Lago Roca"
Bembidion rugosellum
Bembidion stricticolle
Bembidion scitulum
Bembidion (Notholopha) sp. "Nahuelbuta"
Bembidion melanopodum
Bembidion orregoi
Bembidion sp. "Cal"
Bembidion mendocinum
Bembidion caoduroi
Bembidion sp. nr. caoduroi
Bembidion zanettii
Bembidion mandibulare
Bembidion neodelamarei
Bembidion spinolai
Bembidion chilense
Bembidion sp. nr. chilense
Bembidion hirtipes
Bembidion rufoplagiatum
Bembidion agonoides
Bembidion chimborazonum
Bembidion paulinae paulinae
Bembidion rogersi
Bembidion sanctaemarthae
Bembidion (Ecuadion) sp. "Mendoza"
Bembidion guamani
Bembidion (Ecuadion) sp. "Papallacta"
Bembidion jimburae
Bembidion walterrossii
Bembidion andersoni
Bembidion rawlinsi
Bembidion georgeballi
Bembidion onorei
Bembidion ricei
Bembidion cotopaxi
Bembidion humboldti
7 concatenated genes
Maximum Likelihood tree
Partitioned by gene
0.1

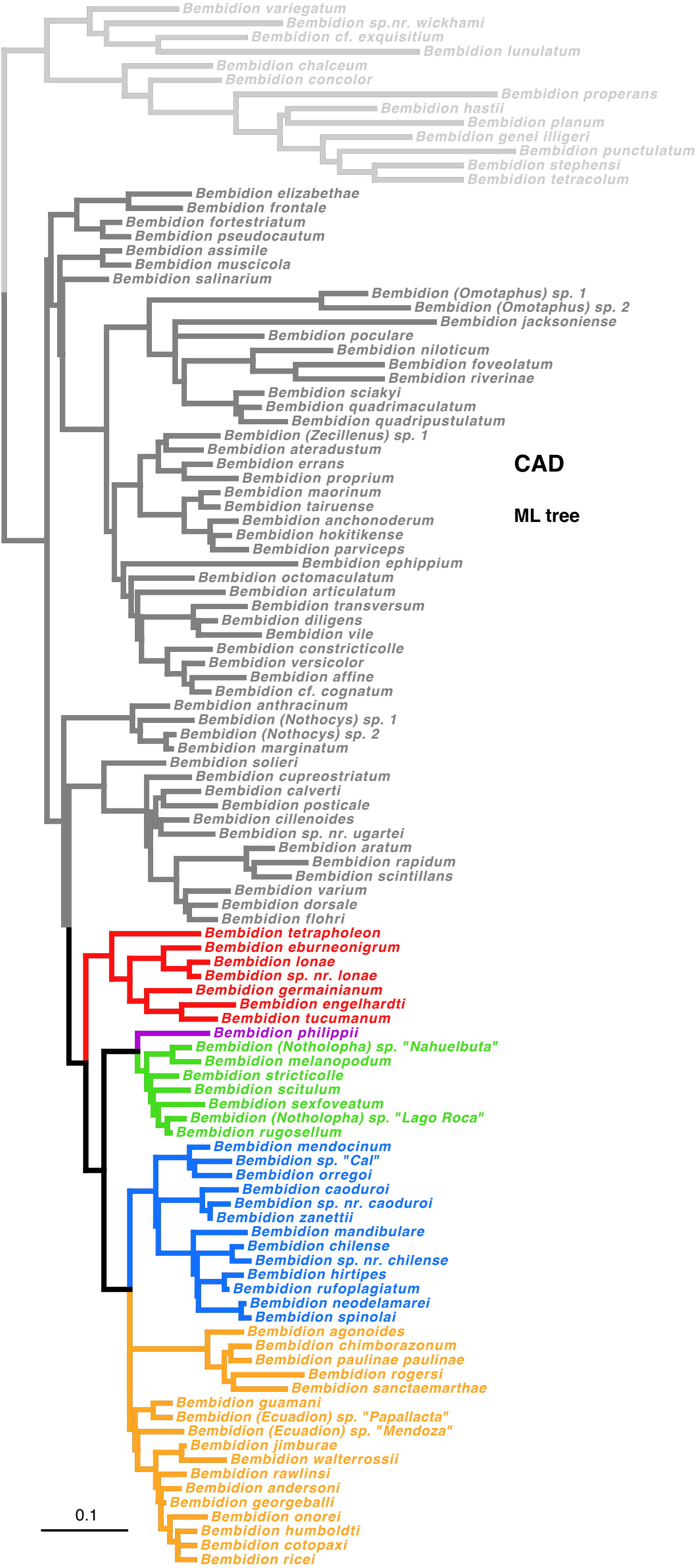
CAD
ML tree
0.1
Bembidion variegatum
Bembidion sp.nr. wickhami
Bembidion cf. exquisitium
Bembidion lunulatum
Bembidion chalceum
Bembidion concolor
Bembidion properans
Bembidion hastii
Bembidion planum
Bembidion genei illigeri
Bembidion punctulatum
Bembidion stephensi
Bembidion tetracolum
Bembidion elizabethae
Bembidion frontale
Bembidion fortestriatum
Bembidion pseudocautum
Bembidion assimile
Bembidion muscicola
Bembidion salinarium
Bembidion (Omotaphus) sp. 1
Bembidion (Omotaphus) sp. 2
Bembidion jacksoniense
Bembidion poculare
Bembidion niloticum
Bembidion foveolatum
Bembidion riverinae
Bembidion sciakyi
Bembidion quadrimaculatum
Bembidion quadripustulatum
Bembidion (Zecillenus) sp. 1
Bembidion ateradustum
Bembidion errans
Bembidion proprium
Bembidion maorinum
Bembidion tairuense
Bembidion anchonoderum
Bembidion hokitikense
Bembidion parviceps
Bembidion ephippium
Bembidion octomaculatum
Bembidion articulatum
Bembidion transversum
Bembidion diligens
Bembidion vile
Bembidion constricticolle
Bembidion versicolor
Bembidion affine
Bembidion cf. cognatum
Bembidion anthracinum
Bembidion (Nothocys) sp. 1
Bembidion (Nothocys) sp. 2
Bembidion marginatum
Bembidion solieri
Bembidion cupreostriatum
Bembidion calverti
Bembidion posticale
Bembidion cillenoides
Bembidion sp. nr. ugartei
Bembidion aratum
Bembidion rapidum
Bembidion scintillans
Bembidion varium
Bembidion dorsale
Bembidion flohri
Bembidion tetrapholeon
Bembidion eburneonigrum
Bembidion lonae
Bembidion sp. nr. lonae
Bembidion germainianum
Bembidion engelhardti
Bembidion tucumanum
Bembidion philippii
Bembidion (Notholopha) sp. "Nahuelbuta"
Bembidion melanopodum
Bembidion stricticolle
Bembidion scitulum
Bembidion sexfoveatum
Bembidion (Notholopha) sp. "Lago Roca"
Bembidion rugosellum
Bembidion mendocinum
Bembidion sp. "Cal"
Bembidion orregoi
Bembidion caoduroi
Bembidion sp. nr. caoduroi
Bembidion zanettii
Bembidion mandibulare
Bembidion chilense
Bembidion sp. nr. chilense
Bembidion hirtipes
Bembidion rufoplagiatum
Bembidion neodelamarei
Bembidion spinolai
Bembidion agonoides
Bembidion chimborazonum
Bembidion paulinae paulinae
Bembidion rogersi
Bembidion sanctaemarthae
Bembidion guamani
Bembidion (Ecuadion) sp. "Papallacta"
Bembidion (Ecuadion) sp. "Mendoza"
Bembidion jimburae
Bembidion walterrossii
Bembidion rawlinsi
Bembidion andersoni
Bembidion georgeballi
Bembidion onorei
Bembidion humboldti
Bembidion cotopaxi
Bembidion ricei

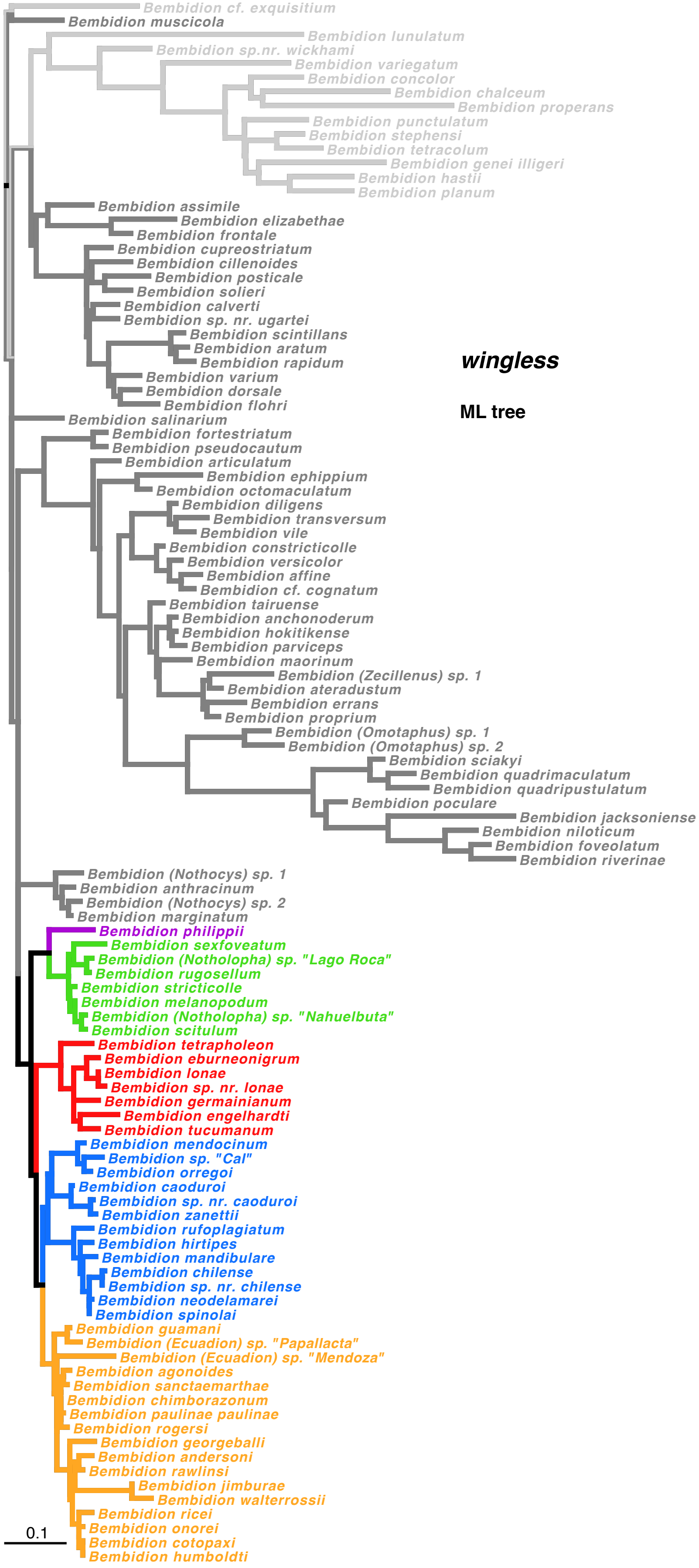
Bembidion cf. exquisitium
Bembidion muscicola
Bembidion lunulatum
Bembidion sp.nr. wickhami
Bembidion variegatum
Bembidion concolor
Bembidion chalceum
Bembidion properans
Bembidion punctulatum
Bembidion stephensi
Bembidion tetracolum
Bembidion genei illigeri
Bembidion hastii
Bembidion planum
Bembidion assimile
Bembidion elizabethae
Bembidion frontale
Bembidion cupreostriatum
Bembidion cillenoides
Bembidion posticale
Bembidion solieri
Bembidion calverti
Bembidion sp. nr. ugartei
Bembidion scintillans
Bembidion aratum
Bembidion rapidum
Bembidion varium
Bembidion dorsale
Bembidion flohri
Bembidion salinarium
Bembidion fortestriatum
Bembidion pseudocautum
Bembidion articulatum
Bembidion ephippium
Bembidion octomaculatum
Bembidion diligens
Bembidion transversum
Bembidion vile
Bembidion constricticolle
Bembidion versicolor
Bembidion affine
Bembidion cf. cognatum
Bembidion tairuense
Bembidion anchonoderum
Bembidion hokitikense
Bembidion parviceps
Bembidion maorinum
Bembidion (Zecillenus) sp. 1
Bembidion ateradustum
Bembidion errans
Bembidion proprium
Bembidion (Omotaphus) sp. 1
Bembidion (Omotaphus) sp. 2
Bembidion sciakyi
Bembidion quadrimaculatum
Bembidion quadripustulatum
Bembidion poculare
Bembidion jacksoniense
Bembidion niloticum
Bembidion foveolatum
Bembidion riverinae
Bembidion (Nothocys) sp. 1
Bembidion anthracinum
Bembidion (Nothocys) sp. 2
Bembidion marginatum
Bembidion philippii
Bembidion sexfoveatum
Bembidion (Notholopha) sp. "Lago Roca"
Bembidion rugosellum
Bembidion stricticolle
Bembidion melanopodum
Bembidion (Notholopha) sp. "Nahuelbuta"
Bembidion scitulum
Bembidion tetrapholeon
Bembidion eburneonigrum
Bembidion lonae
Bembidion sp. nr. lonae
Bembidion germainianum
Bembidion engelhardti
Bembidion tucumanum
Bembidion mendocinum
Bembidion sp. "Cal"
Bembidion orregoi
Bembidion caoduroi
Bembidion sp. nr. caoduroi
Bembidion zanettii
Bembidion rufoplagiatum
Bembidion hirtipes
Bembidion mandibulare
Bembidion chilense
Bembidion sp. nr. chilense
Bembidion neodelamarei
Bembidion spinolai
Bembidion guamani
Bembidion (Ecuadion) sp. "Papallacta"
Bembidion (Ecuadion) sp. "Mendoza"
Bembidion agonoides
Bembidion sanctaemarthae
Bembidion chimborazonum
Bembidion paulinae paulinae
Bembidion rogersi
Bembidion georgeballi
Bembidion andersoni
Bembidion rawlinsi
Bembidion jimburae
Bembidion walterrossii
Bembidion ricei
Bembidion onorei
Bembidion cotopaxi
Bembidion humboldti
wingless
ML tree
0.1

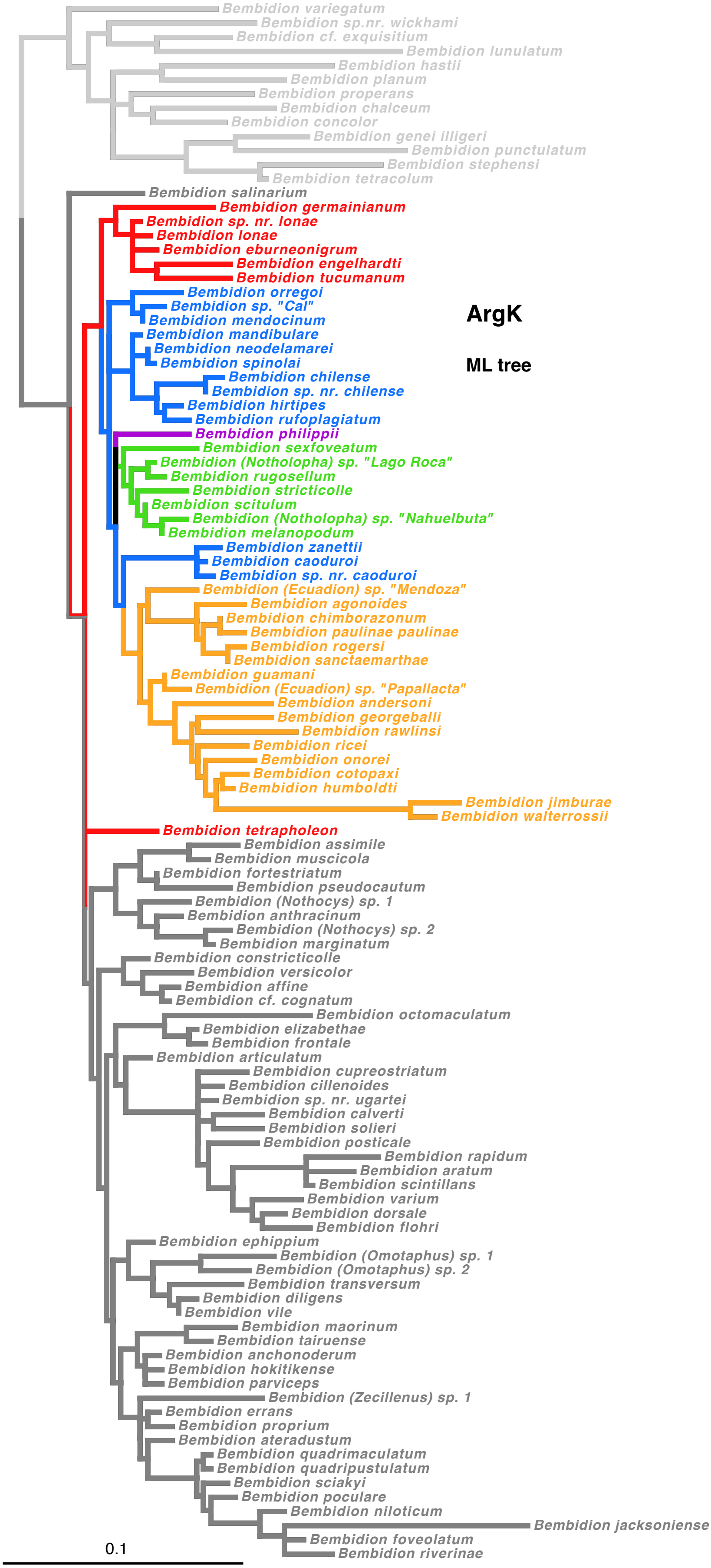
Bembidion variegatum
Bembidion sp.nr. wickhami
Bembidion cf. exquisitium
Bembidion lunulatum
Bembidion hastii
Bembidion planum
Bembidion properans
Bembidion chalceum
Bembidion concolor
Bembidion genei illigeri
Bembidion punctulatum
Bembidion stephensi
Bembidion tetracolum
Bembidion salinarium
Bembidion germainianum
Bembidion sp. nr. lonae
Bembidion lonae
Bembidion eburneonigrum
Bembidion engelhardti
Bembidion tucumanum
Bembidion orregoi
Bembidion sp. "Cal"
Bembidion mendocinum
Bembidion mandibulare
Bembidion neodelamarei
Bembidion spinolai
Bembidion chilense
Bembidion sp. nr. chilense
Bembidion hirtipes
Bembidion rufoplagiatum
Bembidion philippii
Bembidion sexfoveatum
Bembidion (Notholopha) sp. "Lago Roca"
Bembidion rugosellum
Bembidion stricticolle
Bembidion scitulum
Bembidion (Notholopha) sp. "Nahuelbuta"
Bembidion melanopodum
Bembidion zanettii
Bembidion caoduroi
Bembidion sp. nr. caoduroi
Bembidion (Ecuadion) sp. "Mendoza"
Bembidion agonoides
Bembidion chimborazonum
Bembidion paulinae paulinae
Bembidion rogersi
Bembidion sanctaemarthae
Bembidion guamani
Bembidion (Ecuadion) sp. "Papallacta"
Bembidion andersoni
Bembidion georgeballi
Bembidion rawlinsi
Bembidion ricei
Bembidion onorei
Bembidion cotopaxi
Bembidion humboldti
Bembidion jimburae
Bembidion walterrossii
Bembidion tetrapholeon
Bembidion assimile
Bembidion muscicola
Bembidion fortestriatum
Bembidion pseudocautum
Bembidion (Nothocys) sp. 1
Bembidion anthracinum
Bembidion (Nothocys) sp. 2
Bembidion marginatum
Bembidion constricticolle
Bembidion versicolor
Bembidion affine
Bembidion cf. cognatum
Bembidion octomaculatum
Bembidion elizabethae
Bembidion frontale
Bembidion articulatum
Bembidion cupreostriatum
Bembidion cillenoides
Bembidion sp. nr. ugartei
Bembidion calverti
Bembidion solieri
Bembidion posticale
Bembidion rapidum
Bembidion aratum
Bembidion scintillans
Bembidion varium
Bembidion dorsale
Bembidion flohri
Bembidion ephippium
Bembidion (Omotaphus) sp. 1
Bembidion (Omotaphus) sp. 2
Bembidion transversum
Bembidion diligens
Bembidion vile
Bembidion maorinum
Bembidion tairuense
Bembidion anchonoderum
Bembidion hokitikense
Bembidion parviceps
Bembidion (Zecillenus) sp. 1
Bembidion errans
Bembidion proprium
Bembidion ateradustum
Bembidion quadrimaculatum
Bembidion quadripustulatum
Bembidion sciakyi
Bembidion poculare
Bembidion niloticum
Bembidion jacksoniense
Bembidion foveolatum
Bembidion riverinae
ArgK
ML tree
0.1

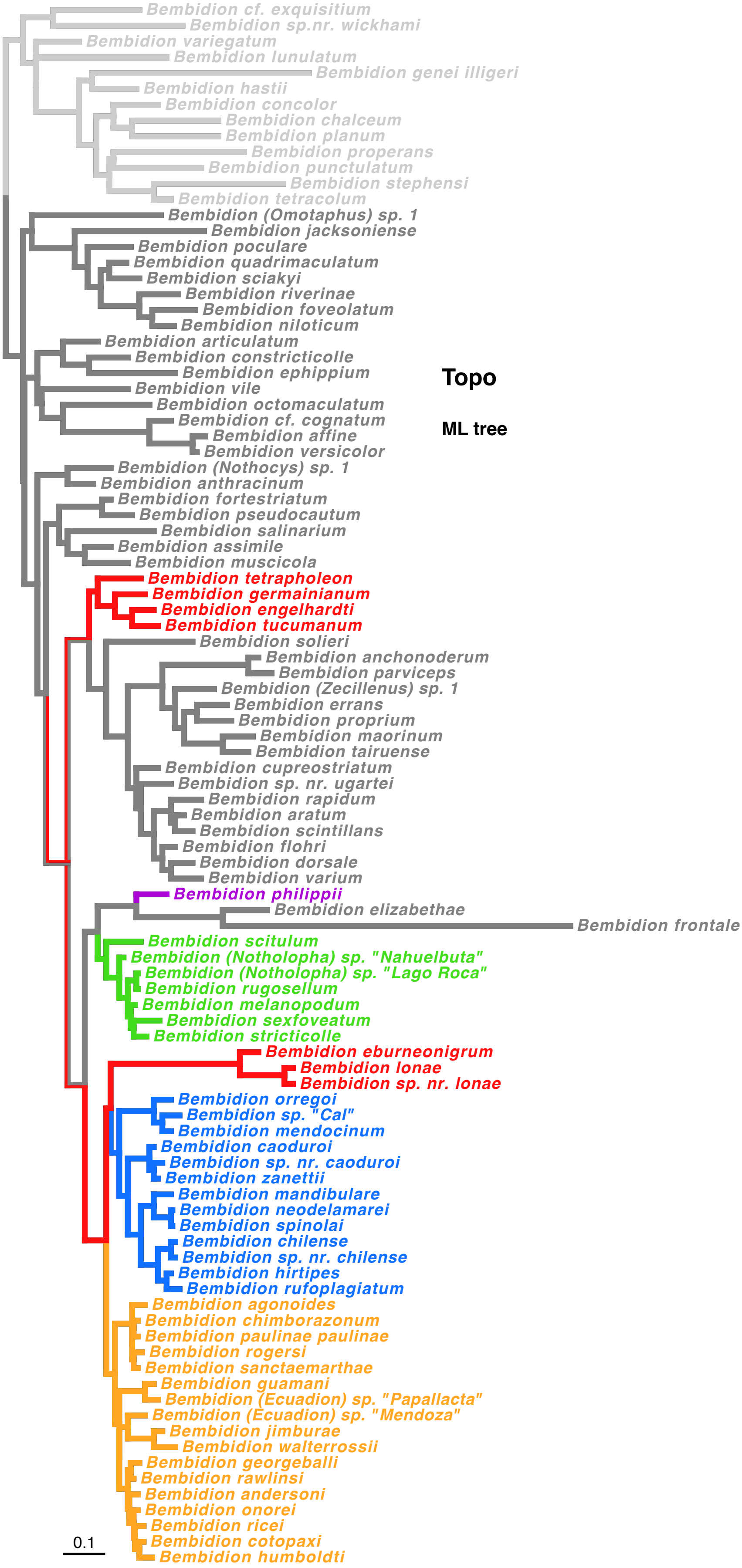
Bembidion cf. exquisitium
Bembidion sp.nr. wickhami
Bembidion variegatum
Bembidion lunulatum
Bembidion genei illigeri
Bembidion hastii
Bembidion concolor
Bembidion chalceum
Bembidion planum
Bembidion properans
Bembidion punctulatum
Bembidion stephensi
Bembidion tetracolum
Bembidion (Omotaphus) sp. 1
Bembidion jacksoniense
Bembidion poculare
Bembidion quadrimaculatum
Bembidion sciakyi
Bembidion riverinae
Bembidion foveolatum
Bembidion niloticum
Bembidion articulatum
Bembidion constricticolle
Bembidion ephippium
Bembidion vile
Bembidion octomaculatum
Bembidion cf. cognatum
Bembidion affine
Bembidion versicolor
Bembidion (Nothocys) sp. 1
Bembidion anthracinum
Bembidion fortestriatum
Bembidion pseudocautum
Bembidion salinarium
Bembidion assimile
Bembidion muscicola
Bembidion tetrapholeon
Bembidion germainianum
Bembidion engelhardti
Bembidion tucumanum
Bembidion solieri
Bembidion anchonoderum
Bembidion parviceps
Bembidion (Zecillenus) sp. 1
Bembidion errans
Bembidion proprium
Bembidion maorinum
Bembidion tairuense
Bembidion cupreostriatum
Bembidion sp. nr. ugartei
Bembidion rapidum
Bembidion aratum
Bembidion scintillans
Bembidion flohri
Bembidion dorsale
Bembidion varium
Bembidion philippii
Bembidion elizabethae
Bembidion frontale
Bembidion scitulum
Bembidion (Notholopha) sp. "Nahuelbuta"
Bembidion (Notholopha) sp. "Lago Roca"
Bembidion rugosellum
Bembidion melanopodum
Bembidion sexfoveatum
Bembidion stricticolle
Bembidion eburneonigrum
Bembidion lonae
Bembidion sp. nr. lonae
Bembidion orregoi
Bembidion sp. "Cal"
Bembidion mendocinum
Bembidion caoduroi
Bembidion sp. nr. caoduroi
Bembidion zanettii
Bembidion mandibulare
Bembidion neodelamarei
Bembidion spinolai
Bembidion chilense
Bembidion sp. nr. chilense
Bembidion hirtipes
Bembidion rufoplagiatum
Bembidion agonoides
Bembidion chimborazonum
Bembidion paulinae paulinae
Bembidion rogersi
Bembidion sanctaemarthae
Bembidion guamani
Bembidion (Ecuadion) sp. "Papallacta"
Bembidion (Ecuadion) sp. "Mendoza"
Bembidion jimburae
Bembidion walterrossii
Bembidion georgeballi
Bembidion rawlinsi
Bembidion andersoni
Bembidion onorei
Bembidion ricei
Bembidion cotopaxi
Bembidion humboldti
Topo
ML tree
0.1

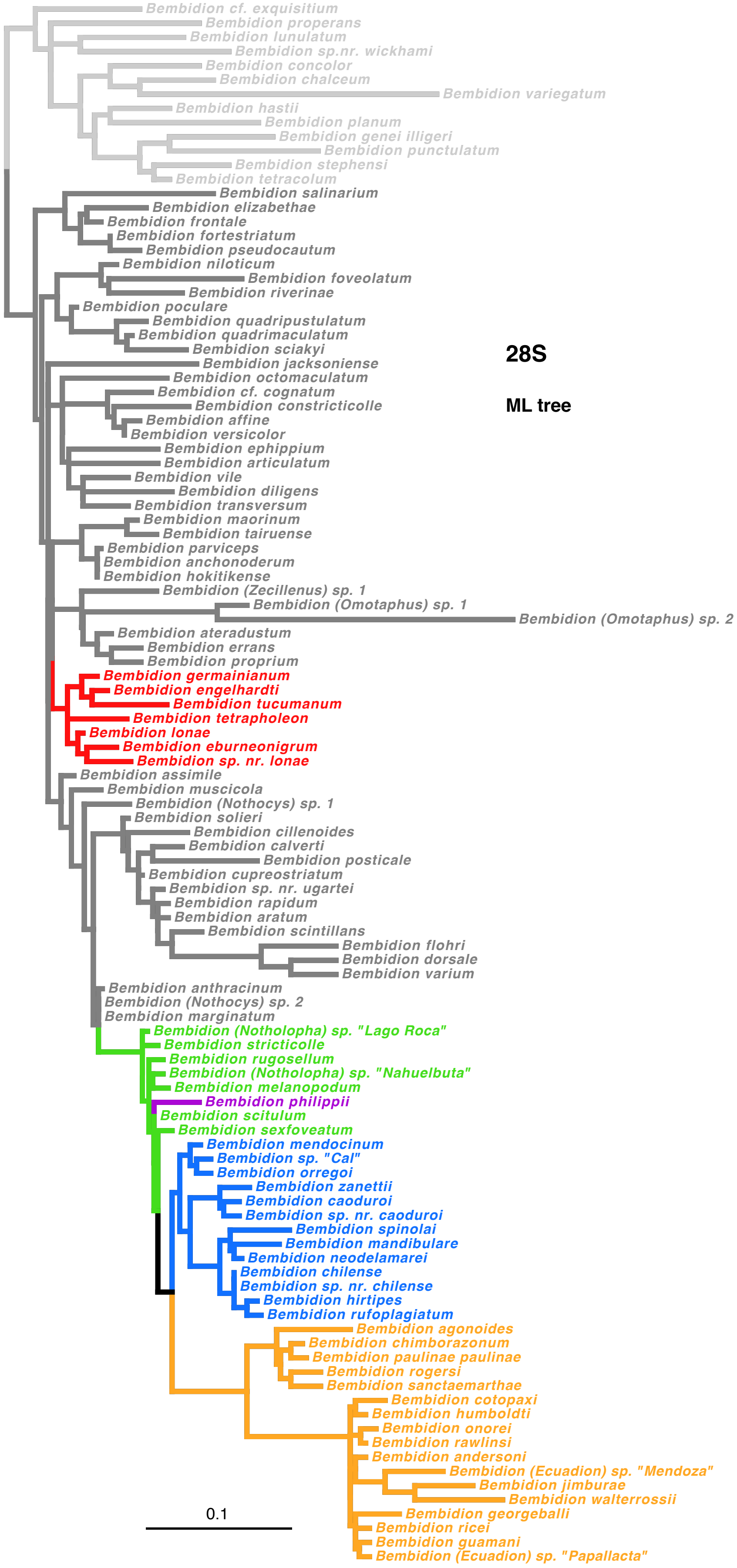
Bembidion cf. exquisitium
Bembidion properans
Bembidion lunulatum
Bembidion sp.nr. wickhami
Bembidion concolor
Bembidion chalceum
Bembidion variegatum
Bembidion hastii
Bembidion planum
Bembidion genei illigeri
Bembidion punctulatum
Bembidion stephensi
Bembidion tetracolum
Bembidion salinarium
Bembidion elizabethae
Bembidion frontale
Bembidion fortestriatum
Bembidion pseudocautum
Bembidion niloticum
Bembidion foveolatum
Bembidion riverinae
Bembidion poculare
Bembidion quadripustulatum
Bembidion quadrimaculatum
Bembidion sciakyi
Bembidion jacksoniense
Bembidion octomaculatum
Bembidion cf. cognatum
Bembidion constricticolle
Bembidion affine
Bembidion versicolor
Bembidion ephippium
Bembidion articulatum
Bembidion vile
Bembidion diligens
Bembidion transversum
Bembidion maorinum
Bembidion tairuense
Bembidion parviceps
Bembidion anchonoderum
Bembidion hokitikense
Bembidion (Zecillenus) sp. 1
Bembidion (Omotaphus) sp. 1
Bembidion (Omotaphus) sp. 2
Bembidion ateradustum
Bembidion errans
Bembidion proprium
Bembidion germainianum
Bembidion engelhardti
Bembidion tucumanum
Bembidion tetrapholeon
Bembidion lonae
Bembidion eburneonigrum
Bembidion sp. nr. lonae
Bembidion assimile
Bembidion muscicola
Bembidion (Nothocys) sp. 1
Bembidion solieri
Bembidion cillenoides
Bembidion calverti
Bembidion posticale
Bembidion cupreostriatum
Bembidion sp. nr. ugartei
Bembidion rapidum
Bembidion aratum
Bembidion scintillans
Bembidion flohri
Bembidion dorsale
Bembidion varium
Bembidion anthracinum
Bembidion (Nothocys) sp. 2
Bembidion marginatum
Bembidion (Notholopha) sp. "Lago Roca"
Bembidion stricticolle
Bembidion rugosellum
Bembidion (Notholopha) sp. "Nahuelbuta"
Bembidion melanopodum
Bembidion philippii
Bembidion scitulum
Bembidion sexfoveatum
Bembidion mendocinum
Bembidion sp. "Cal"
Bembidion orregoi
Bembidion zanettii
Bembidion caoduroi
Bembidion sp. nr. caoduroi
Bembidion spinolai
Bembidion mandibulare
Bembidion neodelamarei
Bembidion chilense
Bembidion sp. nr. chilense
Bembidion hirtipes
Bembidion rufoplagiatum
Bembidion agonoides
Bembidion chimborazonum
Bembidion paulinae paulinae
Bembidion rogersi
Bembidion sanctaemarthae
Bembidion cotopaxi
Bembidion humboldti
Bembidion onorei
Bembidion rawlinsi
Bembidion andersoni
Bembidion (Ecuadion) sp. "Mendoza"
Bembidion jimburae
Bembidion walterrossii
Bembidion georgeballi
Bembidion ricei
Bembidion guamani
Bembidion (Ecuadion) sp. "Papallacta"
28S
ML tree
0.1

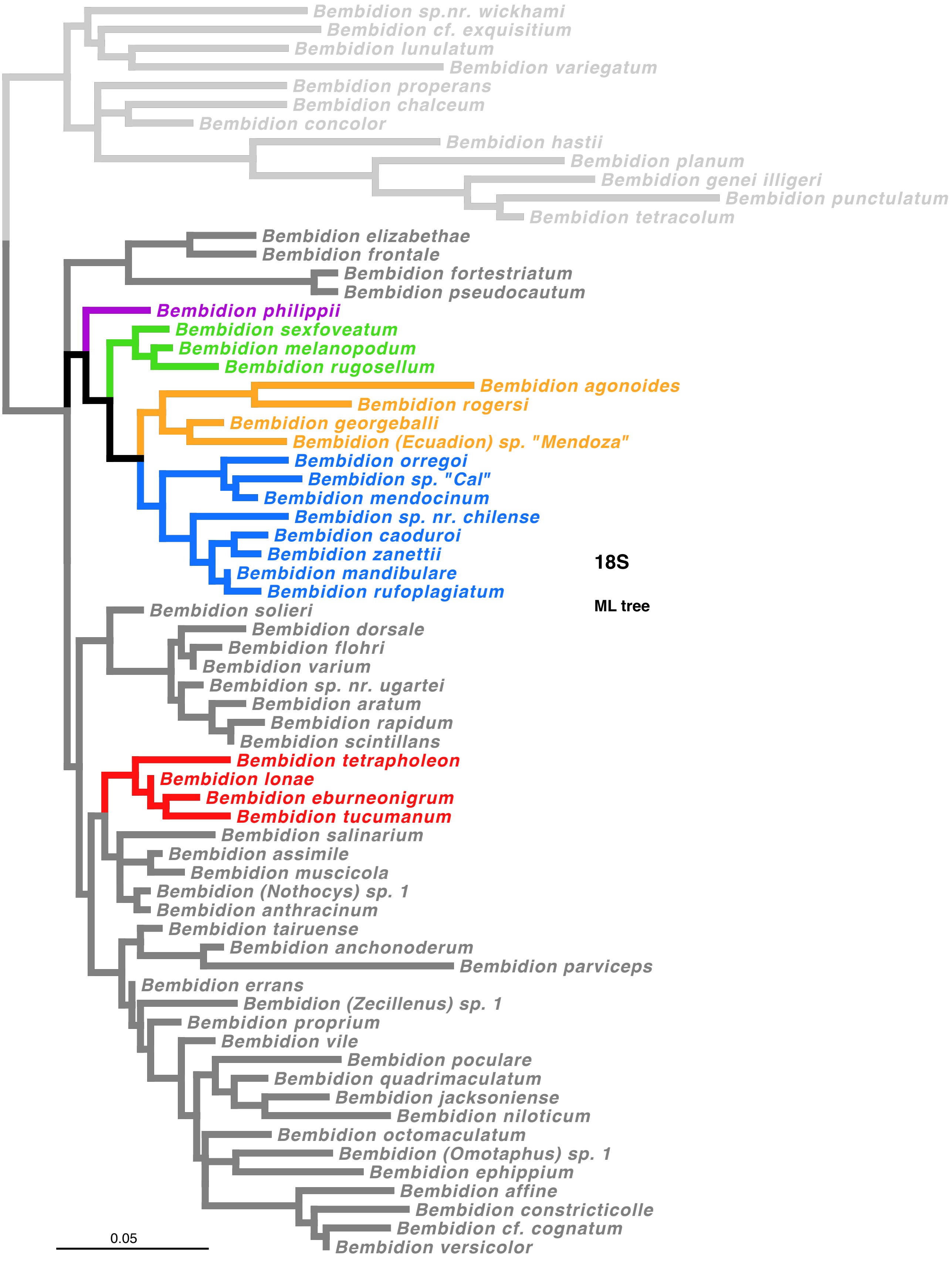
Bembidion sp.nr. wickhami
Bembidion cf. exquisitium
Bembidion lunulatum
Bembidion variegatum
Bembidion properans
Bembidion chalceum
Bembidion concolor
Bembidion hastii
Bembidion planum
Bembidion genei illigeri
Bembidion punctulatum
Bembidion tetracolum
Bembidion elizabethae
Bembidion frontale
Bembidion fortestriatum
Bembidion pseudocautum
Bembidion philippii
Bembidion sexfoveatum
Bembidion melanopodum
Bembidion rugosellum
Bembidion agonoides
Bembidion rogersi
Bembidion georgeballi
Bembidion (Ecuadion) sp. "Mendoza"
Bembidion orregoi
Bembidion sp. "Cal"
Bembidion mendocinum
Bembidion sp. nr. chilense
Bembidion caoduroi
Bembidion zanettii
Bembidion mandibulare
Bembidion rufoplagiatum
18S
ML tree
Bembidion solieri
Bembidion dorsale
Bembidion flohri
Bembidion varium
Bembidion sp. nr. ugartei
Bembidion aratum
Bembidion rapidum
Bembidion scintillans
Bembidion tetrapholeon
Bembidion lonae
Bembidion eburneonigrum
Bembidion tucumanum
Bembidion salinarium
Bembidion assimile
Bembidion muscicola
Bembidion (Nothocys) sp. 1
Bembidion anthracinum
Bembidion tairuense
Bembidion anchonoderum
Bembidion parviceps
Bembidion errans
Bembidion (Zecillenus) sp. 1
Bembidion proprium
Bembidion vile
Bembidion poculare
Bembidion quadrimaculatum
Bembidion jacksoniense
Bembidion niloticum
Bembidion octomaculatum
Bembidion (Omotaphus) sp. 1
Bembidion ephippium
Bembidion affine
Bembidion constricticolle
Bembidion cf. cognatum
Bembidion versicolor
0.05

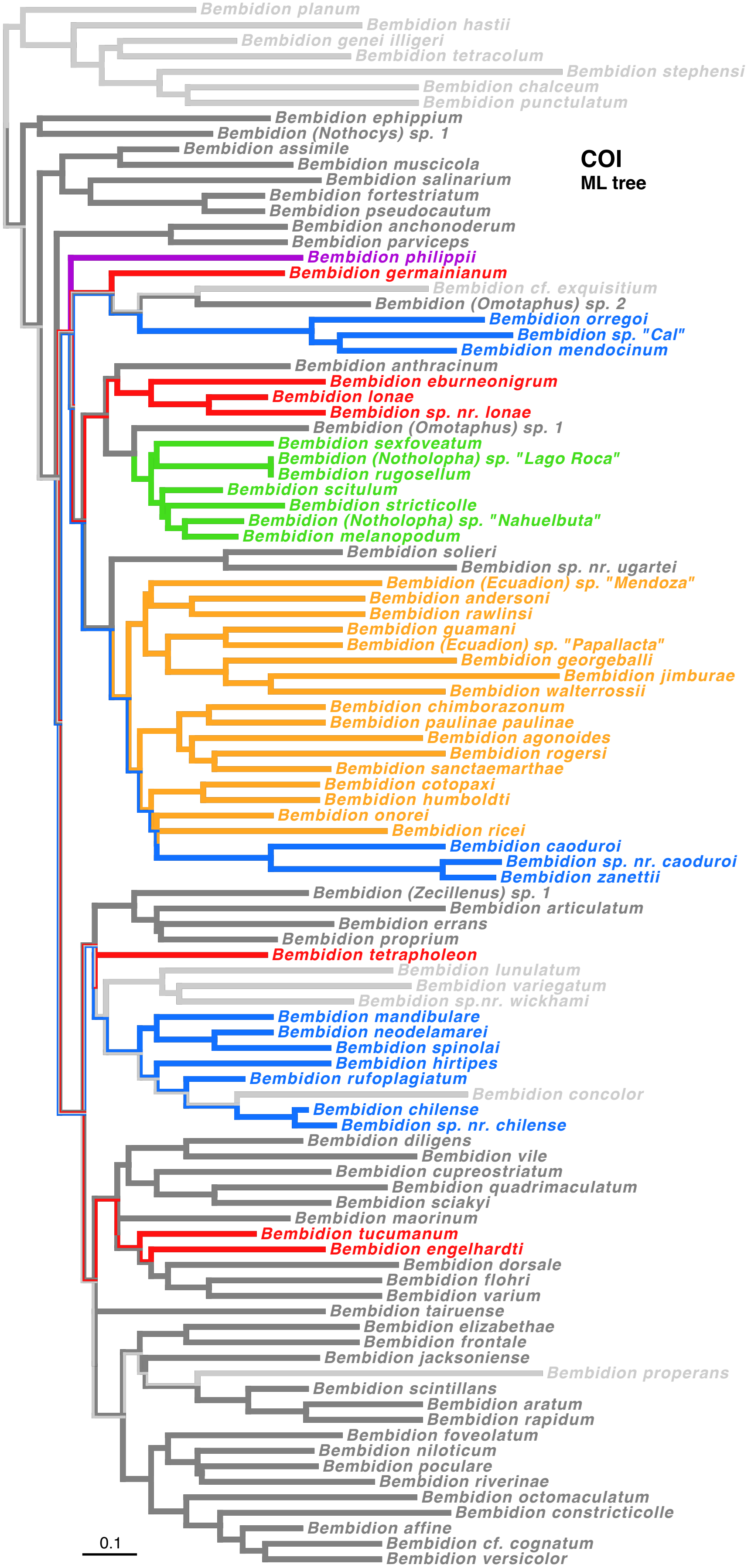
Bembidion planum
Bembidion hastii
Bembidion genei illigeri
Bembidion tetracolum
Bembidion stephensi
Bembidion chalceum
Bembidion punctulatum
Bembidion ephippium
Bembidion (Nothocys) sp. 1
Bembidion assimile
Bembidion muscicola
Bembidion salinarium
Bembidion fortestriatum
Bembidion pseudocautum
Bembidion anchonoderum
Bembidion parviceps
Bembidion philippii
Bembidion germainianum
Bembidion cf. exquisitium
Bembidion (Omotaphus) sp. 2
Bembidion orregoi
Bembidion sp. "Cal"
Bembidion mendocinum
Bembidion anthracinum
Bembidion eburneonigrum
Bembidion lonae
Bembidion sp. nr. lonae
Bembidion (Omotaphus) sp. 1
Bembidion sexfoveatum
Bembidion (Notholopha) sp. "Lago Roca"
Bembidion rugosellum
Bembidion scitulum
Bembidion stricticolle
Bembidion (Notholopha) sp. "Nahuelbuta"
Bembidion melanopodum
Bembidion solieri
Bembidion sp. nr. ugartei
Bembidion (Ecuadion) sp. "Mendoza"
Bembidion andersoni
Bembidion rawlinsi
Bembidion guamani
Bembidion (Ecuadion) sp. "Papallacta"
Bembidion georgeballi
Bembidion jimburae
Bembidion walterrossii
Bembidion chimborazonum
Bembidion paulinae paulinae
Bembidion agonoides
Bembidion rogersi
Bembidion sanctaemarthae
Bembidion cotopaxi
Bembidion humboldti
Bembidion onorei
Bembidion ricei
Bembidion caoduroi
Bembidion sp. nr. caoduroi
Bembidion zanettii
Bembidion (Zecillenus) sp. 1
Bembidion articulatum
Bembidion errans
Bembidion proprium
Bembidion tetrapholeon
Bembidion lunulatum
Bembidion variegatum
Bembidion sp.nr. wickhami
Bembidion mandibulare
Bembidion neodelamarei
Bembidion spinolai
Bembidion hirtipes
Bembidion rufoplagiatum
Bembidion concolor
Bembidion chilense
Bembidion sp. nr. chilense
Bembidion diligens
Bembidion vile
Bembidion cupreostriatum
Bembidion quadrimaculatum
Bembidion sciakyi
Bembidion maorinum
Bembidion tucumanum
Bembidion engelhardti
Bembidion dorsale
Bembidion flohri
Bembidion varium
Bembidion tairuense
Bembidion elizabethae
Bembidion frontale
Bembidion jacksoniense
Bembidion properans
Bembidion scintillans
Bembidion aratum
Bembidion rapidum
Bembidion foveolatum
Bembidion niloticum
Bembidion poculare
Bembidion riverinae
Bembidion octomaculatum
Bembidion constricticolle
Bembidion affine
Bembidion cf. cognatum
Bembidion versicolor
COI
ML tree
0.1
