# Supplementary material for: An unexpected clade of South American ground beetles (Coleoptera, Carabidae, Bembidion)
Source: Zookeys. 2014 Jun 17;(416):113–55. doi: 10.3897/zookeys.416.7706 (PMC4109512; doi:10.3897/zookeys.416.7706)
Supplement: Supplementary material 4 — Images of maximum likelihood bootstrap trees [file zookeys-416-113-s004.pdf]

Supporting Information S4

This file shows the maximum likelihood bootstrap trees for the concatenated, 7-gene matrices as well as each individual gene. Each figure is labeled to indicate the nature of that data analyzed for that tree. Numbers on each branch are the frequencies of that clade in the bootstrap replicates, expressed as a percentage.

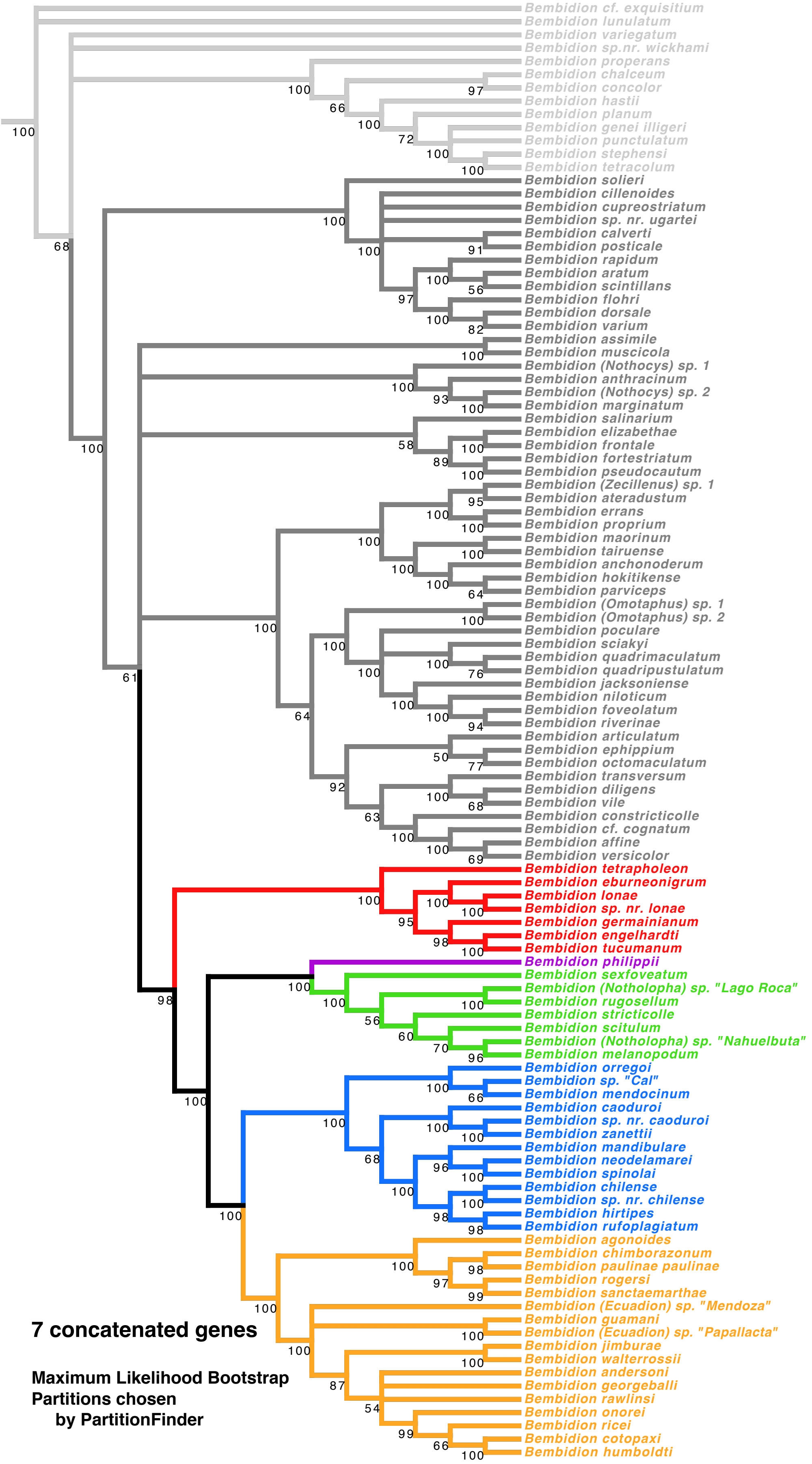
Bembidion cf. exquisitium
Bembidion lunulatum
Bembidion variegatum
Bembidion sp.nr. wickhami
Bembidion properans
Bembidion chalceum
Bembidion concolor
Bembidion hastii
Bembidion planum
Bembidion genei illigeri
Bembidion punctulatum
Bembidion stephensi
Bembidion tetracolum
Bembidion solieri
Bembidion cillenoides
Bembidion cupreostriatum
Bembidion sp. nr. ugartei
Bembidion calverti
Bembidion posticale
Bembidion rapidum
Bembidion aratum
Bembidion scintillans
Bembidion flohri
Bembidion dorsale
Bembidion varium
Bembidion assimile
Bembidion muscicola
Bembidion (Nothocys) sp. 1
Bembidion anthracinum
Bembidion (Nothocys) sp. 2
Bembidion marginatum
Bembidion salinarium
Bembidion elizabethae
Bembidion frontale
Bembidion fortestriatum
Bembidion pseudocautum
Bembidion (Zecillenus) sp. 1
Bembidion ateradustum
Bembidion errans
Bembidion proprium
Bembidion maorinum
Bembidion tairuense
Bembidion anchonoderum
Bembidion hokitikense
Bembidion parviceps
Bembidion (Omotaphus) sp. 1
Bembidion (Omotaphus) sp. 2
Bembidion poculare
Bembidion sciakyi
Bembidion quadrimaculatum
Bembidion quadripustulatum
Bembidion jacksoniense
Bembidion niloticum
Bembidion foveolatum
Bembidion riverinae
Bembidion articulatum
Bembidion ephippium
Bembidion octomaculatum
Bembidion transversum
Bembidion diligens
Bembidion vile
Bembidion constricticolle
Bembidion cf. cognatum
Bembidion affine
Bembidion versicolor
Bembidion tetrapholeon
Bembidion eburneonigrum
Bembidion lonae
Bembidion sp. nr. lonae
Bembidion germainianum
Bembidion engelhardti
Bembidion tucumanum
Bembidion philippii
Bembidion sexfoveatum
Bembidion (Notholopha) sp. "Lago Roca"
Bembidion rugosellum
Bembidion stricticolle
Bembidion scitulum
Bembidion (Notholopha) sp. "Nahuelbuta"
Bembidion melanopodum
Bembidion orregoi
Bembidion sp. "Cal"
Bembidion mendocinum
Bembidion caoduroi
Bembidion sp. nr. caoduroi
Bembidion zanettii
Bembidion mandibulare
Bembidion neodelamarei
Bembidion spinolai
Bembidion chilense
Bembidion sp. nr. chilense
Bembidion hirtipes
Bembidion rufoplagiatum
Bembidion agonoides
Bembidion chimborazonum
Bembidion paulinae paulinae
Bembidion rogersi
Bembidion sanctaemarthae
Bembidion (Ecuadion) sp. "Mendoza"
Bembidion guamani
Bembidion (Ecuadion) sp. "Papallacta"
Bembidion jimburae
Bembidion walterrossii
Bembidion andersoni
Bembidion georgeballi
Bembidion rawlinsi
Bembidion onorei
Bembidion ricei
Bembidion cotopaxi
Bembidion humboldti
7 concatenated genes
Maximum Likelihood Bootstrap
Partitions chosen
by PartitionFinder

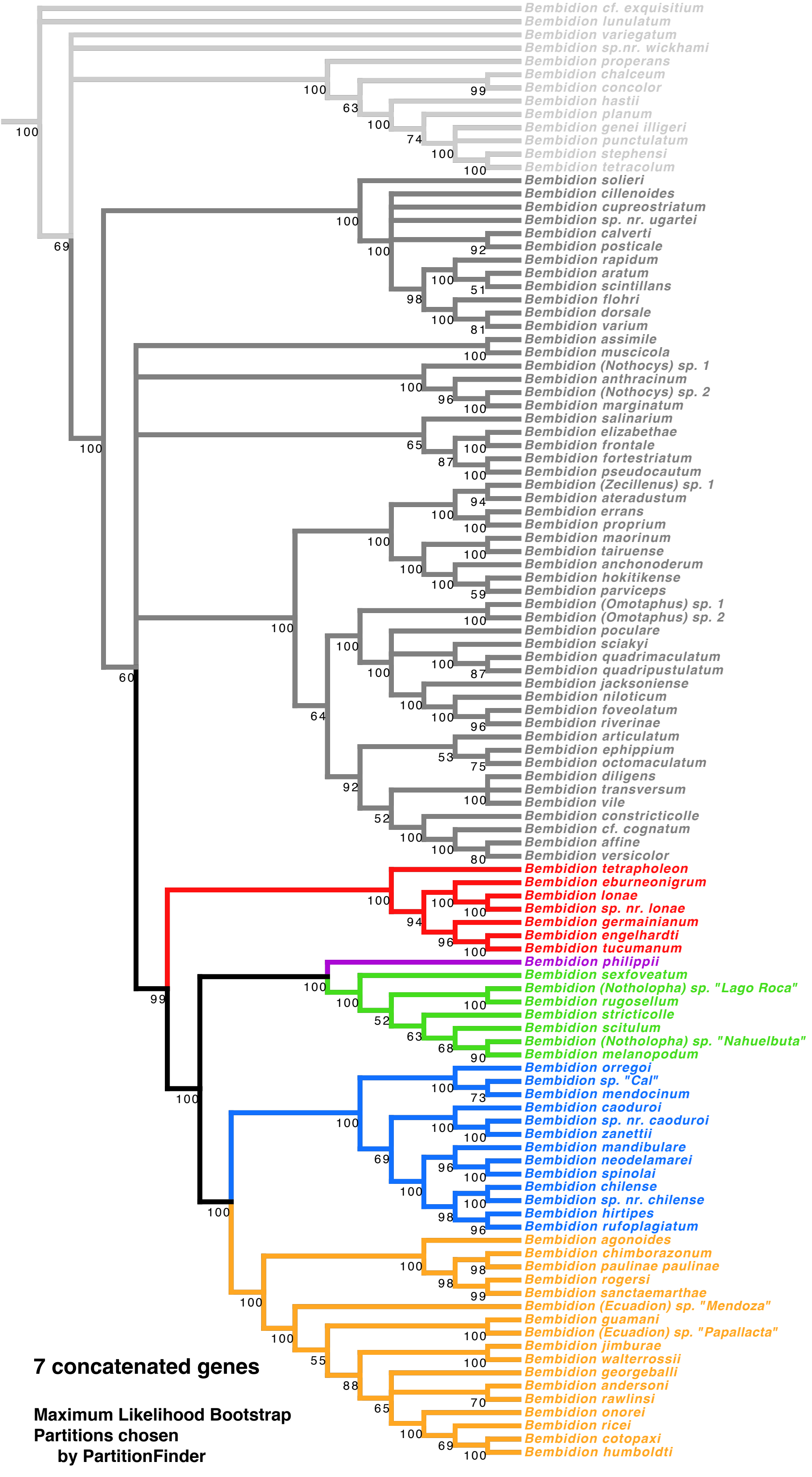
Bembidion cf. exquisitium
Bembidion lunulatum
Bembidion variegatum
Bembidion sp.nr. wickhami
Bembidion properans
Bembidion chalceum
Bembidion concolor
Bembidion hastii
Bembidion planum
Bembidion genei illigeri
Bembidion punctulatum
Bembidion stephensi
Bembidion tetracolum
Bembidion solieri
Bembidion cillenoides
Bembidion cupreostriatum
Bembidion sp. nr. ugartei
Bembidion calverti
Bembidion posticale
Bembidion rapidum
Bembidion aratum
Bembidion scintillans
Bembidion flohri
Bembidion dorsale
Bembidion varium
Bembidion assimile
Bembidion muscicola
Bembidion (Nothocys) sp. 1
Bembidion anthracinum
Bembidion (Nothocys) sp. 2
Bembidion marginatum
Bembidion salinarium
Bembidion elizabethae
Bembidion frontale
Bembidion fortestriatum
Bembidion pseudocautum
Bembidion (Zecillenus) sp. 1
Bembidion ateradustum
Bembidion errans
Bembidion proprium
Bembidion maorinum
Bembidion tairuense
Bembidion anchonoderum
Bembidion hokitikense
Bembidion parviceps
Bembidion (Omotaphus) sp. 1
Bembidion (Omotaphus) sp. 2
Bembidion poculare
Bembidion sciakyi
Bembidion quadrimaculatum
Bembidion quadripustulatum
Bembidion jacksoniense
Bembidion niloticum
Bembidion foveolatum
Bembidion riverinae
Bembidion articulatum
Bembidion ephippium
Bembidion octomaculatum
Bembidion diligens
Bembidion transversum
Bembidion vile
Bembidion constricticolle
Bembidion cf. cognatum
Bembidion affine
Bembidion versicolor
Bembidion tetrapholeon
Bembidion eburneonigrum
Bembidion lonae
Bembidion sp. nr. lonae
Bembidion germainianum
Bembidion engelhardti
Bembidion tucumanum
Bembidion philippii
Bembidion sexfoveatum
Bembidion (Notholopha) sp. "Lago Roca"
Bembidion rugosellum
Bembidion stricticolle
Bembidion scitulum
Bembidion (Notholopha) sp. "Nahuelbuta"
Bembidion melanopodum
Bembidion orregoi
Bembidion sp. "Cal"
Bembidion mendocinum
Bembidion caoduroi
Bembidion sp. nr. caoduroi
Bembidion zanettii
Bembidion mandibulare
Bembidion neodelamarei
Bembidion spinolai
Bembidion chilense
Bembidion sp. nr. chilense
Bembidion hirtipes
Bembidion rufoplagiatum
Bembidion agonoides
Bembidion chimborazonum
Bembidion paulinae paulinae
Bembidion rogersi
Bembidion sanctaemarthae
Bembidion (Ecuadion) sp. "Mendoza"
Bembidion guamani
Bembidion (Ecuadion) sp. "Papallacta"
Bembidion jimburae
Bembidion walterrossii
Bembidion georgeballi
Bembidion andersoni
Bembidion rawlinsi
Bembidion onorei
Bembidion ricei
Bembidion cotopaxi
Bembidion humboldti
7 concatenated genes
Maximum Likelihood Bootstrap
Partitions chosen
by PartitionFinder

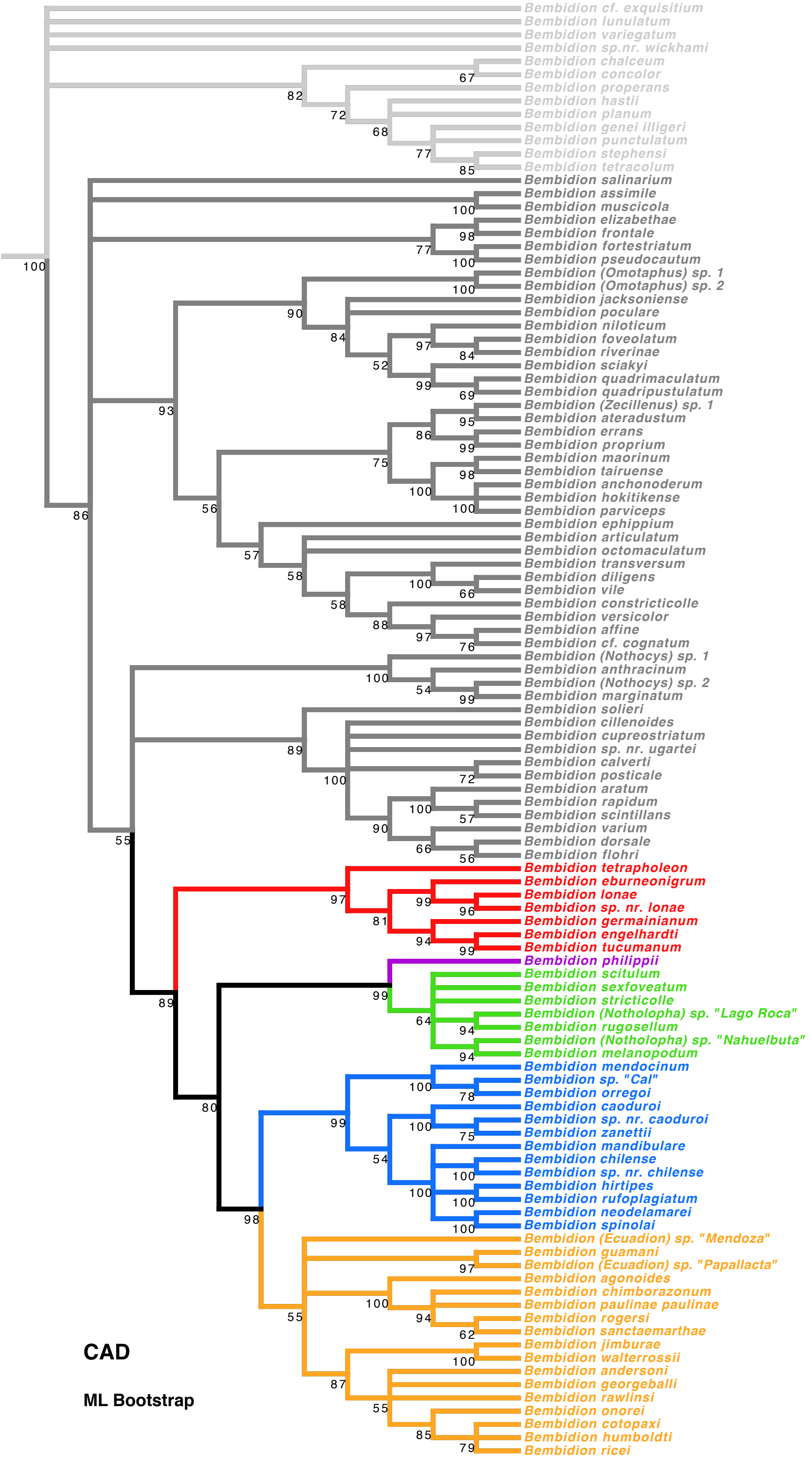
Bembidion cf. exquisitium
Bembidion lunulatum
Bembidion variegatum
Bembidion sp.nr. wickhami
Bembidion chalceum
Bembidion concolor
Bembidion properans
Bembidion hastii
Bembidion planum
Bembidion genei illigeri
Bembidion punctulatum
Bembidion stephensi
Bembidion tetracolum
Bembidion salinarium
Bembidion assimile
Bembidion muscicola
Bembidion elizabethae
Bembidion frontale
Bembidion fortestriatum
Bembidion pseudocautum
Bembidion (Omotaphus) sp. 1
Bembidion (Omotaphus) sp. 2
Bembidion jacksoniense
Bembidion poculare
Bembidion niloticum
Bembidion foveolatum
Bembidion riverinae
Bembidion sciakyi
Bembidion quadrimaculatum
Bembidion quadripustulatum
Bembidion (Zecillenus) sp. 1
Bembidion ateradustum
Bembidion errans
Bembidion proprium
Bembidion maorinum
Bembidion tairuense
Bembidion anchonoderum
Bembidion hokitikense
Bembidion parviceps
Bembidion ephippium
Bembidion articulatum
Bembidion octomaculatum
Bembidion transversum
Bembidion diligens
Bembidion vile
Bembidion constricticolle
Bembidion versicolor
Bembidion affine
Bembidion cf. cognatum
Bembidion (Nothocys) sp. 1
Bembidion anthracinum
Bembidion (Nothocys) sp. 2
Bembidion marginatum
Bembidion solieri
Bembidion cillenoides
Bembidion cupreostriatum
Bembidion sp. nr. ugartei
Bembidion calverti
Bembidion posticale
Bembidion aratum
Bembidion rapidum
Bembidion scintillans
Bembidion varium
Bembidion dorsale
Bembidion flohri
Bembidion tetrapholeon
Bembidion eburneonigrum
Bembidion lonae
Bembidion sp. nr. lonae
Bembidion germainianum
Bembidion engelhardti
Bembidion tucumanum
Bembidion philippii
Bembidion scitulum
Bembidion sexfoveatum
Bembidion stricticolle
Bembidion (Notholopha) sp. "Lago Roca"
Bembidion rugosellum
Bembidion (Notholopha) sp. "Nahuelbuta"
Bembidion melanopodum
Bembidion mendocinum
Bembidion sp. "Cal"
Bembidion orregoi
Bembidion caoduroi
Bembidion sp. nr. caoduroi
Bembidion zanettii
Bembidion mandibulare
Bembidion chilense
Bembidion sp. nr. chilense
Bembidion hirtipes
Bembidion rufoplagiatum
Bembidion neodelamarei
Bembidion spinolai
Bembidion (Ecuadion) sp. "Mendoza"
Bembidion guamani
Bembidion (Ecuadion) sp. "Papallacta"
Bembidion agonoides
Bembidion chimborazonum
Bembidion paulinae paulinae
Bembidion rogersi
Bembidion sanctaemarthae
Bembidion jimburae
Bembidion walterrossii
Bembidion andersoni
Bembidion georgeballi
Bembidion rawlinsi
Bembidion onorei
Bembidion cotopaxi
Bembidion humboldti
Bembidion ricei
CAD
ML Bootstrap

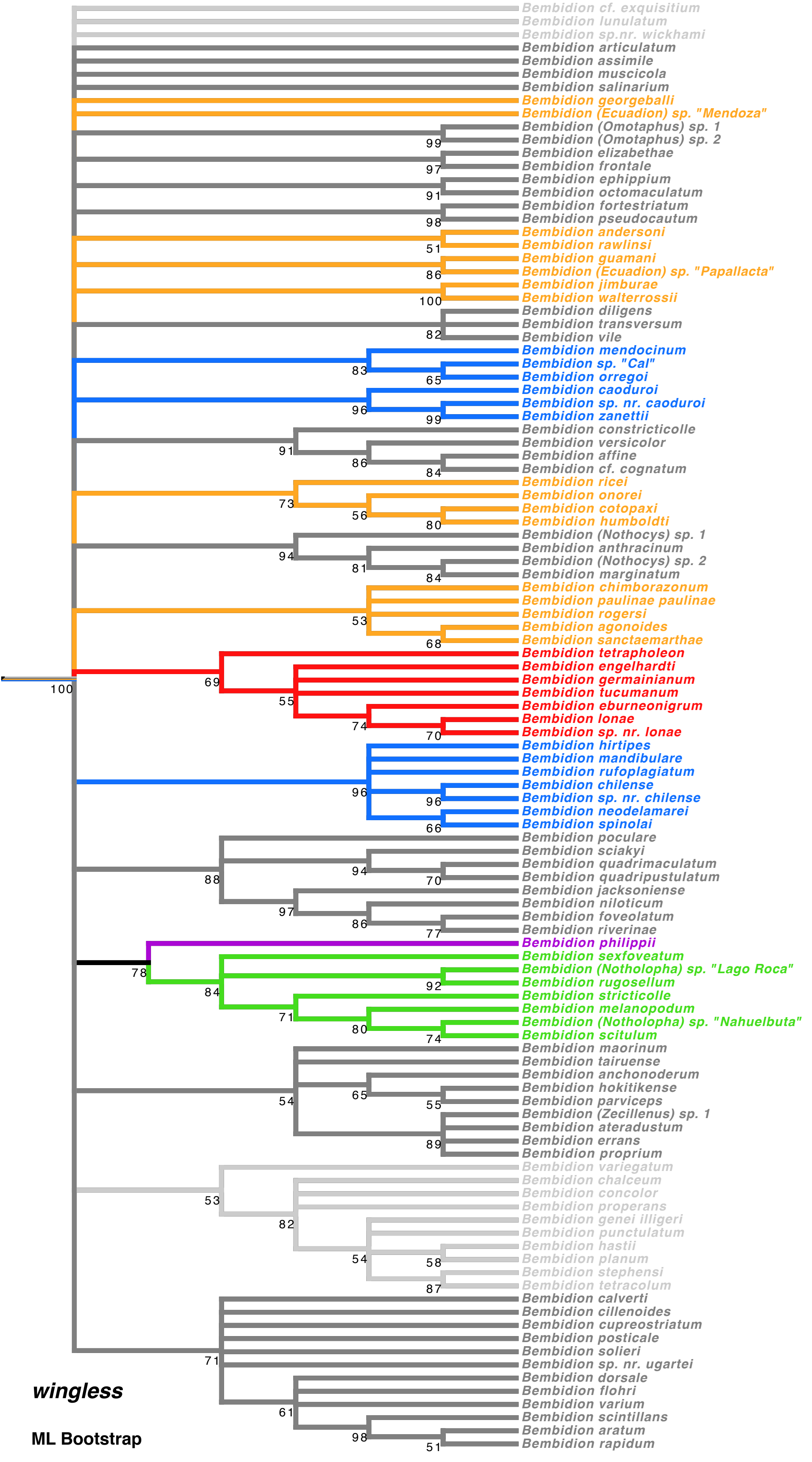
Bembidion cf. exquisitium
Bembidion lunulatum
Bembidion sp.nr. wickhami
Bembidion articulatum
Bembidion assimile
Bembidion muscicola
Bembidion salinarium
Bembidion georgeballi
Bembidion (Ecuadion) sp. "Mendoza"
Bembidion (Omotaphus) sp. 1
Bembidion (Omotaphus) sp. 2
Bembidion elizabethae
Bembidion frontale
Bembidion ephippium
Bembidion octomaculatum
Bembidion fortestriatum
Bembidion pseudocautum
Bembidion andersoni
Bembidion rawlinsi
Bembidion guamani
Bembidion (Ecuadion) sp. "Papallacta"
Bembidion jimburae
Bembidion walterrossii
Bembidion diligens
Bembidion transversum
Bembidion vile
Bembidion mendocinum
Bembidion sp. "Cal"
Bembidion orregoi
Bembidion caoduroi
Bembidion sp. nr. caoduroi
Bembidion zanettii
Bembidion constricticolle
Bembidion versicolor
Bembidion affine
Bembidion cf. cognatum
Bembidion ricei
Bembidion onorei
Bembidion cotopaxi
Bembidion humboldti
Bembidion (Nothocys) sp. 1
Bembidion anthracinum
Bembidion (Nothocys) sp. 2
Bembidion marginatum
Bembidion chimborazonum
Bembidion paulinae paulinae
Bembidion rogersi
Bembidion agonoides
Bembidion sanctaemarthae
Bembidion tetrapholeon
Bembidion engelhardti
Bembidion germainianum
Bembidion tucumanum
Bembidion eburneonigrum
Bembidion lonae
Bembidion sp. nr. lonae
Bembidion hirtipes
Bembidion mandibulare
Bembidion rufoplagiatum
Bembidion chilense
Bembidion sp. nr. chilense
Bembidion neodelamarei
Bembidion spinolai
Bembidion poculare
Bembidion sciakyi
Bembidion quadrimaculatum
Bembidion quadripustulatum
Bembidion jacksoniense
Bembidion niloticum
Bembidion foveolatum
Bembidion riverinae
Bembidion philippii
Bembidion sexfoveatum
Bembidion (Notholopha) sp. "Lago Roca"
Bembidion rugosellum
Bembidion stricticolle
Bembidion melanopodum
Bembidion (Notholopha) sp. "Nahuelbuta"
Bembidion scitulum
Bembidion maorinum
Bembidion tairuense
Bembidion anchonoderum
Bembidion hokitikense
Bembidion parviceps
Bembidion (Zecillenus) sp. 1
Bembidion ateradustum
Bembidion errans
Bembidion proprium
Bembidion variegatum
Bembidion chalceum
Bembidion concolor
Bembidion properans
Bembidion genei illigeri
Bembidion punctulatum
Bembidion hastii
Bembidion planum
Bembidion stephensi
Bembidion tetracolum
Bembidion calverti
Bembidion cillenoides
Bembidion cupreostriatum
Bembidion posticale
Bembidion solieri
Bembidion sp. nr. ugartei
Bembidion dorsale
Bembidion flohri
Bembidion varium
Bembidion scintillans
Bembidion aratum
Bembidion rapidum
wingless
ML Bootstrap

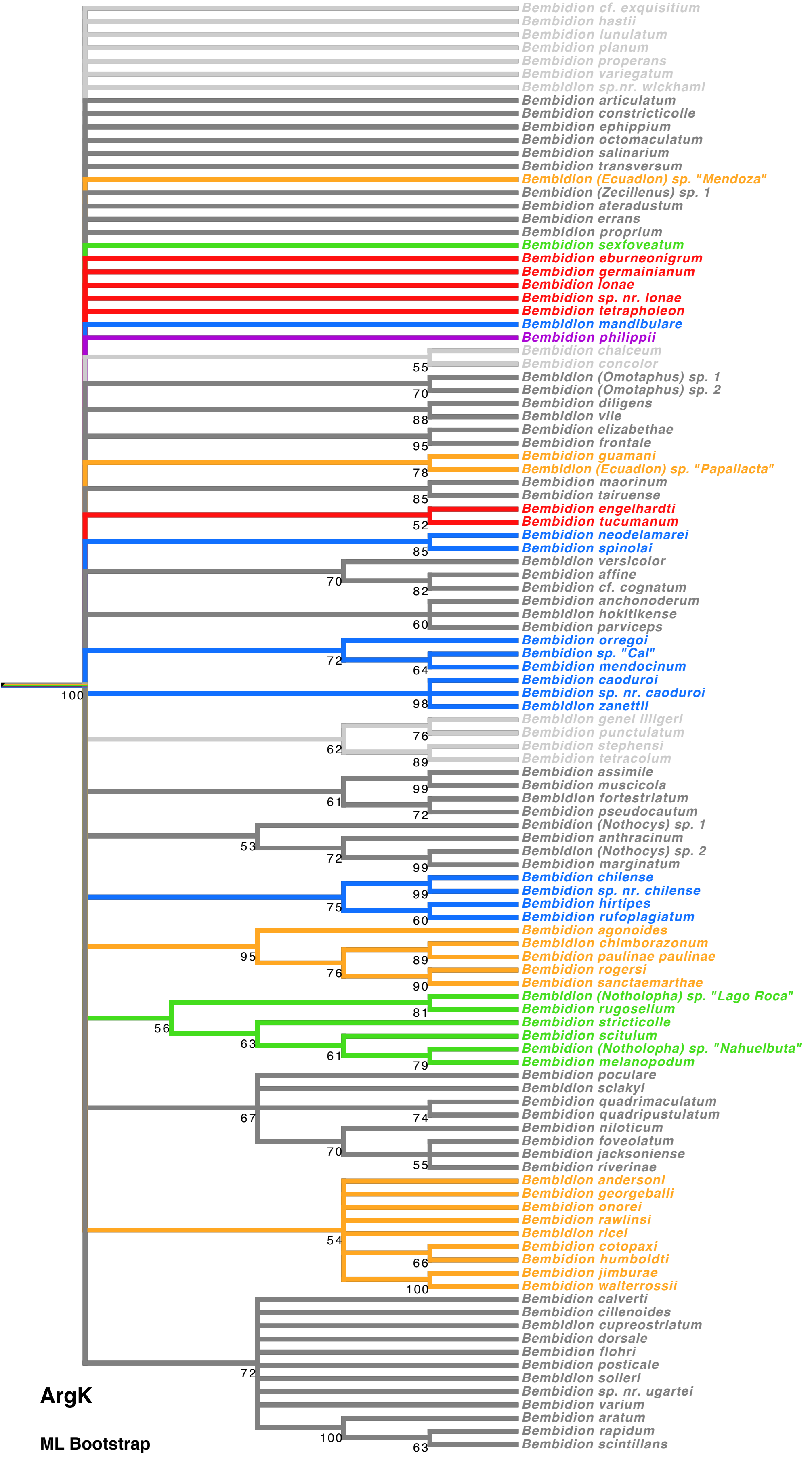
Bembidion cf. exquisitium
Bembidion hastii
Bembidion lunulatum
Bembidion planum
Bembidion properans
Bembidion variegatum
Bembidion sp.nr. wickhami
Bembidion articulatum
Bembidion constricticolle
Bembidion ephippium
Bembidion octomaculatum
Bembidion salinarium
Bembidion transversum
Bembidion (Ecuadion) sp. "Mendoza"
Bembidion (Zecillenus) sp. 1
Bembidion ateradustum
Bembidion errans
Bembidion proprium
Bembidion sexfoveatum
Bembidion eburneonigrum
Bembidion germainianum
Bembidion lonae
Bembidion sp. nr. lonae
Bembidion tetrapholeon
Bembidion mandibulare
Bembidion philippii
Bembidion chalceum
Bembidion concolor
Bembidion (Omotaphus) sp. 1
Bembidion (Omotaphus) sp. 2
Bembidion diligens
Bembidion vile
Bembidion elizabethae
Bembidion frontale
Bembidion guamani
Bembidion (Ecuadion) sp. "Papallacta"
Bembidion maorinum
Bembidion tairuense
Bembidion engelhardti
Bembidion tucumanum
Bembidion neodelamarei
Bembidion spinolai
Bembidion versicolor
Bembidion affine
Bembidion cf. cognatum
Bembidion anchonoderum
Bembidion hokitikense
Bembidion parviceps
Bembidion orregoi
Bembidion sp. "Cal"
Bembidion mendocinum
Bembidion caoduroi
Bembidion sp. nr. caoduroi
Bembidion zanettii
Bembidion genei illigeri
Bembidion punctulatum
Bembidion stephensi
Bembidion tetracolum
Bembidion assimile
Bembidion muscicola
Bembidion fortestriatum
Bembidion pseudocautum
Bembidion (Nothocys) sp. 1
Bembidion anthracinum
Bembidion (Nothocys) sp. 2
Bembidion marginatum
Bembidion chilense
Bembidion sp. nr. chilense
Bembidion hirtipes
Bembidion rufoplagiatum
Bembidion agonoides
Bembidion chimborazonum
Bembidion paulinae paulinae
Bembidion rogersi
Bembidion sanctaemarthae
Bembidion (Notholopha) sp. "Lago Roca"
Bembidion rugosellum
Bembidion stricticolle
Bembidion scitulum
Bembidion (Notholopha) sp. "Nahuelbuta"
Bembidion melanopodum
Bembidion poculare
Bembidion sciakyi
Bembidion quadrimaculatum
Bembidion quadripustulatum
Bembidion niloticum
Bembidion foveolatum
Bembidion jacksoniense
Bembidion riverinae
Bembidion andersoni
Bembidion georgeballi
Bembidion onorei
Bembidion rawlinsi
Bembidion ricei
Bembidion cotopaxi
Bembidion humboldti
Bembidion jimburae
Bembidion walterrossii
Bembidion calverti
Bembidion cillenoides
Bembidion cupreostriatum
Bembidion dorsale
Bembidion flohri
Bembidion posticale
Bembidion solieri
Bembidion sp. nr. ugartei
Bembidion varium
Bembidion aratum
Bembidion rapidum
Bembidion scintillans
55
70
88
95
78
85
52
85
70
82
60
72
64
100
98
76
62
89
99
61
72
53
72
99
99
75
60
95
89
76
90
81
56
63
61
79
67
74
70
55
54
66
100
72
100
63
ArgK
ML Bootstrap

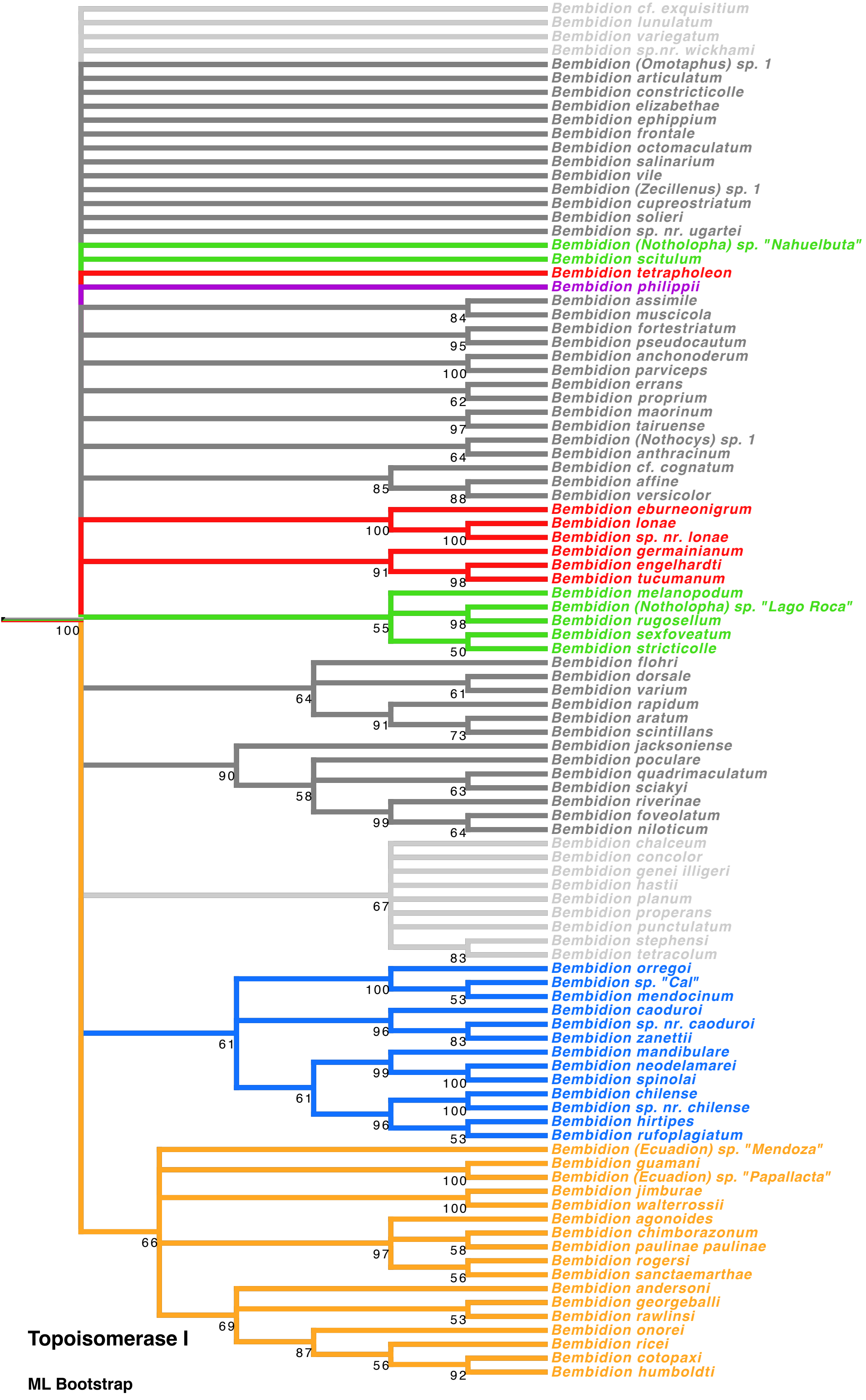
Bembidion cf. exquisitium
Bembidion lunulatum
Bembidion variegatum
Bembidion sp.nr. wickhami
Bembidion (Omotaphus) sp. 1
Bembidion articulatum
Bembidion constricticolle
Bembidion elizabethae
Bembidion ephippium
Bembidion frontale
Bembidion octomaculatum
Bembidion salinarium
Bembidion vile
Bembidion (Zecillenus) sp. 1
Bembidion cupreostriatum
Bembidion solieri
Bembidion sp. nr. ugartei
Bembidion (Notholopha) sp. "Nahuelbuta"
Bembidion scitulum
Bembidion tetrapholeon
Bembidion philippii
Bembidion assimile
Bembidion muscicola
Bembidion fortestriatum
Bembidion pseudocautum
Bembidion anchonoderum
Bembidion parviceps
Bembidion errans
Bembidion proprium
Bembidion maorinum
Bembidion tairuense
Bembidion (Nothocys) sp. 1
Bembidion anthracinum
Bembidion cf. cognatum
Bembidion affine
Bembidion versicolor
Bembidion eburneonigrum
Bembidion lonae
Bembidion sp. nr. lonae
Bembidion germainianum
Bembidion engelhardti
Bembidion tucumanum
Bembidion melanopodum
Bembidion (Notholopha) sp. "Lago Roca"
Bembidion rugosellum
Bembidion sexfoveatum
Bembidion stricticolle
Bembidion flohri
Bembidion dorsale
Bembidion varium
Bembidion rapidum
Bembidion aratum
Bembidion scintillans
Bembidion jacksoniense
Bembidion poculare
Bembidion quadrimaculatum
Bembidion sciakyi
Bembidion riverinae
Bembidion foveolatum
Bembidion niloticum
Bembidion chalceum
Bembidion concolor
Bembidion genei illigeri
Bembidion hastii
Bembidion planum
Bembidion properans
Bembidion punctulatum
Bembidion stephensi
Bembidion tetracolum
Bembidion orregoi
Bembidion sp. "Cal"
Bembidion mendocinum
Bembidion caoduroi
Bembidion sp. nr. caoduroi
Bembidion zanettii
Bembidion mandibulare
Bembidion neodelamarei
Bembidion spinolai
Bembidion chilense
Bembidion sp. nr. chilense
Bembidion hirtipes
Bembidion rufoplagiatum
Bembidion (Ecuadion) sp. "Mendoza"
Bembidion guamani
Bembidion (Ecuadion) sp. "Papallacta"
Bembidion jimburae
Bembidion walterrossii
Bembidion agonoides
Bembidion chimborazonum
Bembidion paulinae paulinae
Bembidion rogersi
Bembidion sanctaemarthae
Bembidion andersoni
Bembidion georgeballi
Bembidion rawlinsi
Bembidion onorei
Bembidion ricei
Bembidion cotopaxi
Bembidion humboldti
Topoisomerase I
ML Bootstrap

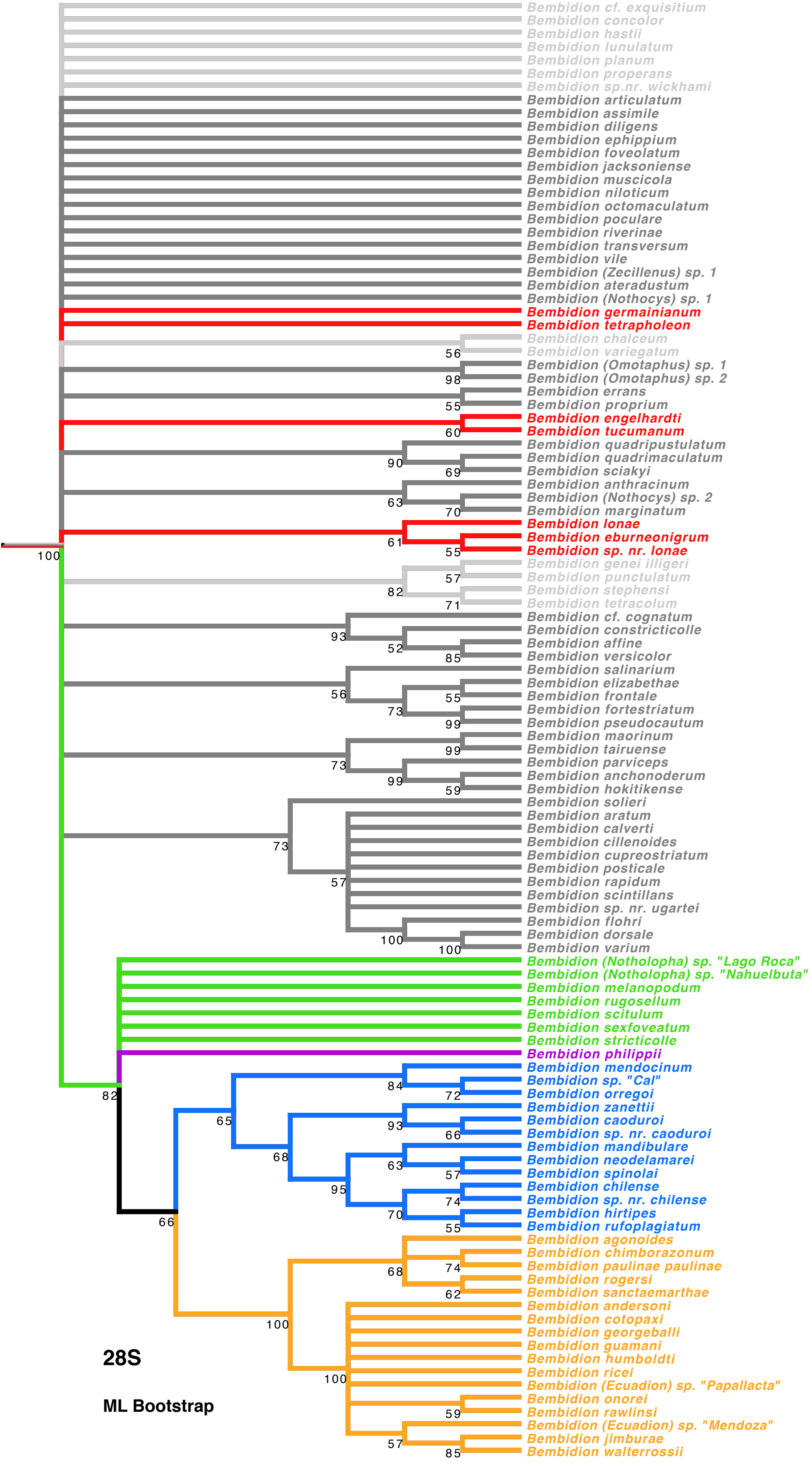

Bembidion cf. exquisitium
Bembidion concolor
Bembidion hastii
Bembidion lunulatum
Bembidion planum
Bembidion properans
Bembidion sp.nr. wickhami
Bembidion articulatum
Bembidion assimile
Bembidion diligens
Bembidion ephippium
Bembidion foveolatum
Bembidion jacksoniense
Bembidion muscicola
Bembidion niloticum
Bembidion octomaculatum
Bembidion poculare
Bembidion riverinae
Bembidion transversum
Bembidion vile
Bembidion (Zecillenus) sp. 1
Bembidion ateradustum
Bembidion (Nothocys) sp. 1
Bembidion germainianum
Bembidion tetrapholeon
Bembidion chalceum
56
Bembidion variegatum
Bembidion (Omotaphus) sp. 1
98
Bembidion (Omotaphus) sp. 2
Bembidion errans
55
Bembidion proprium
Bembidion engelhardti
60
Bembidion tucumanum
Bembidion quadripustulatum
90
Bembidion quadrimaculatum
69
Bembidion sciakyi
Bembidion anthracinum
63
Bembidion (Nothocys) sp. 2
70
Bembidion marginatum
Bembidion lonae
61
Bembidion eburneonigrum
55
Bembidion sp. nr. lonae
100
Bembidion genei illigeri
57
Bembidion punctulatum
82
Bembidion stephensi
71
Bembidion tetracolum
Bembidion cf. cognatum
93
Bembidion constricticolle
52
Bembidion affine
85
Bembidion versicolor
Bembidion salinarium
Bembidion elizabethae
56
Bembidion frontale
55
73
Bembidion fortestriatum
99
Bembidion pseudocautum
Bembidion maorinum
99
Bembidion tairuense
73
Bembidion parviceps
99
Bembidion anchonoderum
59
Bembidion hokitikense
Bembidion solieri
Bembidion aratum
Bembidion calverti
73
Bembidion cillenoides
Bembidion cupreostriatum
Bembidion posticale
57
Bembidion rapidum
Bembidion scintillans
Bembidion sp. nr. ugartei
Bembidion flohri
100
Bembidion dorsale
100
Bembidion varium
Bembidion (Notholopha) sp. "Lago Roca"
Bembidion (Notholopha) sp. "Nahuelbuta"
Bembidion melanopodum
Bembidion rugosellum
Bembidion scitulum
Bembidion sexfoveatum
Bembidion stricticolle
Bembidion philippii
Bembidion mendocinum
84
Bembidion sp. "Cal"
82
72
Bembidion orregoi
65
Bembidion zanettii
93
Bembidion caoduroi
66
Bembidion sp. nr. caoduroi
68
Bembidion mandibulare
63
Bembidion neodelamarei
57
Bembidion spinolai
95
Bembidion chilense
74
Bembidion sp. nr. chilense
70
Bembidion hirtipes
66
55
Bembidion rufoplagiatum
Bembidion agonoides
Bembidion chimborazonum
74
68
Bembidion paulinae paulinae
Bembidion rogersi
62
Bembidion sanctaemarthae
Bembidion andersoni
100
Bembidion cotopaxi
Bembidion georgeballi
Bembidion guamani
Bembidion humboldti
100
Bembidion ricei
Bembidion (Ecuadion) sp. "Papallacta"
Bembidion onorei
59
Bembidion rawlinsi
Bembidion (Ecuadion) sp. "Mendoza"
57
Bembidion jimburae
85
Bembidion walterrossii
28S
ML Bootstrap

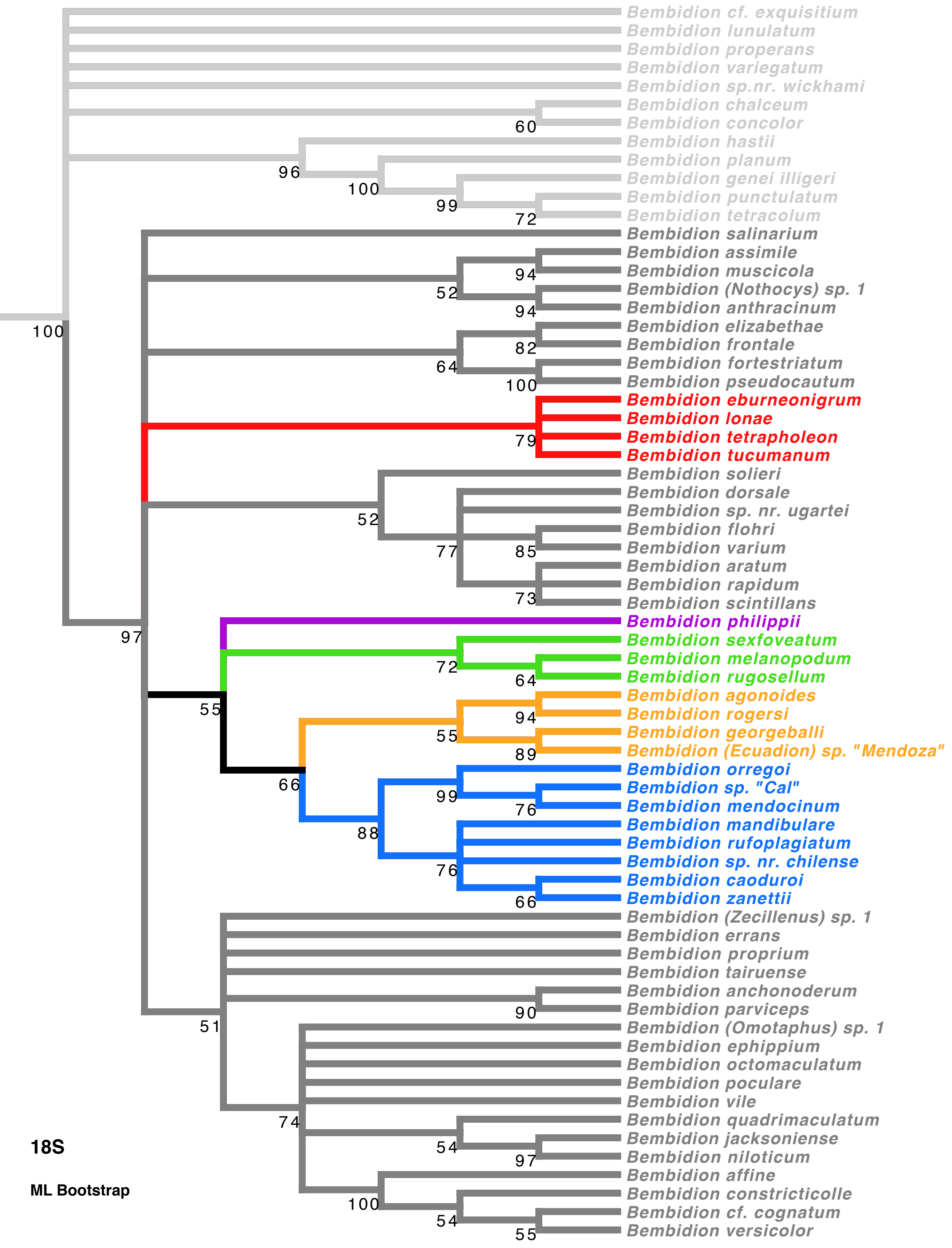

Bembidion cf. exquisitium
Bembidion lunulatum
Bembidion properans
Bembidion variegatum
Bembidion sp.nr. wickhami
Bembidion chalceum
Bembidion concolor
Bembidion hastii
Bembidion planum
Bembidion genei illigeri
Bembidion punctulatum
Bembidion tetracolum
Bembidion salinarium
Bembidion assimile
Bembidion muscicola
Bembidion (Nothocys) sp. 1
Bembidion anthracinum
Bembidion elizabethae
Bembidion frontale
Bembidion fortestriatum
Bembidion pseudocautum
Bembidion eburneonigrum
Bembidion lonae
Bembidion tetrapholeon
Bembidion tucumanum
Bembidion solieri
Bembidion dorsale
Bembidion sp. nr. ugartei
Bembidion flohri
Bembidion varium
Bembidion aratum
Bembidion rapidum
Bembidion scintillans
Bembidion philippii
Bembidion sexfoveatum
Bembidion melanopodum
Bembidion rugosellum
Bembidion agonoides
Bembidion rogersi
Bembidion georgeballi
Bembidion (Ecuadion) sp. "Mendoza"
Bembidion orregoi
Bembidion sp. "Cal"
Bembidion mendocinum
Bembidion mandibulare
Bembidion rufoplagiatum
Bembidion sp. nr. chilense
Bembidion caoduroi
Bembidion zanettii
Bembidion (Zecillenus) sp. 1
Bembidion errans
Bembidion proprium
Bembidion tairuense
Bembidion anchonoderum
Bembidion parviceps
Bembidion (Omotaphus) sp. 1
Bembidion ephippium
Bembidion octomaculatum
Bembidion poculare
Bembidion vile
Bembidion quadrimaculatum
Bembidion jacksoniense
Bembidion niloticum
Bembidion affine
Bembidion constricticolle
Bembidion cf. cognatum
Bembidion versicolor
18S
ML Bootstrap

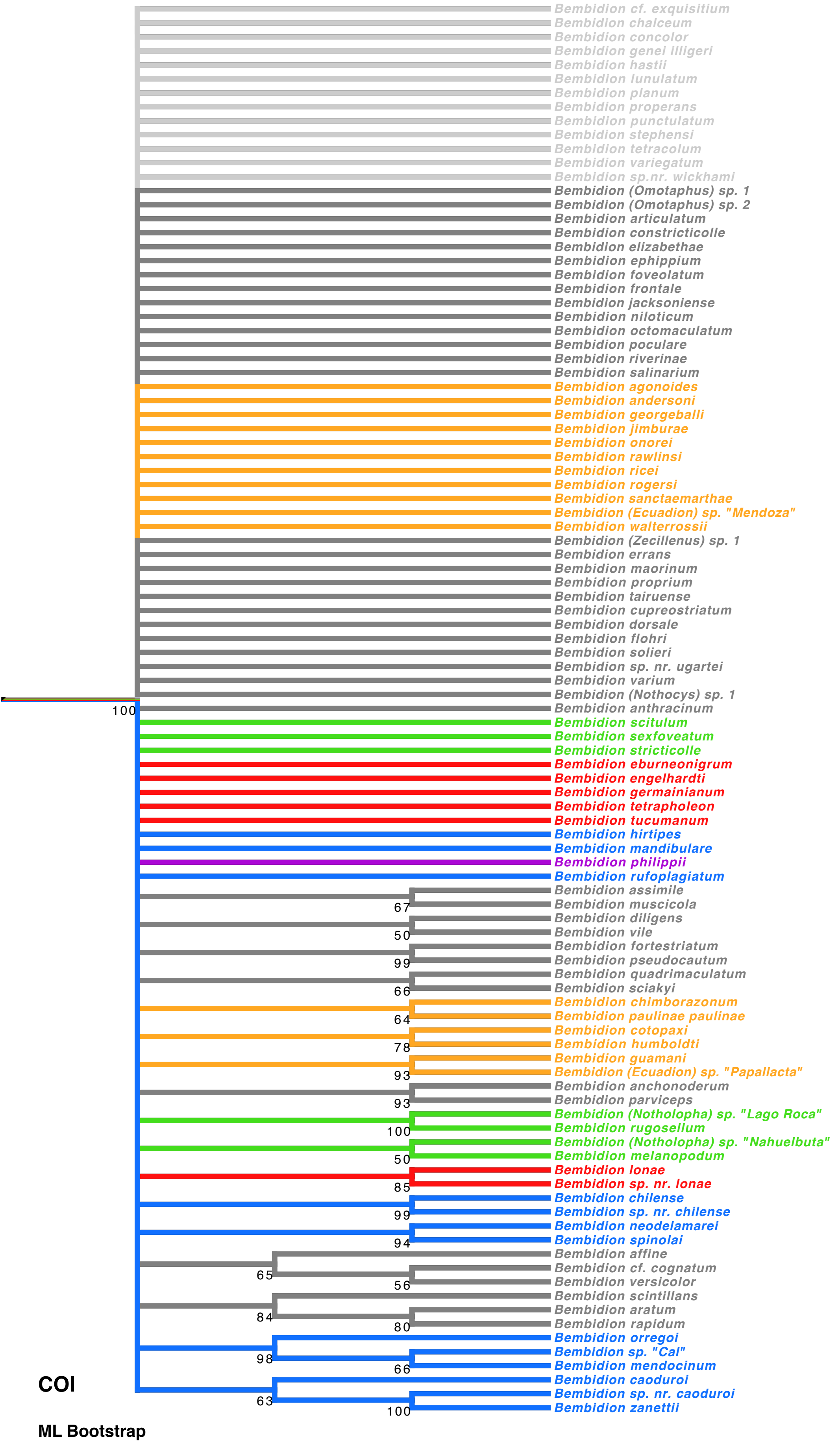
Bembidion cf. exquisitium
Bembidion chalceum
Bembidion concolor
Bembidion genei illigeri
Bembidion hastii
Bembidion lunulatum
Bembidion planum
Bembidion properans
Bembidion punctulatum
Bembidion stephensi
Bembidion tetracolum
Bembidion variegatum
Bembidion sp.nr. wickhami
Bembidion (Omotaphus) sp. 1
Bembidion (Omotaphus) sp. 2
Bembidion articulatum
Bembidion constricticolle
Bembidion elizabethae
Bembidion ephippium
Bembidion foveolatum
Bembidion frontale
Bembidion jacksoniense
Bembidion niloticum
Bembidion octomaculatum
Bembidion poculare
Bembidion riverinae
Bembidion salinarium
Bembidion agonoides
Bembidion andersoni
Bembidion georgeballi
Bembidion jimburae
Bembidion onorei
Bembidion rawlinsi
Bembidion ricei
Bembidion rogersi
Bembidion sanctaemarthae
Bembidion (Ecuadion) sp. "Mendoza"
Bembidion walterrossii
Bembidion (Zecillenus) sp. 1
Bembidion errans
Bembidion maorinum
Bembidion proprium
Bembidion tairuense
Bembidion cupreostriatum
Bembidion dorsale
Bembidion flohri
Bembidion solieri
Bembidion sp. nr. ugartei
Bembidion varium
Bembidion (Nothocys) sp. 1
Bembidion anthracinum
100
Bembidion scitulum
Bembidion sexfoveatum
Bembidion stricticolle
Bembidion eburneonigrum
Bembidion engelhardti
Bembidion germainianum
Bembidion tetrapholeon
Bembidion tucumanum
Bembidion hirtipes
Bembidion mandibulare
Bembidion philippii
Bembidion rufoplagiatum
Bembidion assimile
67
Bembidion muscicola
Bembidion diligens
50
Bembidion vile
Bembidion fortestriatum
99
Bembidion pseudocautum
Bembidion quadrimaculatum
66
Bembidion sciakyi
Bembidion chimborazonum
64
Bembidion paulinae paulinae
Bembidion cotopaxi
78
Bembidion humboldti
Bembidion guamani
93
Bembidion (Ecuadion) sp. "Papallacta"
Bembidion anchonoderum
93
Bembidion parviceps
Bembidion (Notholopha) sp. "Lago Roca"
100
Bembidion rugosellum
Bembidion (Notholopha) sp. "Nahuelbuta"
50
Bembidion melanopodum
Bembidion lonae
85
Bembidion sp. nr. lonae
Bembidion chilense
99
Bembidion sp. nr. chilense
Bembidion neodelamarei
94
Bembidion spinolai
Bembidion affine
65
Bembidion cf. cognatum
56
Bembidion versicolor
Bembidion scintillans
84
Bembidion aratum
80
Bembidion rapidum
Bembidion orregoi
98
Bembidion sp. "Cal"
66
Bembidion mendocinum
Bembidion caoduroi
63
Bembidion sp. nr. caoduroi
100
Bembidion zanettii
COI
ML Bootstrap
